# Nutritional markers of undiagnosed type 2 diabetes in adults: Findings of a machine learning analysis with external validation and benchmarking

**DOI:** 10.1371/journal.pone.0250832

**Published:** 2021-05-05

**Authors:** Kushan De Silva, Siew Lim, Aya Mousa, Helena Teede, Andrew Forbes, Ryan T. Demmer, Daniel Jönsson, Joanne Enticott

**Affiliations:** 1 Monash Centre for Health Research and Implementation, School of Public Health and Preventive Medicine, Faculty of Medicine, Nursing, and Health Sciences, Monash University, Clayton, Australia; 2 Biostatistics Unit, Division of Research Methodology, School of Public Health and Preventive Medicine, Faculty of Medicine, Nursing, and Health Sciences, Monash University, Melbourne, Australia; 3 Division of Epidemiology and Community Health, School of Public Health, University of Minnesota, Minneapolis, Minnesota, United States of America; 4 Mailman School of Public Health, Columbia University, New York, New York, United States of America; 5 Department of Periodontology, Faculty of Odontology, Malmö University, Malmö, Sweden; 6 Swedish Dental Service of Skane, Lund, Sweden; Taipei Medical University, TAIWAN

## Abstract

**Objectives:**

Using a nationally-representative, cross-sectional cohort, we examined nutritional markers of undiagnosed type 2 diabetes in adults via machine learning.

**Methods:**

A total of 16429 men and non-pregnant women ≥ 20 years of age were analysed from five consecutive cycles of the National Health and Nutrition Examination Survey. Cohorts from years 2013–2016 (n = 6673) was used for external validation. Undiagnosed type 2 diabetes was determined by a negative response to the question “Have you ever been told by a doctor that you have diabetes?” and a positive glycaemic response to one or more of the three diagnostic tests (HbA1c > 6.4% or FPG >125 mg/dl or 2-hr post-OGTT glucose > 200mg/dl). Following comprehensive literature search, 114 potential nutritional markers were modelled with 13 behavioural and 12 socio-economic variables. We tested three machine learning algorithms on original and resampled training datasets built using three resampling methods. From this, the derived 12 predictive models were validated on internal- and external validation cohorts. Magnitudes of associations were gauged through odds ratios in logistic models and variable importance in others. Models were benchmarked against the ADA diabetes risk test.

**Results:**

The prevalence of undiagnosed type 2 diabetes was 5.26%. Four best-performing models (AUROC range: 74.9%-75.7%) classified 39 markers of undiagnosed type 2 diabetes; 28 via one or more of the three best-performing non-linear/ensemble models and 11 uniquely by the logistic model. They comprised 14 nutrient-based, 12 anthropometry-based, 9 socio-behavioural, and 4 diet-associated markers. AUROC of all models were on a par with ADA diabetes risk test on both internal and external validation cohorts (*p*>0.05).

**Conclusions:**

Models performed comparably to the chosen benchmark. Novel behavioural markers such as the number of meals not prepared from home were revealed. This approach may be useful in nutritional epidemiology to unravel new associations with type 2 diabetes.

## Introduction

Diabetes is one of the most wide-spread non-communicable diseases in the world, which is expected to affect 552 million people by year 2030 [1]. Primary prevention of the most prevalent form of diabetes i.e. type 2 diabetes [2] is driven by healthy lifestyle-focussed interventions and policies [3, 4]. However, different principles and policies may underpin prevention and management of other less prevalent phenotypes such as type 1 diabetes [5], latent autoimmune diabetes in adults [6], or rare monogenic diabetes [7]. Nutritional aspects including food habits, dietary constituents, and anthropometric measures offer value since these are relatively easily modifiable at an individual level [8] compared to socio-economic factors such as income, education, or occupation, the modification of which would often require higher policy-level and broader societal interventions [9]. However, there is a dearth of nutritional information for optimising type 2 diabetes prevention [10]. Further studies are needed to deepen our understanding of dietary factors associated with type 2 diabetes risk and specific physiological and systemic pathways underlying those associations.

Extraneous factors such as cooking practices and food contaminants as well as individual metabolic heterogeneity such as variations in genetics, epigenetics, and microbiome may further confound diet-type 2 diabetes associations, resulting in even contradictory findings that are not uncommon in the literature [11]. As such, studies which model these associations should strive to adjust for these factors to derive meaningful evidence [12]. With increasingly available multidimensional big data and machine learning (ML) techniques, such precision nutrition approaches are needed to understand nutritional aetiopathogenesis of disease and to develop tailored programs [13]. Presently, ML is sparingly used in nutrition research [14], despite its promise and broadening applications in other areas of research including type 2 diabetes [15].

It should be noted that the current screening tools of type 2 diabetes are heavily hinged on non-modifiable markers such as age and family history, with less emphasis on modifiable, behavioural aspects including little to no nutritional inputs. The American Diabetes Association (ADA) type 2 diabetes risk test comprises age, gender, history of gestational diabetes mellitus (in women), family history of diabetes, history of hypertension, body mass index (BMI) and physical activity [16]. Similarly, the Australian type 2 diabetes risk assessment tool (AUSDRISK) incorporates age, gender, ethnicity/country of birth, family history of diabetes, history of hyperglycaemia and history of hypertension as well as some lifestyle or anthropometric factors such as smoking, physical activity, and waist circumference, and a single dietary question (frequency of fruit/vegetables intake) [17]. The Finnish diabetes risk score (FINDRISC) is derived from age, gender, history of hypertension, history of hyperglycaemia, family history of diabetes, immediate relatives with history of diabetes, BMI, waist circumference, physical activity and the frequency of fruit/vegetable intake [18]. It has been found that the available screening tools composed of a few known predictors result in the underdiagnosis of early dysglycaemia [19]. A study which assessed four type 2 diabetes risk assessment tools based on these few predictors reported of sub-standard performance and low external validity on new populations [20].

At present, evidence-based nutritional practices for primary prevention of type 2 diabetes in adults include lower consumption of dietary fat and energy as well as sufficient intake of dietary fibres (14 g fibres/1000 kcal) and whole-grain foods (equivalent to 50% of grain intake). These dietary practices should be combined with lifestyle interventions focussed on moderate weight loss (7% body weight) and steady exercises (150 minutes/week). Consumption of low glycaemic index (GI) foods enriched with fibres and nutrients is encouraged despite lack of direct evidence that low GI food per se prevents the onset of type 2 diabetes. Alcohol use is not recommended for individuals at high-risk of type 2 diabetes regardless of the beneficial effects associated with its moderate use revealed by observational studies [21].

A thorough understanding of the role of nutrition and its complex interplay with other factors in the natural history of type 2 diabetes is key to developing personalised prevention programs as well as managing overt diabetes [10]. Therefore, there is a need to explore opportunities to expand on and improve the existing sparse models to achieve higher predictive ability by incorporating more granular information on modifiable predictors of type 2 diabetes such as nutritional aspects. From a translational perspective, cost-effective, scalable markers derived from self-reports may be preferred over costly nutritional biomarkers (e.g. blood concentrations) that are not collected or measured in resource-constrained contexts or faced with implementation challenges [22]. Moreover, the validity of self-reported dietary assessment methods is well-documented [23, 24].

Classical statistics have developed mathematical models to explain inferential relationships between variables and outcomes such as type 2 diabetes, which are sometimes used to predict events although it is often inferential statistics underpinning these algorithms [25, 26]. Inferential statistics is constrained in the task of predictive modelling due to a number of reasons including that it struggles incorporating collinear factors and complex interactions. The pure prediction world is anti-parsimonious [27]; there will be a multitude of potential factors that combined together in complex non-linear ways can produce more accurate predictions for particular events. Real prediction is done using ML algorithms that are capable of detecting complex patterns and handling collinear factors, and are designed with the primary aim to predict future events [28]. Examining new factors as potential candidate predictors for type 2 diabetes or other clinical conditions using ML and large datasets formulate an extensive knowledge discovery process. Machine learning also has broadened our abilities to detect patterns between predictors and outcomes not previously possible [27]. With the increasing availability of big data, the scope to investigate a multitude of other possible predictors is now a reality. Together, large datasets and new analytical approaches with ML, have provided us with the opportunity to expand the knowledge base on other factors associated with type 2 diabetes. It is envisioned that identifying the best cohort of these predictors, many of which will have small effects, may be used to eventually build the best predictive tools with high predictive abilities and give clinicians and their patients the best certainty in risk prediction probabilities.

To date, no study has applied ML to explore nutritional markers of undiagnosed type 2 diabetes which could be used to improve its early diagnosis and understand its pathology beyond routinely-used risk factors. In this context, the present study used prediction models and ML, coupled with serial cross-sectional data from five consecutive cycles of the National Health and Nutrition Examination Survey (NHANES) (https://www.cdc.gov/nchs/nhanes/index.htm) over the years 2007–2016, with the aim of identifying nutritional markers that could predict undiagnosed type 2 diabetes together with routinely used non-modifiable, behavioural and socio-economic predictors. We also benchmarked the performance of these models against a national risk assessment method (i.e. ADA diabetes risk test) [16].

The rest of the manuscript is structured as follows: We first describe the database and study cohort followed by an account of the operationalisation of outcome variable. Thereafter, we detail the statistical analysis including data pre-processing, ML, and benchmarking steps. We then present results of univariate analyses followed by details of best-performing ML models derived by each algorithm and the elucidated nutritional markers. Results section is concluded with information on the findings from benchmarking and algorithmic performance comparison steps. Next, we discuss the strengths, limitations, novel aspects, and potential clinical implications of the study. Finally, conclusions of the study are presented.

## Materials and methods

### Data source and study sample

The NHANES is a series of biennial cross-sectional surveys conducted by the Centres for Disease Control and Prevention (CDC) [29]. This is a large database containing voluminous information from nationally-representative samples of non-institutionalised US civilians, which can be used for predictive analytic purposes.

**Fig 1:**
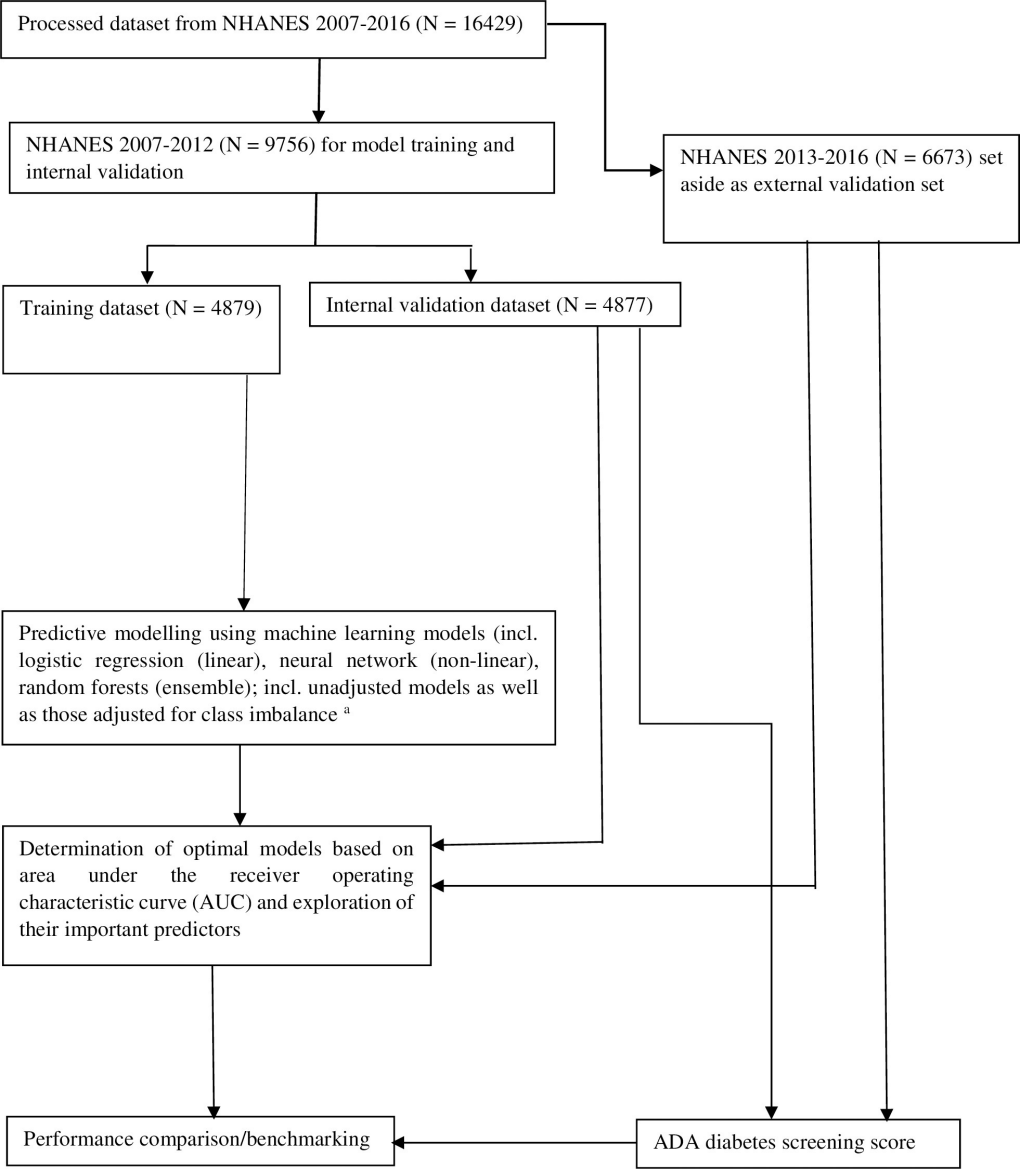
Flowchart depicting the analytic workflow adopted in the study. a-adjusted by resampling methods incl. oversampling, under-sampling, random oversampling (ROSE) and synthetic minority oversampling technique (SMOTE).

For this study, we pooled five consecutive cycles in order to maximise the number of adult participants with undiagnosed type 2 diabetes and to enable robust adjustment for potential confounders. Each survey cycle had been approved by the National Centre for Health Statistics Institutional Ethics Review Board and all adult participants had provided written informed consent. Additionally, Monash University Human Research Ethics Committee approved this study (#24888). The approach to participant selection is presented in **Fig 1**.

The resulting sample (n = 16429) included men and non-pregnant women ≥ 20 years of age with nutritional, behavioural, socio-economic and non-modifiable demographic data collected using pre-defined and uniform methods, from five consecutive data collection cycles of the NHANES spanning years 2007–2016. Design and methods of NHANES are well-documented (https://www.cdc.gov/nchs/nhanes/index.htm). In brief, dietary information was collected via two 24-hour dietary recall interviews; the first was an in-person visit in specially-designed Mobile Examination Centres (MECs) and the second was by telephone 3–10 days later. All dietary data were collected using similar methods in each survey cycle, enabling accurate total nutrient intake estimations and comparisons. Other health information was gathered by home-based interviews and via clinical examination in MECs. Although NHANES also collected serum biomarker data in MECs, these were not included, as we aimed to incorporate only easily collected, cost-effectively scalable nutritional and other clinical information frequently associated with dysglycaemia.

### Outcome variable

Undiagnosed type 2 diabetes among men and non-pregnant women ≥ 20 years of age was determined using all three diagnostic tests administered in NHANES: fasting plasma glucose [FPG], oral glucose tolerance test [OGTT], and haemoglobin A1c [HbA1c]. A participant was classified as having undiagnosed type 2 diabetes if they had a negative response to the question “Have you ever been told by a doctor that you have diabetes?” and a positive glycaemic response to the above diagnostic tests [HbA1c ≥ 48 mmol/mol (≥ 6.5%) or FPG ≥126 mg/dl or 2-hr post-OGTT glucose ≥ 200mg/dl] as per ADA criteria [30]. All diagnosed diabetes cases, defined by a positive response to the question above and a positive glycaemic response [HbA1c ≥ 48 mmol/mol (≥ 6.5%) or FPG ≥126 mg/dl or 2-hr post-OGTT glucose ≥ 200mg/dl] were removed. Since the aim was to elucidate markers of overt type 2 diabetes as opposed to normoglycaemia, individuals with prediabetes according to ADA criteria [HbA1c = 39–47 mmol/mol (5.7–6.4%) or FPG = 100–125 mg/dl or 2-hr post-OGTT glucose = 140–199 mg/dl] [31] were also removed. Normoglycaemia was defined as a negative response to the question above and a negative glycaemic response for all three diagnostic tests [HbA1c <39 mmol/mol (< 5.7%) and FPG < 100mg and OGTT < 140mg].

### Statistical analysis

The analytic workflow of this study was based on our previously published proof-of-study exploring predictors of prediabetes [32]. However, substantial modifications were made including analysing nutritional variables (omitted in the previous study) and excluding serum biomarkers in order to consider only those predictors which are simple, scalable and based on self-reported or easily measurable parameters. Another advancement was that, to be consistent with the cross-sectional design of NHANES, only undiagnosed type 2 diabetes was modelled in the present analysis whereas such a refinement was not applied to define the prediabetes cohort in the previous proof-of-concept study. We also used different benchmarking instruments in congruence with the two different conditions analysed in respective studies and we included much larger cohorts for training, testing, external validation and benchmarking. Finally, to identify all potential nutritional associations, we did not incorporate any statistical feature selection.

#### Data pre-processing

All analyses were performed using *R* statistical software [33]. Variables with ≥ 30% missing data were excluded, after which 139 variables that are potentially associated with undiagnosed type 2 diabetes (114 nutritional/dietary/food-intake associated; 13 other modifiable/health behaviour associated; 12 socio-economic/demographic) were included as independent variables, selected based on comprehensive literature surveys as summarised in **S1 Table**. The rationale for inclusion of behavioural and socio-economic variables was to enable robust adjustment of resulting multivariate models for these factors and to elucidate nutritional markers jointly with information that are routinely incorporated into type 2 diabetes screening.

Statistical feature selection was omitted as we aimed to identify all potential predictors of undiagnosed type 2 diabetes from the repertoire of 139 variables. The multiple imputation by chained equations (MICE) package [34] was used with default functions for imputing missing values; predictive mean matching, polytomous, and binary logistic regression for numeric, multi-level (> 2 levels) categorical and dichotomous categorical variables, respectively. Summary measures and variable distributions in the original and complete datasets were compared to evaluate goodness of fit.

The distribution of characteristics for individuals with undiagnosed type 2 diabetes and those with normoglycaemia within the entire cohort is outlined in **S2 Table**. NHANES 2013–2016 data were set aside as external validation sample to temporally validate constructed models. We performed random 50/50 split of the remaining NHANES 2007–2012 data to generate training samples (n = 4879) and internal validation samples (n = 4877).

#### Machine learning

We applied three ML algorithms, including logistic regression (LR) (linear), artificial neural network (ANN) (non-linear), and random forests (RF) (ensemble). To resolve the effect of class imbalance, resampling algorithms including minority class oversampling, Random OverSampling Examples (ROSE) [35], and Synthetic Minority Oversampling TEchnique (SMOTE) [36], were incorporated and trained in conjunction with each ML algorithm. Thus, a total of four models were built with: 1) original data, 2) oversampling, 3) ROSE, and 4) SMOTE per each ML algorithm. For ANN, parameter tuning and 5-fold cross-validation were conducted whereas default *R* package parameters and 10-fold cross validation were used for training the other two algorithms [37–39]. In detail, ANN settings were as follows: tuning grid composed of three weight decay parameters (0, 0.1, 0.01) and the size parameter was set from 1 to a maximum of 139 to be equivalent with the number of features. Bagging option was set to false and variable standardisation was performed via centering and scaling. The maximum number of iterations was 500. All other parameters were trained under default values.

**Fig 2:**
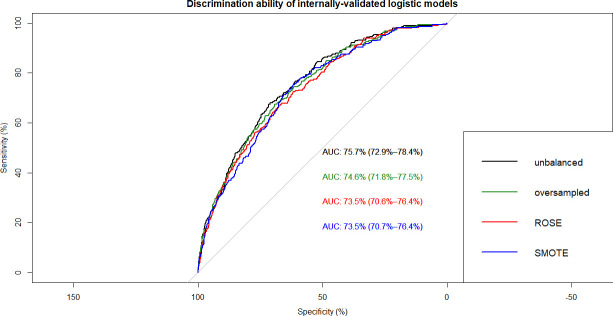
Overlapped ROC curves demonstrating predictive performance of logistic regression models on internal validation data. Using unbalanced, original training data and re-structured with oversampling, ROSE and SMOTE resampling.

**Fig 3:**
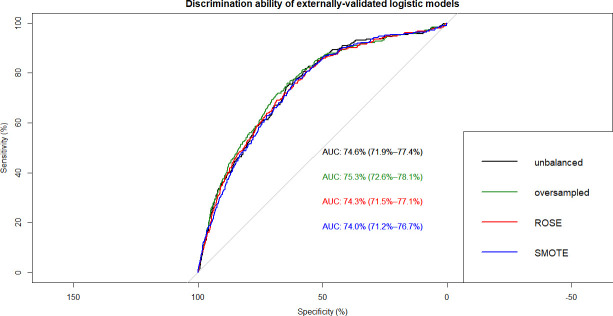
Overlapped ROC curves demonstrating predictive performance of logistic regression models on external validation data. Using unbalanced, original training data and re-structured with oversampling, ROSE and SMOTE resampling.

**Fig 4:**
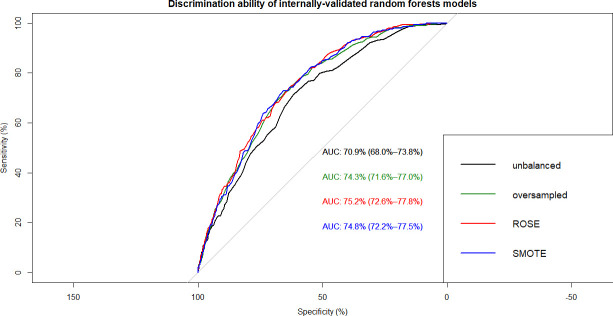
Overlapped ROC curves demonstrating predictive performance of random forests models on internal validation data. Using unbalanced, original training data and re-structured with oversampling, ROSE and SMOTE resampling.

**Fig 5:**
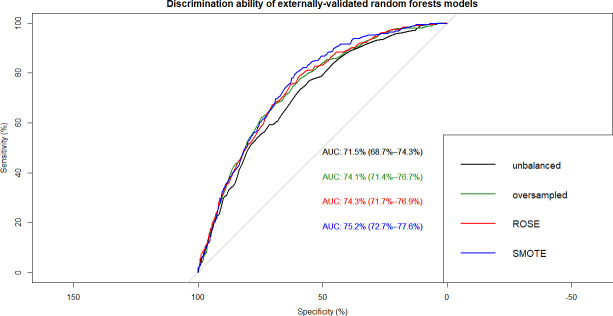
Overlapped ROC curves demonstrating predictive performance of random forests models on external validation data. Using unbalanced, original training data and re-structured with oversampling, ROSE and SMOTE resampling.

**Fig 6:**
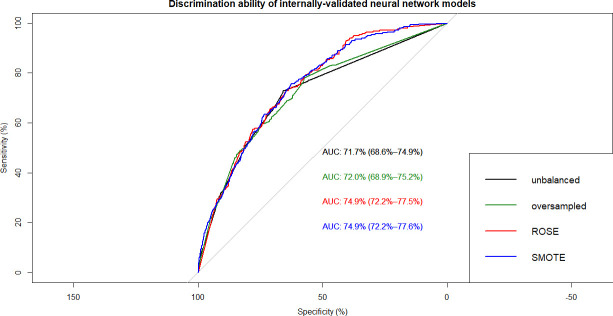
Overlapped ROC curves demonstrating predictive performance of artificial neural network models on internal validation data. Using unbalanced, original training data and re-structured with oversampling, ROSE and SMOTE resampling.

**Fig 7:**
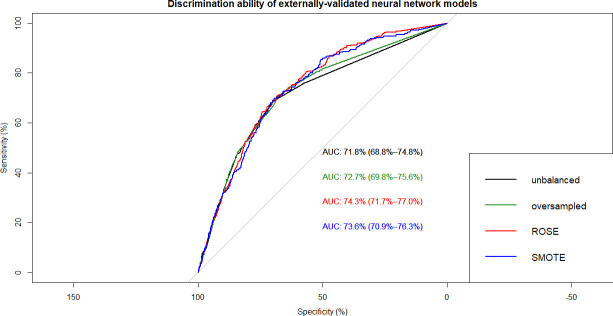
Overlapped ROC curves demonstrating predictive performance of artificial neural network models on external validation data. Using unbalanced, original training data and re-structured with oversampling, ROSE and SMOTE resampling.

**Fig 8:**
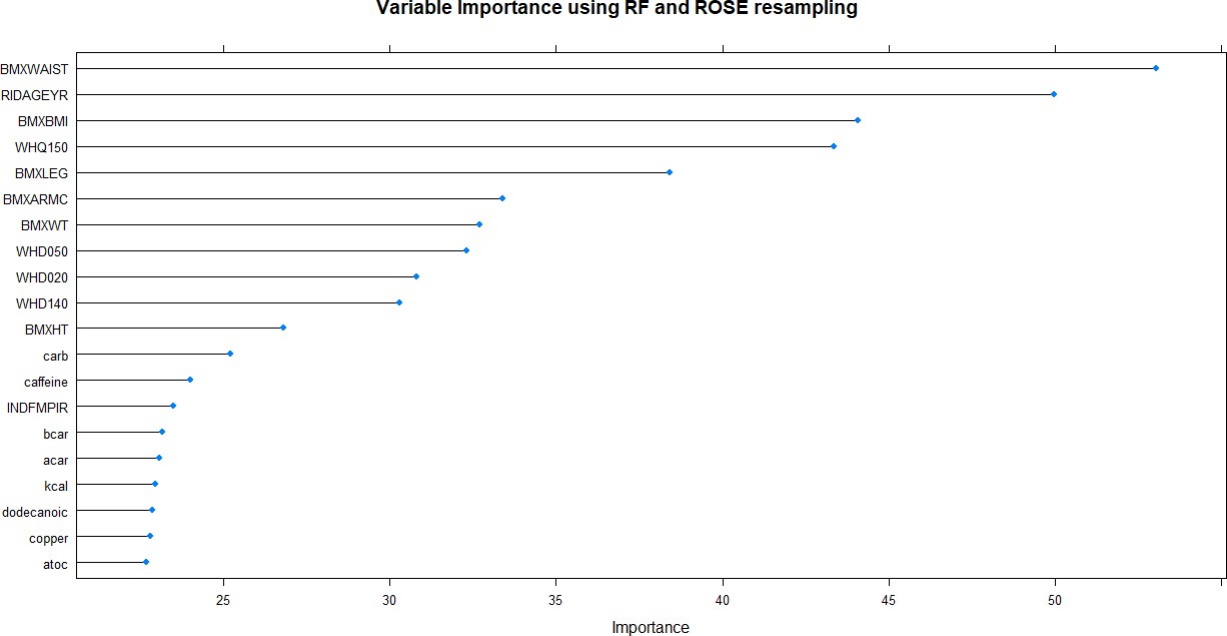
Variable importance plot of the best-performing random forest model produced by ROSE resampling. BMXWAIST = waist circumference; RIDAGEYR = age; BMXBMI = body mass index; WHQ150 = age when heaviest weight; BMXLEG = upper leg length; BMXARMC = arm circumference; BMXWT = weight; WHD050 = self-reported weight– 1 year ago; WHD020 = current self-reported weight; WHD140 = self-reported greatest weight; BMXHT = standing height; carb = carbohydrate; caffeine = caffeine; INDFMPIR = income-poverty ratio; bcar = beta carotene; acar = alpha carotene; kcal = energy; dodecanoic = SFA 12:0 (Dodecanoic); copper = copper; atoc = vitamin E alpha tocopherol.

**Fig 9:**
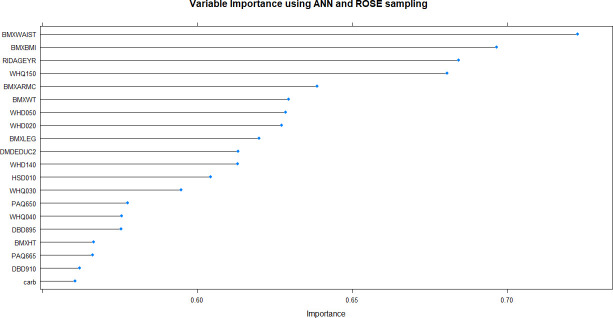
Variable importance plot of the best-performing artificial neural network model produced by ROSE resampling. BMXWAIST = waist circumference; BMXBMI = body mass index; RIDAGEYR = age; WHQ150 = age when heaviest weight; BMXARMC = arm circumference; BMXWT = weight; WHD050 = self-reported weight– 1 year ago; WHD020 = current self-reported weight; BMXLEG = upper leg length; DMDEDUC2 = education level; WHD140 = self-reported greatest weight; HSD010 = self-rated general health; WHQ030 = How do you consider your weight?; PAQ650 = vigorous recreational activities; WHQ040 = Like to weigh more, less, or same?; DBD895 = number of meals not home prepared; BMXHT = standing height; PAQ665 = moderate recreational activities; DBD910 = number of frozen meals/pizzas in past 30 days; carb = carbohydrate.

**Fig 10:**
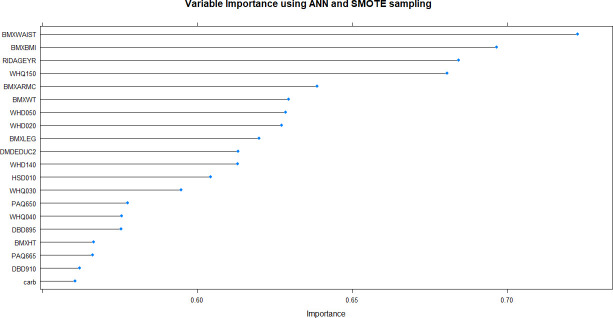
Variable importance plot of the best-performing artificial neural network models produced by SMOTE resampling. BMXWAIST = waist circumference; BMXBMI = body mass index; RIDAGEYR = age; WHQ150 = age when heaviest weight; BMXARMC = arm circumference; BMXWT = weight; WHD050 = self-reported weight– 1 year ago; WHD020 = current self-reported weight; BMXLEG = upper leg length; DMDEDUC2 = education level; WHD140 = self-reported greatest weight; HSD010 = self-rated general health; WHQ030 = How do you consider your weight?; PAQ650 = vigorous recreational activities; WHQ040 = Like to weigh more, less, or same?; DBD895 = number of meals not home prepared; BMXHT = standing height; PAQ665 = moderate recreational activities; DBD910 = number of frozen meals/pizzas in past 30 days; carb = carbohydrate.

This resulted in 12 ML models which were built on training data and tested on internal and external validation cohorts (**Figs 2–7**). Confusion matrix metrics such as sensitivity, specificity, and negative and positive predictive values as well as area under the receiver operating characteristic curve (AUROC) were used to assess the predictive performance of these models. Adjusted odds ratios (OR) indicated the relative impact of predictors in LR models with confidence intervals (CI) used to measure variability and significance. Predictors from the other two algorithms were identified by variable importance values, as calculated by default *R* software functions (**Figs 8–10**) [37–39].

Four best-performing models which produced highest AUROC per algorithm were identified: 1 each by LR and RF; 2 by ANN with the same AUROC. These models and the respective markers identified are summarised in **Tables 1 and 2.**

**Table 1:** Nutritional and other markers of undiagnosed type 2 diabetes identified by the best-performing logistic model (AUC = 75.7%).

GLM original ^a^	
(AUC_internal_ = 75.7%) ^b^	
(AUC_external_ = 74.6%) ^c^	
Marker	OR (95% CI)
***Nutrient-intake/Diet related***	
*Diet related* ^*d*^	
How healthy is the diet?	0.85 (0.72, 0.99)
Number of meals from fast food or pizza place	1.01 (1.00, 1.02)
*Anthropometry related* ^*e*^	
Weight	1.07 (1.01, 1.13)
Body mass index	1.06 (1.03, 1.09)
Waist circumference	1.06 (1.04, 1.09)
*Nutrient intake related* ^*e*^	
Total fat	1.14 (1.02, 1.27)
Beta-cryptoxanthin	0.99 (0.98, 1.00)
Folic acid	0.83 (0.69, 0.99)
Food folate	0.84 (0.74, 0.96)
Calcium	0.97 (0.94, 1.00)
Caffeine	0.998 (0.997, 1.000)
Vitamin B12	0.99 (0.98, 1.00)
***Other modifiable/behavioural*** ^***d***^	
Smoked at least 100 cigarettes in life? = yes (ref = no)	1.09 (1.00, 1.19)
***Socio-economic/Demographic*** ^***d***^	
Age	1.05 (1.03, 1.07)
Ethnicity = other (ref = White)	1.04 (1.01, 1.08)
Total number of people in the household	1.23 (1.01, 1.47)

a: logistic regression model on original, un-resampled data

b: area under receiver operating characteristic curve on the internal validation data

c: area under receiver operating characteristic curve on the external validation data

d: self-reported

e: measured via two 24-hour dietary recalls.

AUC = area under receiver operating characteristic curve.

**Table 2:** Nutritional and other markers of undiagnosed type 2 diabetes identified by best-performing ANN and RF models.

RF ROSE ^a^		ANN ROSE ^b^		ANN SMOTE ^c^	
(AUC_internal_ = 75.2%) ^d^		(AUC_internal_ = 74.9%) ^d^		(AUC_internal_ = 74.9%) ^d^	
(AUC_external_ = 74.3%) ^e^		(AUC_external_ = 74.3%) ^e^		(AUC_external_ = 73.6%) ^e^	
Marker	Importance ^f^	Marker	Importance ^g^	Marker	Importance ^g^
***Nutritional***		***Nutritional***		***Nutritional***	
*Anthropometry-related*		*Diet/food-intake related*		*Diet/food-intake related*	
Waist circumference	53.03	Number of meals not home prepared	0.5755	Number of meals not home prepared	0.5755
		Number of frozen meals/pizzas in past 30 days	0.5620	Number of frozen meals/pizzas in past 30 days	0.5620
Body mass index	44.07	*Anthropometry-related*		*Anthropometry-related*	
Age when heaviest weight	43.35	Waist circumference	0.7228	Waist circumference	0.7228
Upper leg length	38.42	Body mass index	0.6967	Body mass index	0.6967
Arm circumference	33.41	Age when heaviest weight	0.6806	Age when heaviest weight	0.6806
Weight	32.70	Arm circumference	0.6386	Arm circumference	0.6386
Self-reported weight—1 year ago	32.32	Weight	0.6295	Weight	0.6295
Current self-reported weight	30.81	Self-reported weight—1 year ago	0.6285	Self-reported weight—1 year ago	0.6285
Self-reported greatest weight	30.31	Current self-reported weight	0.6272	Current self-reported weight	0.6272
Standing height	26.82	Upper leg length	0.6199	Upper leg length	0.6199
*Nutrient intake-related*		Self-reported greatest weight	0.6130	Self-reported greatest weight	0.6130
Carbohydrate	25.21	How do you consider your weight?	0.5948	How do you consider your weight	0.5948
Caffeine	24.01	Like to weigh more, less or same?	0.5755	Like to weigh more, less or same	0.5755
		Standing height	0.5665	Standing height	0.5665
Beta-carotene	23.18	*Nutrient intake related*		*Nutrient intake-related*	
Alpha-carotene	23.09	Carbohydrate	0.5606	Carbohydrate	0.5606
Energy	22.96	***Other modifiable/health behaviour associated***		***Other modifiable/health behaviour associated***	
SFA 12:0 (Dodecanoic)	22.88	Self-rated general health	0.6043	Self-rated general health	0.6043
Copper	22.82	Vigorous recreational activities	0.5776	Vigorous recreational activities	0.5776
Vitamin E as alpha-tocopherol	22.70	Moderate recreational activities	0.5662	Moderate recreational activities	0.5662
***Socio-economic/Demographic***		***Socio-economic/Demographic***		***Socio-economic/Demographic***	
Age	49.96	Age	0.6843	Age	0.6843
Income-poverty ratio	23.51	Education level	0.6132	Education level	0.6132

a = random forest model on train data restructured by ROSE sampling

b = artificial neural network model on training data restructured by ROSE sampling algorithm

c = artificial neural network model on training data restructured by SMOTE sampling algorithm

d = area under receiver operating characteristic curve on the internal validation data

e = area under receiver operating characteristic curve on the external validation data

f = by default, mean decrease in prediction accuracy after a variable is permuted

g = default method uses combinations of the absolute values of the weights.

ANN = artificial neural network; AUC = area under receiver operating characteristic curve; RF = random forest; ROSE = random oversampling examples; SFA = saturated fatty acid; SMOTE = synthetic minority oversampling technique.

#### Benchmarking

**Fig 11:**
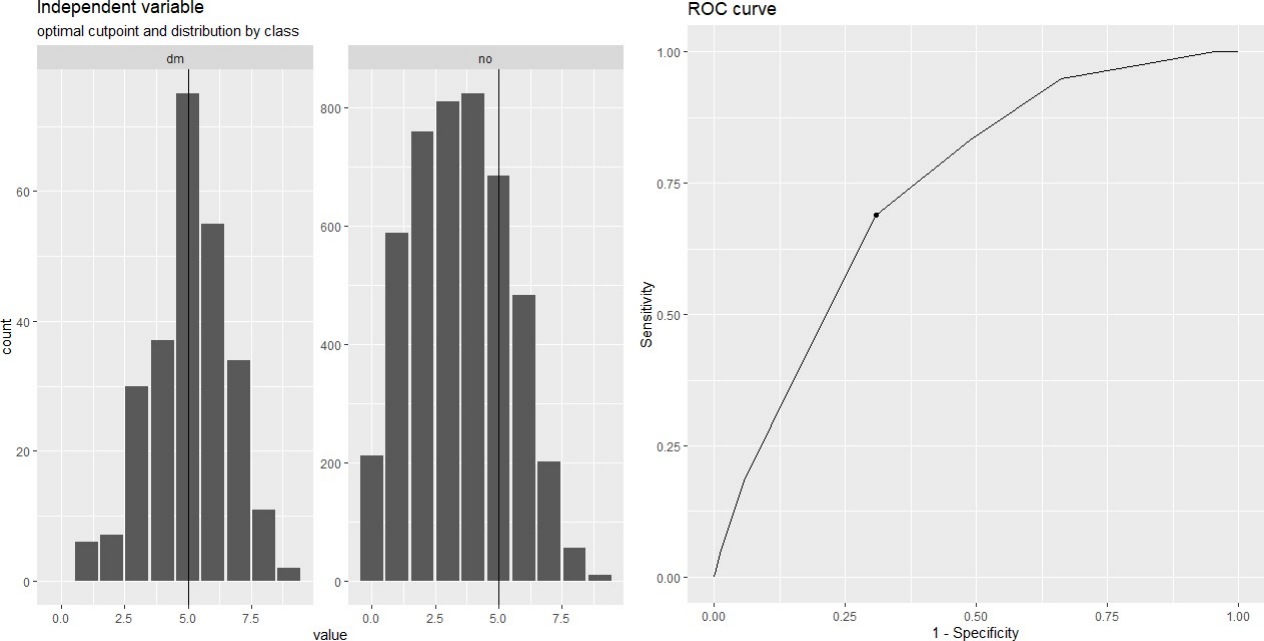
Benchmarking with the ADA diabetes risk test. Comparison of predictive performance of ADA diabetes risk test on internal validation data (AUC = 0.737028) and the best-performing predictive model on internal validation data (AUC = 0.7566544), as per DeLong test for comparing two ROC curves, was non-significant (p = 0.3201) indicating performances on a par with each other. Comparison of predictive performance of ADA diabetes risk test on external validation data (AUC = 0.7401352) and the best-performing predictive model on external validation data (AUC = 0.7464), as per DeLong test for comparing two ROC curves, was also non-significant (p = 0.0643).

We compared the predictive performance of best-performing models on internal and external validation data against the performance of an appropriate benchmark (i.e. ADA diabetes risk test) [16]. As there were discrepancies between ADA risk test criteria and NHANES variables, the instrument needed to be adapted suitably in order to estimate its parameter scores. Thus, we modified the ADA diabetes risk test enabling to use it on the NHANES cohorts as shown in **Table 3.** The ADA diabetes risk test collects information on 7 risk factors: age, gender, previous gestational diabetes (if female), first degree relative with diabetes, hypertension, physical activity and BMI. Total risk score is in the range of 0–10 whereas the cut-point indicating high risk of diabetes is 5. Therefore, we categorised participants with a total risk score ≥5 as at high risk of diabetes and those with < 5, not at high risk. We performed this classification on both internal and external validation cohorts. Next, AUROC achieved by the ADA risk test on these cohorts were calculated and compared to the corresponding AUROC estimates of best-performing models using DeLong test [40] (**Table 4; Fig 11**). Finally, algorithmic performances across all models were compared using Hanley and McNeil test for comparing ROC curves [41] (**S3 Table**).

**Table 3:** Creation of variables analogous to those in the American Diabetes Association (ADA) diabetes risk test using National Health and Nutrition Examination Survey (NHANES) data.

Variable	Information used/modified from NHANES	Score*
Age	Age in years at screening was categorised with following cut-points to ascribe scores	
	<40	0
	40–49	1
	50–59	2
	≥60	3
Gender	Self-reported gender	
	Female	0
	Male	1
Previous gestational diabetes (if female)	Self-reported history of gestational diabetes	
	No	0
	Yes	1
1^st^ degree relative with diabetes	NHANES questionnaire collects information on familial diabetes, but not on 1^st^ degree relatives with diabetes per se, so the self-reported family history of diabetes was used as a proxy variable.	
	No	0
	Yes	1
Hypertension (self-reported history of hypertension, prescribed antihypertensive medication, and/or BP ≥140/90)	NHANES provides information on all 3 criteria; self-reported history of hypertension (“Ever told you had high blood pressure?”), prescribed antihypertensive medication (“Taking prescription for hypertension?”) and/or BP ≥140/90 (objectively measured and averaged over 3 or 4 measurements of SBP and DBP).	
	Absence of all 3 criteria	0
	Presence of history of self-reported hypertension or prescribed antihypertensive medication, or BP ≥140/90	1
Physically active (self-reported)	Derived a binary variable by checking if any of the following activities were done in 5 or more days of a typical week: vigorous or moderate work, recreational work, walk or bicycle	
	Yes	0
	No	1
BMI, kg/m^2^	Available in NHANES. Objectively measured.	
	<25	0
	25 to <30	1
	30 to <40	2
	≥40	3

* Cumulative scores ≥5 should be formally screened for diabetes, per ADA guidelines, which was chosen as the cut-point for classifying individuals.

ADA = American Diabetes Association; BMI = body mass index; BP = blood pressure; NHANES = National Health and Nutrition Examination Survey.

**Table 4:** Performance comparison of the ADA diabetes risk test versus the best-performing model on NHANES data.

Benchmarking with the best-performing ML model ^a^				
Criterion	ADA diabetes risk test		Best-performing ML model ^a^	
	Performance upon the internal validation dataset ^b^ (N = 4877)	Performance upon the external validation dataset ^c^ (N = 6673)	Performance upon the internal validation dataset ^b^ (N = 4877)	Performance upon the external validation dataset ^c^ (N = 6673)
AUROC	0.737028	0.7401352	0.7566544	0.7464869
Sensitivity	0.688716	0.7639015	0.6810036	0.7745098
Specificity	0.690244	0.6109271	0.7105263	0.6148893
Accuracy	0.690164	0.6319564	0.7088374	0.6222089
PPV	0.147892	0.1092312	0.1249178	0.0881368
NPV	0.912503	0.9219408	0.9734803	0.9826807

a = This was a logistic regression model on original, unbalanced training data without any resampling

b = A randomly partitioned sample from NHANES 2007–2012

c = from NHANES 2013–2016.

ADA = American Diabetes Association; AUROC = area under the receiver operating characteristic curve; ML = machine learning; PPV = positive predictive value; NPV = negative predictive value.

## Results

Undiagnosed type 2 diabetes defined by all three diagnostic tests (FPG, OGTT, and HbA1c) was prevalent in 5.6% (n = 16429) of the sample. The age distribution of the sample ranged from 20–80 years, with a mean ± SD of 47 ± 17.24 years.

As per univariate analysis of categorical variables, the normoglycaemic cohort (n = 15564) included a significantly greater number of Non-Hispanic White, self-reported citizens as well as participants who: self-reported vigorous or moderate work activity; walking or bicycling; vigorous or moderate recreational activities; considered themselves underweight or about the right weight; liked to weigh more or stay about the same; self-reportedly used ordinary salt and community supply as their tap water source; consumed shellfish during the past 30 days; and took dietary supplements. Univariate analysis of numeric variables revealed that normoglycaemic individuals had significantly higher education, income-poverty ratio, self-rated general health, self-reported dietary health, household food security category, adult food security category, monthly family income, family monthly income-poverty level index, family monthly income-poverty level category, standing height, upper leg length, upper arm length, current self-reported height, total plain water drank the previous day and total tap water drank the previous day. A number of dietary constituents were also significantly higher in the normoglycaemic group, namely, dietary protein, total monounsaturated fatty acids (MFA), added alpha-tocopherol (vitamin E), lutein + zeaxanthin, thiamin (vitamin B1), total folate, folic acid, food folate, folate, dietary folate equivalents (DFE), vitamin B12, vitamin D (D2 + D3), phosphorus, sodium, caffeine, dietary water content/moisture and MFA 18:1 (octadecenoic) levels (**S2 Table**).

As per univariate analysis of categorical variables, males, those who received household (HH) emergency food, smoked at least 100 cigarettes in life, considered themselves overweight, liked to weigh less, did not use/add salt products at the table, and did not drink tap water were significantly higher in those with undiagnosed type 2 diabetes (n = 865). Univariate analysis of numeric variables revealed that the undiagnosed type 2 diabetes group were older in age, had significantly greater BMI, arm- and waist- circumference, weight (including self-reported current weight, weight 1-year ago, self-reported greatest weight), age when heaviest weight, and minutes of sedentary activity, reported significantly greater amount of money spent on eating out, past 30-day milk product consumption, number of meals not home prepared, number of meals from fast food or pizza places, number of ready-to-eat foods in the past 30 days, and the number of frozen meals/pizzas in the past 30 days. Moreover, among those with undiagnosed type 2 diabetes, a greater number of individuals self-reported that HH food did not last or HH could not afford balanced meals while higher dietary intakes of energy, carbohydrate, total fat, total saturated fatty acids (SFA), SFA 10:0 (decanoic) (gm), SFA 12:0 (dodecanoic), and SFA 16:0 (hexadecanoic) levels were also observed among them (**S2 Table**).

The best-performing LR model was based on original, un-resampled training data and produced an AUROC of 75.7% and 74.6% on internal- and external validation data, respectively (**Fig 2**). As shown in **Table 1**, the model identified 16 significant predictors of undiagnosed type 2 diabetes encompassing nutritional, behavioural, and socio-economical markers. Among nutritional markers, 2 diet-related (how healthy is the diet, number of meals from fast food or pizza place), 3 anthropometric (weight, BMI, waist circumference), and 7 nutrient intake-related (total fat, beta-cryptoxanthin, folic acid, food folate, calcium, caffeine, vitamin B12) predictors could be identified. Smoking at least 100 cigarettes in life was also a significant behavioural predictor of undiagnosed type 2 diabetes. The 3 socio-economic/demographic markers were the age, ethnicity, and the total number of people in the household.

Using non-linear or ensemble ML algorithms, three best-performing models were generated. Of random forests models, RF with ROSE resampling was the best-performing model with AUROC of 75.2% on internal validation data. Two best-performing models were produced by ANN, namely, ANN with ROSE resampling and ANN with SMOTE resampling, which had approximately the same AUROC of 74.9% on internal validation data (**Table 2**). These two ANN models had following specifications: logistic output functions; feed-forward, 5-fold cross-validated neural networks; automatically standardised input variables with tuned parameters. Five neural networks were trained and the mean values of resulting predictions comprised the model output. The best-performing RF model was built using 10-fold cross-validation and also consisted of automatically standardised variables with default *R* package functions and parameters.

Out of the 39 markers of undiagnosed type 2 diabetes, 30 were nutritional. Moreover, 11 of the 39 markers were uniquely identified by the LR model, while the remaining 28 markers emerged from one or more of the three best-performing non-linear/ensemble models. Of these 28 markers, 12 were common across all three models whilst eight were unique to each of the RF and ANN models. Notably, of the 16 significant predictors identified by the best-performing LR model, 11 were exclusive. Four markers including age, waist circumference, BMI, and weight were common to all four models, while one marker, dietary caffeine intake, was elucidated by both logistic and RF models.

Internal (n = 4877) and external (n = 6673) validation data acquired AUROC estimates of 73.7% and 74.0%, respectively, for the ADA diabetes risk test. When ROC curves were compared using the DeLong test, the AUROC estimates from the four best-performing models did not differ significantly from the corresponding ADA diabetes risk test estimates (*p*>0.05), with AUROC differences ranging from 0.2–2.8% and 0.0–2.5% on internal and external validation data, respectively. Performance of the ADA diabetes risk test using NHANES data, compared to performance of the best-performing classifier with the highest AUROC is outlined in **Table 4**.

As depicted in **S3 Table**, comparison of models derived using each algorithm indicated that none were significantly different from each other (p>0.05) within both internal- and external- validation cohorts.

## Discussion

In summary, this analysis revealed several nutritional markers of undiagnosed type 2 diabetes comprising diet-related, anthropometric, and nutrient-based variables that can be used in concert with regularly obtainable behavioural and socio-economic information to optimise current prediction models. Despite being readily available or easily collected, most of the nutritional predictors revealed by this analysis, are not presently used in type 2 diabetes risk assessment instruments and procedures.

As this is the first study to apply ML to ascertain nutritional markers of undiagnosed type 2 diabetes, our findings provide important groundwork for precision nutrition approaches in diabetes prevention which are currently lacking [42], through identification of a diverse set of simple, cost-effective nutritional markers. Whilst it has been argued that policy-level management of obesogenic environments would be more prudent, precision nutrition approaches are critical for understanding the impact of nutrition on individual risk of type 2 diabetes and providing stratified care [43]. Notably, our emphasis in this analysis was on modifiable markers, including an array of nutritional markers which stands different to the status quo as the current approach to type 2 diabetes risk assessment is leveraged on non-modifiable markers. As shown by our findings, the broad array of nutritional and behavioural markers of undiagnosed type 2 diabetes may collectively augment contemporary risk prediction efforts, although most of their individual impact may be smaller. In addition, these markers may be modified to optimise glycaemic status, following deeper understanding of the aetiological pathways by which they are associated with type 2 diabetes.

A recent meta-analysis of ML models for type 2 diabetes prediction in community settings revealed that external validation was conducted by none suggesting poor generalisability [44]. However, we conducted temporal external validation in the present study thereby achieving higher generalisability to the US population. Applicability beyond the US is likely to be limited by the contextual nature of certain predictors as well as geographic, ethnic, and other variations among target populations. Moreover, nutrients like beta-cryptoxanthin as markers pose challenges to translation and implementation strategies, whereas food-based prediction may facilitate seamless translation. Since food-based dietary guidelines are easier to upscale than traditional nutrient-based guidelines [45, 46], food-based predictive modelling is a necessary consideration for future research and could yield directly translatable findings in terms of type 2 diabetes management and dietary recommendations. A recent study revealed that food-based dietary guidelines (FBDG) currently set up in 90 countries, contained universal as well as variable recommendations. It also underscored that socio-cultural aspects should be considered in the development of country-specific FBDG [47]. While these FBDG are non-specific and targeted to the general population, findings from food-based predictive modelling studies on specific cohorts such as people with type 2 diabetes might offer opportunities to implement disease-specific FBDG. Such advancements would create pathways to provide more tailored, disease-specific nutritional care. Although residual confounding cannot be ruled out, it should be noted that we adjusted our models for multiple variables to minimise the impact of confounding while only undiagnosed type 2 diabetes was modelled to acquiesce with the cross-sectional design of NHANES. Also, ML algorithms are tolerant of complexities inherent in multidimensional data, allowing for multivariate modelling with large datasets and numerous variables to gain meaningful insights [48].

Although all three algorithms performed in a comparable manner, disparities in the nutritional markers identified by them are notable. This underscores the importance of applying an array of algorithms instead of a single learner to get comprehensive insights. Only 5 predictors were identified by both linear and non-linear/ensemble algorithms, which can be explained by differences in underlying prediction dynamics. LR models reveal linear associations whereas ensembles and non-linear learners unearth more complex, non-linear associations [49]. Neural networks modelled after the architecture of the human nervous system, are non-linear organisations consisting of input-, hidden-, and output- layers capable of handling large quantities of data and yielding novel patterns, interactions, and features [50]. Random forests algorithm is structured as an ensemble of trees in which each tree represents a vector of random variables. It offers high computational efficiency and interpretable outputs via variable importance estimates [51, 52]. Therefore, structural and functional differences of the applied algorithms would have contributed to the variations in the predictors identified by each. Most of our findings are supported by contemporary studies. Anthropometric markers were identified by all algorithms, offering solid support for their high predictive value in type 2 diabetes prediction. Findings indicate that a much broader set of anthropometric markers beyond BMI may be useful for improving the existing type 2 diabetes prediction paradigm.

Noteworthily, ultra-processed food consumption has been found an emerging risk factor of type 1- [53], type 2- [54] as well as gestational [55] diabetes by recent studies which is supported by findings in the present study as well. Similarly, caffeine intake has been associated with reduced risk of type 2 diabetes in previous studies as well [56, 57]. Overall, it seems that all models are equivalent to the ADA diabetes risk test, which is possibly because of the disproportionately high importance of age and BMI. In addition, the mediating effects of BMI and other anthropometric variables may have contributed to the lack of importance of some nutritional factors. Since the primary goal in the current analysis was prediction rather than aetiology, studies are needed to further explore the aetiological pathways. As a whole, the markers of undiagnosed type 2 diabetes found in the present study are simple and scalable including a number of self-reported predictors. Higher evidence on these associations should therefore be gathered via designs such as longitudinal, follow-up studies or pragmatic trials to be incorporated into future nutritional guidelines for prevention of type 2 diabetes.

A limitation in NHANES data is that there is no direct information on type 1- or type 2- or other diabetes phenotypes and previous studies used different strategies for defining diabetes phenotypes [58–61]. Consequently, there may have been a negligible number of adults with undiagnosed type 1 diabetes in our samples. Moreover, the prevalence of undiagnosed type 2 diabetes in this study may have increased with the use of all 3 glycaemic tests for its definition whereas only 1 or 2 glycaemic tests were used in previous studies and with comparative differences in other criteria adopted in its operationalisation [58–61].

This study demonstrated that the proof-of-concept ML workflow previously proposed by us [32] is viable, when applied to different research contexts with appropriate modifications, indicating a high degree of generalisability and adaptability. As the obstacles to implementing ML interventions in healthcare are widespread and systemic [62], those requiring only customarily compiled health information would offer more realistic solutions. Therefore, deliberate exclusion of serum biomarkers from the present analysis has produced prototypical models containing information that can be broadly and easily integrated into the nutritional management of people with type 2 diabetes. While predictors such as serum biomarkers and -omics data form an important and sought-after part of precision nutrition [63], the current analysis showed that the exhaustive usage of regularly available information may help identify novel nutritional markers of type 2 diabetes and enhance prediction hence should not be overlooked.

## Conclusion

In conclusion, we report a smorgasbord of novel and classic nutritional markers that could be used concomitantly with known behavioural and non-modifiable markers to optimise the prediction of undetected type 2 diabetes in adults. Our findings may have practical implications as a step towards personalised clinical nutrition, such as risk-stratified nutritional recommendations and early preventive strategies aimed at high risk individuals as well as in the nutritional management of people with type 2 diabetes.

## Supporting information

S1 Table List of nutritional and other variables from NHANES 2007–2016 included as independent variables in machine learning. (DOCX)

S2 Table Distribution of features in the entire cohort (N = 16429) between undiagnosed T2D and non-T2D (normoglycaemic) individuals. (DOCX)

S3 Table Comparison of algorithmic performance across internally- and externally-validated models. (DOCX)
