# Supplementary material for: Nutritional markers of undiagnosed type 2 diabetes in adults: Findings of a machine learning analysis with external validation and benchmarking
Source: PLoS One. 2021 May 5;16(5):e0250832. doi: 10.1371/journal.pone.0250832 (PMC8099133; doi:10.1371/journal.pone.0250832)
Supplement: S1 Table — (DOCX) [file pone.0250832.s001.docx]

**S1 Table: List of nutritional and other variables from NHANES 2007-2016 included as independent variables in machine learning.**

|  | **Variable** | **Explanation and/or its operationalisation, construction and coding** | **Association with dysglycaemia/rationale** |
| --- | --- | --- | --- |
| **Nutritional/Dietary/Food-intake associated** | | |  |
| 1 | HH Emergency food received | Question from the NHANES questionnaire: “In the last 12 months, did {you/you or any member of your household} ever get emergency food from a church, a food pantry, or a food bank, or eat in a soup kitchen?” Responses were recoded as 1 = no, 2 = yes | A case-control study revealed food insecurity is an important risk factor for type 2 diabetes risk (Najibi et al., 2019). A study done on the NHANES 1999-2002 data found that food insecurity was a risk factor for diabetes. Authors suggested that increased consumption of inexpensive, high-caloric food alternatives, among adults with food insecurity could play a part in the observed relationship (Seligman et al, 2007). This variable was added as a proxy indicator of access to nutritious food. |
| 2 | HH FS benefit: ever received | Question from the NHANES questionnaire: “The next questions are about the Food Stamp Program. Food stamps are usually provided on an electronic debit card {or EBT card} {called the {{STATE NAME FOR EBT CARD}} card in {{STATE}}}. Have {you/you or anyone in your household} ever received Food Stamp benefits?” Responses were recoded as 1 = no, 2 = yes. | See no:1 above |
| 3 | How do you consider your weight | Question from the NHANES questionnaire: {Do you/Does SP} consider {your/his/her}self now to be . . . [If {you are/she is} currently pregnant, what did {you/she} consider {your/her}self to be before {you were/she was} pregnant?]” Recoded as 1 = underweight, 2 = about the right weight, 3 = overweight. | Mogre et al (2014) found a substantial discordance between BMI-measured and self-perceived weight status among type 2 diabetes patients. Factors that were associated with underestimation of weight status were; overweight/obese, not married and never tried to lose weight. They recommended diabetes patients should be provided with information about weight guidelines. |
| 4 | Like to weigh more, less or same | Question from the NHANES questionnaire: “Would {you/SP} like to weigh . . .” Recoded as 1 = more, 2 = stay about the same, 3 = less. | In a study by Green et al (2007), type 2 diabetes and high-risk respondents reported attitudes and knowledge conducive to good health, but the majority of respondents did not translate these positive traits into healthy behavior with respect to diet, exercise and weight loss. We hypothesized an association between self-perceived weight and type 2 diabetes risk. |
| 5 | Tried to lose weight in past year? | Question from the NHANES questionnaire: “During the past 12 months, {have you/has SP} tried to lose weight?” Recoded as 1 = no, 2 = yes. | A randomized controlled trial revealed that weight loss was the dominant predictor of reduced diabetes incidence. For every kilogram of weight loss, there was a 16% reduction in risk, adjusted for changes in diet and activity. Lower percent of calories from fat and increased physical activity predicted weight loss. Increased physical activity was important to help sustain weight loss. Among 495 participants not meeting the weight loss goal at year 1, those who achieved the physical activity goal had 44% lower diabetes incidence. It concluded that interventions to reduce diabetes risk should primarily target weight reduction (Hamman et al., 2006). |
| 6 | Type of table salt used | Question from the NHANES questionnaire: “What type of salt {do you/does SP} usually add to {your/his/her/SP's} food at the table? Would you say…” Recoded as 1 = Ordinary salt [includes regular iodized salt, sea salt and seasoning salts made with regular salt], 2 = Lite salt/ Salt substitute, 3 = Doesn't use or add salt products at the table | possible relationship between additional adding of salt to prepared meals and an increased risk of type 2 diabetes was revealed in a case-control study (Radzeviciene & Ostrauskas, 2017). |
| 7 | On special diet? | Question from the NHANES questionnaire: “Are you currently on any kind of diet, either to lose weight or for some other health-related reason?” Recoded as 1 = no, 2 = yes. | Relationship between special diet and diabetes risk has been underscored by a number of studies, for example, with low-carb and ketogenic diet (Bolla et al., 2019). Mediterranean diet (Salas-Salvadó et al., 2015) and Nordic diet (Kanerva et al., 2014). |
| 8 | Compare food consumed yesterday to usual | Question from the NHANES questionnaire: “Was the amount of food that {you/NAME} ate yesterday much more than usual, usual, or much less than usual?” Coded as 1 = Much more than usual, 2 = Usual, 3 = Much less than usual. | A prospective cohort study revealed that food intake pattern was strongly related to plasminogen activator inhibitor-1 (PAI-1) and fibrinogen and independently predicted type 2 diabetes (Liese et al., 2009). Another prospective cohort study reported that breakfast omission was associated with an increased risk of T2D in men even after adjustment for BMI whilst a direct association between snacking between meals and T2D risk was mediated by BMI (Mekary et al., 2012). |
| 9 | Tap water source | Question from the NHANES questionnaire: “When you drink tap water, what is the main source of the tap water? Is the city water supply (community water supply); a well or rain cistern; a spring; or something else?” Coded as 1 = Community supply, 2 = Well or rain cistern, 3 = Spring, 4 = Don't drink tap water. | A prospective cohort study revealed that self-reported water intake was inversely and independently associated with the risk of developing hyperglycemia (Roussel et al., 2011). Another population-based case control study found that tap water pH 6.2–6.9 was associated with a fourfold higher risk of type 1 diabetes compared with pH ≥7.7. This result was similar after exclusion of individuals with the highly protective HLA-DQB1*0602 allele, but adjustment for maternal education, urban/rural residence, sex, and age tended to strengthen the estimated association. Higher tap water concentration of zinc was associated with lower risk of type 1 diabetes after adjustment for pH and other possible confounders, but the overall association was strictly not significant. It concluded that the quality of drinking water influences the risk of type 1 diabetes. The possible mechanisms by which water acidity or mineral content may be involved in the etiology of type 1 diabetes remain unknown, but the mechanisms are most likely indirect and may involve an influence on survival of microorganisms in the water (Stene et al., 2002). We hypothesized a potential association with type 2 diabetes in this study. |
| 10 | Shellfish eaten during past 30 days | Question from the NHANES questionnaire: “Please look at this list of shellfish. During the past 30 days did you eat any types of shellfish listed on this card? Include any foods that had shellfish in them such as sandwiches, soups, or salads.” Coded as 1 = no, 2 = yes. | A population-based prospective cohort (European Prospective Investigation of Cancer [EPIC]-Norfolk) study, found that the total, white, and oily fish consumption may be beneficial for reducing risk of diabetes, reinforcing the public health message to consume fish regularly. Greater shellfish intake seems to be associated with an increased risk of diabetes, warranting further investigation into cooking methods and mechanisms (Patel et al., 2009). A prospective population-based cohort study of Norwegian women found that lean fish consumption of 75–100 g/d had a beneficial effect on T2DM. It was inconclusive whether lean fish in itself has a protective effect on T2DM or that lean fish consumers have a protective life-style that was not considered in this study. Unfavorable effects of fatty fish consumption or use of cod liver oil supplements on T2DM were not observed (Rylander et al., 2014). A prospective cohort study of Swedish men revealed no overall association between total fish consumption and type 2 diabetes. The results indicated that dietary contaminants in fish may influence the relationship. Fried fish and shellfish consumption were associated with higher type 2 diabetes incidence. These findings suggest that more specific advice on fish species sub-types (varying in contamination) and preparation methods may be warranted (Wallin et al., 2017). |
| 11 | Fish eaten during past 30 days | Question from the NHANES questionnaire: “Please look at this list of fish. During the past 30 days did you eat any types of fish listed on this card? Include any foods that had fish in them such as sandwiches, soups, or salads.” Coded as 1 = no, 2 = yes. | See no:10 above. |
| 12 | Any dietary supplements taken? | Question from the NHANES questionnaire: “The next questions are about {your/SP's} use of dietary supplements and medications during the past month. {Have you/Has SP} used or taken any vitamins, minerals or other dietary supplements in the past month? Include those products prescribed by a health professional such as a doctor or dentist, and those that do not require a prescription. This card lists some examples of different types of dietary supplements.” Coded as 1 = no, 2 = yes. | A review found that nutritional supplements are commonly used as an alternative or as an addition to their current diet to prevent T2DM (Yeung et al., 2018). |
| 13 | Any antacids taken? | Based on the questionnaire “Any Antacids Taken?” Coded as 1 = no, 2 = yes. | A prospective cohort study revealed that the presence and the extent of gastric atrophy, but not H. pylori infection, are associated with incident diabetes (Yu et al., 2017). |
| 14 | Money spent at supermarket/grocery store | Question from the NHANES questionnaire: “The next questions are about how much money {your family spends/you spend} on food. First I'll ask you about money spent at supermarkets or grocery stores. Then we will talk about money spent at other types of stores. During the past 30 days, how much money {did your family/did you} spend at supermarkets or grocery stores? Please include purchases made with food stamps.” | A prospective cohort revealed that multiple food groups collectively influence type 2 diabetes risk beyond that of the individual food groups themselves (Nettleton et al., 2008). An analysis of NHANES 1999-2006 found that higher prices of healthy foods were associated with increased blood sugar among people with type 2 diabetes, which was especially pronounced among low-income people with type 2 diabetes (Anekwe &Rahkovsky, 2018). |
| 15 | Money spent on nonfood items | Based on the questionnaire “About how much money was spent on nonfood items?” Coded as dollar amount of money spent in 30 days. | Food security and purchasing power are associated with the prevention and management of diabetes (Gucciardi et al., 2014). |
| 16 | Money spent on food at other stores | Question from the NHANES questionnaire: “About how much money {did your family/did you} spend on food at these types of stores? (Please do not include any stores you have already told me about.)” Coded as dollar amount of money spent in 30 days. | A large multi-ethnic cohort study reported higher neighborhood prices of healthier food relative to unhealthy food were positively associated with insulin resistance (Kern et al., 2018). A Canadian population-based prospective cohort study found that food insecurity is independently associated with increased incident diabetes risk, even after adjustment for a broad set of measured confounders (Tait et al., 2018). |
| 17 | Money spent on eating out | Question from the NHANES questionnaire: “During the past 30 days, how much money {did your family/did you} spend on eating out? Please include money spent in cafeterias at work or at school or on vending machines, for all family members.” Coded as dollar amount of money spent in 30 days. | A cross-sectional study reported a relationship between the frequency of away-from-home meals (AFHs) and T2DM in the rural Chinese population. An excessive frequency of AFHs was likely to increase the prevalence of T2DM. It also reported that BMI partially mediates the effects of the frequency of AFHs on T2DM (Wang et al., 2020). An analysis of two prospective cohort studies showed that frequent consumption of meals prepared at home is associated with a lower risk of developing T2D, which is partly attributable to less weight gain linked with this dining behavior (Zong et al., 2016). |
| 18 | Money spent on carryout/delivered foods | Question from the NHANES questionnaire: “During the past 30 days, how much money {did your family/did you} spend on food carried out or delivered? Please do not include money you have already told me about.” Coded as dollar amount of money spent in 30 days. | A cross-sectional study of 347551 UK Biobank adult participants revealed that access to ready-to-eat food environments was positively associated with type 2 diabetes (Sarkar et al., 2018). In a nation-wide Japanese cohort, fast eating was a strong risk factor for new-onset diabetes (Kudo et al., 2019). |
| 19 | How healthy is the diet | Question from the NHANES questionnaire: “Next I have some questions about {your/SP?s} eating habits. In general, how healthy is {your/his/her} overall diet? Would you say…” Reverse coded as 1 = poor, 2 = fair, 3 = good, 4 = very good, 5 = excellent and modelled as numeric. | A cohort study concluded that high-quality diet, especially maintained over the long term and in conjunction with leisure-time exercise, is associated with lower risk of type 2 diabetes among urban Chinese adults (Yu et al., 2018). Analysis of 3 US prospective cohorts revealed that improvement in overall diet quality is associated with a lower risk of type 2 diabetes, whereas deterioration in diet quality is associated with a higher risk. It added that the association between diet quality changes and diabetes risk is only partly explained by body weight changes (Ley et al., 2016). |
| 20 | Past 30-day milk product consumption | Question from the NHANES questionnaire: “Now I'm going to ask a few questions about milk products. Do not include their use in cooking. In the past 30 days, how often did {you/SP} have milk to drink or on {your/his/her} cereal? Please include chocolate and other flavored milks as well as hot cocoa made with milk. Do not count small amounts of milk added to coffee or tea. Would you say...” Recoded as 0 = never, 1 = Rarely-less than once a week/ Varied, 2 = Sometimes-once a week or more, but less than once a day, or 3 = Often-once a day or more? And modelled as numeric. | A prospective study revealed that a dietary pattern that incorporates higher low-fat dairy products may lower the risk of type 2 diabetes in middle-aged or older women (Liu et al., 2006). Results from 3 large prospective cohorts of US men and women reported that increasing yogurt consumption was associated with a moderately lower risk of T2D, whereas increasing cheese consumption was associated with a moderately higher risk among US men and women. Our study suggests that substituting yogurt or reduced-fat milk for cheese is associated with a lower risk of T2D (Drouin-Chartier et al., 2019). A systematic review and dose-response meta-analysis found that A modest increase in daily intake of dairy products such as low-fat dairy, cheese and yogurt may contribute to the prevention of T2DM (Gao et al., 2013). Analysis of 3 cohorts of US adults and an updated meta-analysis concluded that higher intake of yogurt is associated with a reduced risk of T2D, whereas other dairy foods and consumption of total dairy are not appreciably associated with incidence of T2D (Chen et al., 2014). |
| 21 | Regular milk use 5 times per week | Question from the NHANES questionnaire: “The next question is about regular milk use. A regular milk drinker is someone who uses any type of milk at least 5 times a week. Using this definition, which statement best describes {you/SP}?...” Recoded as 0 = {I've/He's/She's} never been a regular milk drinker; 1 = {My/His/Her} milk drinking has varied over {my/his/her} life-sometimes {I've/he's/she's} been a regular milk drinker; 2 = {I've/He's/She's} been a regular milk drinker for most or all of {my/his/her} life, including {my/his/her} childhood and modelled as numeric. | See no:20 above. |
| 22 | # of meals not home prepared | Question from the NHANES questionnaire: “Next I'm going to ask you about meals. By meal, I mean breakfast, lunch and dinner. During the past 7 days, how many meals {did you/did SP} get that were prepared away from home in places such as restaurants, fast food places, food stands, grocery stores, or from vending machines? {Please do not include meals provided as part of the school lunch or school breakfast./Please do not include meals provided as part of the community programs you reported earlier.}” | See no:17 &18 above. |
| 23 | # of meals from fast food or pizza place | Based on the NHANES questionnaire: “How many of those meals {did you/did SP} get from a fast-food or pizza place?” | A study investigating the association between reported fast-food habits and changes in bodyweight and insulin resistance over a 15-year period in the USA, found fast-food consumption has strong positive associations with weight gain and insulin resistance, suggesting that fast food increases the risk of obesity and type 2 diabetes (Pereira et al., 2005). A cross-sectional analysis of 10 461 participants revealed that increased exposure to fast-food outlets is associated with increased risk of type 2 diabetes and obesity (Bodicoat et al., 2015). See also no:17 &18 above. |
| 24 | # of ready-to-eat foods in past 30 days | Question from the NHANES questionnaire: “Some grocery stores sell "ready to eat" foods such as salads, soups, chicken, sandwiches and cooked vegetables in their salad bars and deli counters. During the past 30 days, how often did {you/SP} eat "ready to eat" foods from the grocery store? Please do not include sliced meat or cheese you buy for sandwiches and frozen or canned foods.” | See no: 17, 18, 22, 23 above. |
| 25 | # of frozen meals/pizza in past 30 days | Question from the NHANES questionnaire “During the past 30 days, how often did you {SP} eat frozen meals or frozen pizzas? Here are some examples of frozen meals and frozen pizzas.” | See no: 17, 18, 22, 23 above. |
| 26 | HH Worried run out of food | Question from the NHANES questionnaire “Now I am going to read you several statements that people have made about their food situation. For these statements, please tell me whether the statement was often true, sometimes true, or never true for {you/your household} in the last 12 months, that is since last {DISPLAY CURRENT MONTH}. The first statement is . . . {I/we} worried whether {my/our} food would run out before {I/we} got money to buy more.” 1 = never true, 2 = sometimes true, 3 = often true. Modelled as numeric. | An association between food security and diabetes risk has been reported in a number of studies (Fitzgerald et al., 2011; Seligman et al, 2007). Also see no: 14, 15, 16 above. |
| 27 | HH Food didn't last | Question from the NHANES questionnaire “[The next statement is . . .] The food that {I/we} bought just didn't last, and {I/we} didn't have money to get more.” Modelled as numeric. | See no: 14, 15, 16, 26 above. |
| 28 | HH Couldn't afford balanced meals | Question from the NHANES questionnaire “[The next statement is . . .] {I/we} couldn't afford to eat balanced meals.” 1 = often true, 2 = sometimes true, 3 = never true. 1 = never true, 2 = sometimes true, 3 = often true. Modelled as numeric. | See no: 14, 15, 16, 26 above. |
| 29 | Household food security category | Household food security category for last 12 months. Reverse coded as 1 = HH very low food security: 6-10 (HH w/o child) / 8-18 (HH w/ child), 2 = HH low food security: 3-5 (HH w/o child) / 3-7 (HH w/ child), 3 = HH marginal food security: 1-2, 4 = HH full food security: 0. Modelled as numeric. | See no: 14, 15, 16, 26 above. |
| 30 | Adult food security category | Adult food security category for last 12 months. Reverse coded as 1 = AD very low food security: 6-10, 2 = AD low food security: 3-5, 3 = AD marginal food security: 1-2, 4 = AD full food security: 0. Modelled as numeric. | See no: 14, 15, 16, 26 above. |
| 31 | Weight | Weight (kg); objectively measured. | A prospective cohort revealed that at even average weight, women are at increased risk of clinical non-insulin-dependent diabetes and that the relation between body mass index and risk of diabetes is continuous (Colditz et al., 1990). Another prospective cohort study indicated that the excess risk for diabetes with even modest and typical adult weight gain is substantial. These findings support the importance of maintaining a constant body weight throughout adult life (Colditz et al., 1995). A meta-analysis indicated that there exists a relation between birth weight and later-life risk of type 2 diabetes which is not linearly inverse but U-shaped (Harder et al., 2007). |
| 32 | Standing height | Standing height (cm); objectively measured | A case-cohort study indicated inverse associations between height and risk of type 2 diabetes, which was largely related to leg length among men. The inverse associations may be partly driven by lower liver fat content and a more favorable cardiometabolic profile (Wittenbecher et al., 2019). A study investigating the association between stature-related measurements (height, leg length, and leg length–to–height ratio) and adiposity, insulin resistance, and glucose intolerance, found that adult markers of prepubertal growth, especially leg length–to–height ratio, are associated with adiposity, insulin resistance, and type 2 diabetes in the general U.S. population (Asao et al., 2006). |
| 33 | Body mass index | Body mass index (kg/m^2^); objectively measured | A case–control study nested in an electronic health records system in the United States revealed that BMI is strongly and independently associated with the risk of being diagnosed with T2D. The incremental association of BMI category on the risk of T2D is stronger for people with a higher BMI relative to people with a lower BMI (Ganz et al., 2014). A study on the association between body mass index history and risk of type 2 diabetes revealed weight gain in early adulthood is related to a higher risk and earlier onset of type 2 diabetes than is weight gain between 40 and 55 y of age (Schienkiewitz et al., 2006). |
| 34 | Upper leg length | Upper leg length (cm); objectively measured | A prospective cohort found that leg length is inversely and independently related to an increased risk of diabetes in middle-age white men and women but not in African-Americans. This sex–race heterogeneity suggests that nutritional and environmental factors in childhood may modify this risk through different pathways (Weitzman et al., 2010). A cross-sectional study revealed leg length is the component of stature related to insulin resistance and coronary heart disease risk, suggesting that pre-adult influences are important in the etiology of coronary heart disease and insulin resistance (Smith et al., 2001). Another cohort study reported that shorter legs were independently associated with lower insulin sensitivity and β-cell function, suggesting that early childhood deprivation may increase the risk of developing diabetes (Johnston et al., 2013). |
| 35 | Upper arm length | Upper arm length (cm); objectively measured. | A cross-sectional study reported associations between arm lengths and type 2 diabetes prevalence supporting a role for factors that determine bone growth or their correlates in the development of this condition. Specifically, total arm length and upper arm length were inversely related to diabetes (Smits et al., 2012). |
| 36 | Arm circumference | Arm circumference (cm); objectively measured. | A greater mid-upper arm circumference was positively associated with higher risks of several cardiometabolic disorders and subclinical atherosclerosis in Chinese adults (Hou et al., 2019). A study revealed that, among Chinese subjects with type 2 diabetes, mid-upper arm circumference is a simple and effective tool for the determination of central obesity and insulin resistance (Zhu et al., 2020). |
| 37 | Waist circumference (cm) | Waist circumference (cm); objectively measured. | An analysis of NHANES 1999-2004 revealed that waist circumference predicted diabetes beyond that explained by traditional cardiometabolic risk factors and BMI. It was concluded that the findings lend critical support for the recommendation that waist circumference can be a routine measure for identification of the high-risk, abdominally obese patient (Janiszewski et al., 2007). A meta-analysis comparing body mass index, waist circumference, and waist/hip ratio in predicting incident diabetes estimated that the pooled relative risks for incident diabetes were 1.87 (95% confidence interval (CI): 1.67, 2.10), 1.87 (95% CI: 1.58, 2.20), and 1.88 (95% CI: 1.61, 2.19) per standard deviation of body mass index, waist circumference, and waist/hip ratio, respectively, demonstrating that these three obesity indicators have similar associations with incident diabetes (Vazquez et al., 2007). |
| 38 | Current self-reported height (inches) | Question from the NHANES questionnaire: “These next questions ask about {your/SP's} height and weight at different times in {your/his/her} life. How tall {are you/is SP} without shoes?” | See no: 32 above. |
| 39 | Current self-reported weight (pounds) | Based on the question from NHANES questionnaire: “How much {do you/does SP} weigh without clothes or shoes?” | See no: 31 above. |
| 40 | Self-reported weight-1 year ago (pounds) | Based on the question from NHANES questionnaire: “How much did {you/SP} weigh a year ago? | A nation-wide cohort study investigating the effect of weight changes on the incidence of type 2 diabetes in Korea reported that weight loss was significantly associated with lower risk for diabetes both in non-obese and obese Koreans, but particularly in the non-obese (Kim et al., 2018). A study investigating the association between weight change and risk of type 2 diabetes and whether initial weight modifies the association, found that a low initial BMI does not ameliorate the increase in risk of type 2 diabetes with weight gain. And the avoidance of weight gain, even among lean individuals, is important to reduce the risk of this disease (Oguma et al., 2005). A prospective cohort indicated that weight gain was associated with substantially increased risk of diabetes among overweight adults, and even modest weight loss was associated with significantly reduced diabetes risk. It concluded that minor weight reductions may have major beneficial effects on subsequent diabetes risk in overweight adults at high risk of developing diabetes (Resnick et al., 2010). Findings from a prospective cohort study examining the influence of weight change on type 2 diabetes incidence indicated a strong positive relationship, supporting the current public-health recommendations for weight control and particularly among ethnic groups at high risk for diabetes (Morimoto et al., 2011). |
| 41 | Self-reported greatest weight (pounds) | Based on the question from the NHANES questionnaire: “Up to the present time, what is the most {you have/SP has} ever weighed?” | See no: 40 above. |
| 42 | Age when heaviest weight | Question from the NHANES questionnaire: “How old {were you/was SP} then? [If you don't know {your/his/her} exact age, please make your best guess.]” | See no:39,40, 41 above. |
| 43 | Salt used in preparation? | Question from the NHANES questionnaire: “How often is ordinary salt or seasoned salt added in cooking or preparing foods in your household? Is it never, rarely, occasionally, or very often?” Coded as 1 = never, 2 = rarely, 3 = occasionally, 4 = very often. Modelled as numeric. | See no:6 above. |
| 44 | Total # of dietary supplements taken | Based on the NHANES questionnaire. Total # of dietary supplements taken. Includes all supplements and the antacids reported with supplements, but not antacids reported with medications. | See no:12 above. |
| 45 | Total # of antacids taken | Based on the NHANES questionnaire. Total # of antacids taken. Includes all antacids reported with medications. | See no:13 above. |
| 46 | Total number of foods reported | Total number of foods reported in the individual foods file. Two-day average was taken where reported. | A multi-center cross-sectional revealed that between-food group variety may exert beneficial effects on glucose metabolism and partially explains the inverse association of the “rice, pasta, meat and fish” dietary pattern with T2D (Danquah et al., 2018). A prospective cohort study found that a diet characterized by regular consumption of all five food groups examined (dairy products, fruits, vegetables, meat and alternatives, and grains) and by greater variety of dairy, fruit, and vegetable subtypes, appears important for a reduced risk of diabetes (Conklin et al., 2016). |
| 47 | Energy | Dietary energy (kcal). Two-day average was calculated where reported. | A cross-sectional found that higher total energy intake was strongly associated with high levels of insulin resistance and may help to explain emerging type 2 diabetes risk in childhood. It recommended further studies to establish whether reducing energy intake produces sustained favorable changes in insulin resistance and circulating glucose levels (Donin et al., 2014). Another study reported that higher protein and total energy intakes (calibrated) appear to be associated with a substantially increased diabetes risk that may be mediated by an increase in body mass over time (Tinker et al., 2011). A study reported that the common SNP rs17782313 near MC4R gene was significantly associated with higher intakes of total energy and dietary fat. In addition, the SNP was related to greater long-term weight change and increased risk of diabetes in women (Qi et al., 2008). A population-based prospective study reported that an energy-dense diet may be associated with increased risk of development of diabetes, independent of baseline obesity, highlighting that the potential public health impact of a low–energy-dense diet on reducing the risk of diabetes deserves further study (Wang et al., 2008). |
| 48 | Protein | Dietary protein content (gm). Two-day average was calculated where available. | A prospective cohort study found that diets high in total as well as animal protein are associated with an increased diabetes risk. Consumption of energy from protein at the expense of energy from either carbohydrates or fat may similarly increase diabetes risk. This finding indicates that accounting for protein content in dietary recommendations for diabetes prevention may be useful (Sluijs et al., 2010). A cross-sectional study among South Asian Indians revealed that higher level of protein intake was associated with increased odds of diabetes (Wang et al., 2010). A dose-response meta-analysis of prospective studies found that the consumption of protein particularly animal protein may be associated with an increased risk of type 2 diabetes (Zhao et al., 2019). |
| 49 | Carbohydrate | Dietary carbohydrate (gm). Two-day average was calculated where available. | A prospective cohort study revealed that a higher carbohydrate intake at the expense of protein and PUFA might be associated with decreased diabetes risk (Schulze et al., 2008). A dose-response meta-analysis of prospective studies on the association between carbohydrates, glycemic index (GI), and glycemic load (GL) and type 2 diabetes found protective effects of low dietary GI and GL, quantifying the range of intakes associated with lower risk. Dose–response trends were linear for GI and GL but more complex for total carbohydrate intake (Greenwood et al., 2013). |
| 50 | Total sugars | Dietary total sugars (gm). Two-day average was calculated where available. | As per a systematic review, data from prospective cohort studies published in the years 2000–2011 suggest that sugar-sweetened beverages probably increase the risk of type 2 diabetes (Sonestedt et al., 2012). A cross-sectional study revealed that high (>20%) energy intake from total sugar may be associated with an increased risk of metabolic syndrome, a strong risk factor for type 2 diabetes (Seo et al., 2019). |
| 51 | Dietary fiber | Dietary fiber content (gm). Two-day average was calculated where available. | A dose-response meta-analysis of prospective studies revealed t the intakes of dietary fiber may be inversely associated with risk of type 2 diabetes (Yao et al., 2014). A randomized controlled trial found that high-fiber, low-fat diet predicts long-term weight loss and decreased type 2 diabetes risk (Lindström et al., 2006). |
| 52 | Total fat | Dietary total fat (gm). Two-day average was calculated where available. | A prospective cohort study revealed that total and saturated fat intake were associated with a higher risk of type 2 diabetes, but these associations were not independent of BMI (Van Dam et al., 2002). |
| 53 | Total saturated fatty acids | Dietary total saturated fatty acids (gm). Two-day average was calculated where available. | A study revealed that the proportional saturated fatty acid composition of plasma is positively associated with the development of diabetes. Findings with the use of this biomarker suggest indirectly that the dietary fat profile, particularly that of saturated fat, may contribute to the etiology of diabetes (ARIC Study Investigators, 2003). |
| 54 | Total monounsaturated fatty acids | Dietary total monounsaturated fatty acids (gm). Two-day average was calculated where available. | A study found that individualized a hypocaloric, high‐fat diet enriched with MUFA (M) and conventional diet (C) were successful in improving metabolic and anthropometric parameters in both the obese non‐diabetic and the type 2 diabetic subjects (Brunerova et al., 2007). A study reported that, after adjusting for confounding factors, total saturated fatty acids among other biomarkers, significantly predicted the worsening of glycaemia and incident type 2 diabetes (Lankinen et al., 2015). |
| 55 | Total polyunsaturated fatty acids (gm) | Dietary total polyunsaturated fatty acids (gm). Two-day average was calculated where available. | A prospective, population-based revealed that the serum long-chain omega-3 PUFA concentration, an objective biomarker for fish intake, was associated with long-term lower risk of type 2 diabetes (Virtanen et al., 2014). |
| 56 | Cholesterol | Dietary cholesterol (mg). Two-day average was calculated where available. | An investigation of cholesterol metabolism in obesity with and without diabetes found that cholesterol absorption efficiency was lower and cholesterol synthesis was higher in obese subjects with diabetes than in those without diabetes, suggesting that diabetes modulates cholesterol metabolism more than obesity alone (Simonen et al., 2002). A study investigating associations between reported intakes of dietary fat and incident type 2 diabetes found an inverse relation between incident type 2 diabetes and vegetable fat and substituting polyunsaturated fatty acids for saturated fatty acids and cholesterol (Meyer et al., 2001). |
| 57 | Vitamin E as alpha-tocopherol | Dietary vitamin E as alpha-tocopherol (mg). Two-day average was calculated where available. | An increased risk of non-insulin dependent diabetes mellitus at low plasma vitamin E concentrations was reported by a four year follow up study in men (Salonen et al., 1995). A prospective cohort study confirmed the hypothesis that development of type 2 diabetes may be reduced by the intake of antioxidants in the diet (Montonen et al., 2004). Another study revealed that a protective effect of vitamin E may exist within the range of intake available from food. This effect may go undetected within studies of high-dose supplement use, which appears to hold no additional protective benefit (Mayer-Davis et al., 2002). |
| 58 | Added alpha-tocopherol (Vitamin E) | Dietary added alpha-tocopherol (Vitamin E) (mg). Two-day average was calculated where available. | See no:57 above. |
| 59 | Retinol | Dietary retinol (mcg). Two-day average was calculated where available. | A study illustrated the antihyperglycemic and antioxidant potential of vitamin A in vivo, which has potential to serve as a dietary intervention in type 2 diabetes (Meerza et al., 2016). Vitamin A regulates insulin release and energy homeostasis (Iqbal & Naseem, 2015). In recent years, evidence has demonstrated that vitamin A can also regulate metabolic pathways implicated in the pathogenesis of obesity and diabetes. This has increased interest in the possible anti-obesity and antidiabetic properties of natural and synthetic vitamin A derivatives (Trasino & Gudas, 2015). |
| 60 | Vitamin A, RAE | Dietary Vitamin A as retinol activity equivalents (mcg). Two-day average was calculated where available. | See no:59 above. |
| 61 | Alpha-carotene | Dietary alpha-carotene (mcg). Two-day average was calculated where available. | A prospective cohort study showed that diets high in β-carotene and α-carotene are associated with reduced type 2 diabetes in generally healthy men and women (Sluijs et al., 2015). A cross-sectional study revealed an advantageous association of carotenoids, which are markers of fruit and vegetable intake, with glucose metabolism in men at high risk of type 2 diabetes (Ylönen et al., 2003). A follow-up study found that eating a diet rich in carotenoids, especially provitamin A carotenoids, might help prevent the development of type 2 diabetes in Japanese individuals (Sugiura et al., 2015). |
| 62 | Beta-carotene | Dietary beta-carotene (mcg). Two-day average was calculated where available. | See no:61 above. |
| 63 | Beta-cryptoxanthin | Dietary beta-cryptoxanthin (mcg). Two-day average was calculated where available. | It was found that beta-cryptoxanthin, a natural carotenoid with antioxidant properties, ameliorates metabolic risk factors by regulating NF-κB and Nrf2 pathways in insulin resistance induced by high-fat diet in rodents (Sahin et al., 2017). Double-blind, placebo-controlled parallel trials found beneficial effects of a beta-cryptoxanthin-containing beverage on body mass index and visceral fat in pre-obese men (Iwata et al., 2018). |
| 64 | Lycopene | Dietary lycopene (mcg). Two-day average was calculated where available. | A study reported that all investigated serum carotenoids (α-carotene, β-carotene, cryptoxanthin, lutein/zeaxanthin, and lycopene) were inversely related to fasting serum insulin after adjustment for confounders (p < 0.05 for each carotenoid) suggesting a possible role for carotenoids in the pathogenesis of insulin resistance and diabetes (Ford et al., 1999). A review reported inverse associations of serum β-cryptoxanthin with the risks for atherosclerosis, insulin resistance, liver dysfunction, metabolic syndrome, low bone mineral density, and oxidative stress (Sugiura, 2015). A case-control study reported higher β-cryptoxanthin concentrations among healthy controls compared to diabetic subjects (Olmedilla et al., 1997). |
| 65 | Lutein + Zeaxanthin | Dietary lutein + zeaxanthin (mcg). Two-day average was calculated where available. | See no:59-64 above. |
| 66 | Thiamin (vitamin B1) | Dietary thiamin (Vitamin B1) (mg). Two-day average was calculated where available. | A case-control study reported deficiency of vitamin B6 (PLP, pyridoxine, pyridoxal) and vitamin B1 (thiamine) was prevalent in type 2 diabetes (Nix et al., 2015). Thiamine is an essential cofactor in carbohydrate metabolism and individuals with diabetes are thiamine deficient (Page et al., 2011). |
| 67 | Riboflavin (vitamin B2) | Dietary riboflavin (Vitamin B2) (mg). Two-day average was calculated where available. | A study reported that insufficient riboflavin intake may contribute to development of cardiometabolic disorder, particularly in women (Shin & Kim, 2019). An ameliorative effect of riboflavin on hyperglycemia, oxidative stress and DNA damage in type-2 diabetic mice has been reported (Alam et al., 2015). |
| 68 | Niacin | Dietary niacin (mg). Two-day average was calculated where available. | A meta-analysis of randomized controlled trials revealed that niacin therapy is associated with a moderately increased risk of developing diabetes regardless of background statin or combination laropiprant therapy (Goldie et al., 2016). An analysis of NHANES 2007-2014 revealed that linear inverse relationship between dietary vitamin B1, niacin, B6, DFE and metabolic syndrome, and a non-linear inverse relationship between dietary vitamin B2 and metabolic syndrome, suggesting higher intake of vitamin B1, B2, niacin, B6 and DFE were all associated with reduced risk of metabolic syndrome (Wu et al., 2020). |
| 69 | Vitamin B6 | Dietary vitamin B6 (mg). Two-day average was calculated where available. | Epidemiological and experimental studies indicated an evident inverse association between vitamin B6 levels and diabetes (Mascolo & Verni, 2020). A follow-up study showed that higher intakes of vitamin B2 and vitamin B6 are associated with a lower T2D risk (Braun et al., 2019). |
| 70 | Total folate | Dietary total folate (mcg). Two-day average was calculated where available. | A 30-year follow-up study found that the intake of folate in young adulthood was inversely associated with diabetes incidence in midlife among Americans, which may be partially explained by mechanisms related to homocysteine level, insulin sensitivity, and systemic inflammation (Zhu et al., 2020). A follow-up study revealed a prospective association between dietary folate intake and type 2 diabetes risk among Korean adults aged 40 years or older (Hong et al., 2017). A prospective cohort study found that higher habitual intakes of supplemental folate before pregnancy were significantly associated with lower GDM risk (Li et al., 2019). A systematic review and meta-analysis of randomized controlled trials found a potential benefit of folate on insulin resistance and glycemic control (Zhao et al., 2018). |
| 71 | Folic acid | Dietary folic acid (mcg). Two-day average was calculated where available. | See no:70 above. |
| 72 | Food folate | Dietary food folate (mcg). Two-day average was calculated where available. | See no:70 above. |
| 73 | Folate, DFE | Folate as dietary folate equivalents (mcg). Two-day average was calculated where available. | See no:70 above. |
| 74 | Total choline | Dietary total choline (mg). Two-day average was calculated where available. | A prospective cohort study revealed that higher total dietary choline intake, especially phosphatidylcholine, was associated with lower type 2 diabetes risk among men (Virtanen et al., 2020). An observational prospective study among Norwegian patients found that systemic and urinary concentrations of several choline metabolites were associated with risk of incident type 2 diabetes, and relevant biomarkers may improve risk prediction (Svingen et al., 2016). |
| 75 | Vitamin B12 | Dietary vitamin B12 (mcg). Two-day average was calculated where available. | A case-control study reported that vitamin D, B12, and folic acid levels were low and poor vitamin D and B12 status were associated with insulin resistance in nondiabetic obese patients (Cigerli et al., 2016). A cross-sectional study found that lower circulating B12 is associated with higher obesity and insulin resistance during pregnancy in a non-diabetic White British population (Knight et al., 2015). A study reported that low plasma vitamin B12 in pregnancy is associated with gestational ‘diabesity’ and later incident diabetes (Krishnaveni et al., 2009). A primary-care based cross-sectional study revealed that low vitamin B12 level was associated with obesity, overweight, and metabolic syndrome (Baltaci et al., 2013). In another study, lower vitamin B12 in obese adolescents was associated with clinical features of insulin resistance (Ho et al., 2014).A systematic review and meta-analysis revealed that vitamin B12 insufficiency is associated with increased risk of gestational diabetes mellitus, which in turn, is a major risk factor of T2DM (Kouroglou et al., 2019). |
| 76 | Added vitamin B12 | Added vitamin B12 (mcg) in the diet. Two-day average was calculated where available. | See no:75 above. |
| 77 | Vitamin C | Dietary vitamin C (mg). Two-day average was calculated where available. | A follow-up study reported that higher plasma vitamin C level is associated with a substantially decreased risk of diabetes (Harding et al., 2008). Another study reported that dietary vitamin C intake reduces the risk of type 2 diabetes in Chinese Adults, which may be partly mediated by inhibiting or ameliorating oxidative stress and insulin resistance (Zhou et al., 2016). |
| 78 | Vitamin D (D2 + D3) | Dietary vitamin D (D2 + D3) (mcg). Two-day average was calculated where available. | A follow-up study reported a joint action of dietary calcium and vitamin D against type 2 diabetes (Kirii et al., 2009). A cross-sectional study reported vitamin D insufficiency is associated with diabetes risk (Nsiah-Kumi et al., 2012). A systematic review revealed that Vitamin D may play a role in type 2 diabetes (Mitri et al., 2011). A nested case-control study concluded that Plasma 25-hydroxyvitamin D concentration was associated with lower risk of incident type 2 diabetes in women (Pittas et al., 2010). A pooled analysis of two nested case-control studies found high vitamin D status is protective against type 2 diabetes (Knekt et al., 2008). A meta-analysis of prospective studies revealed an inverse and significant association between circulating 25(OH)D levels and risk of type 2 diabetes across a broad range of blood 25(OH)D levels in diverse populations (Song et al., 2013). A Mendelian randomization study reported that genetic variants associated with low plasma 25(OH)D concentrations are associated with type 2 diabetes and low plasma 25(OH)D concentrations might be a modest mediator between obesity and increased risk of diabetes (Afzal et al., 2014). |
| 79 | Vitamin K | Vitamin K (mcg). Two-day average was calculated where available. | Beneficial role of vitamin K supplementation on insulin sensitivity, glucose metabolism, and the reduced risk of type 2 diabetes has been summarized in a review (Manna & Kalita, 2016). Vitamin K_2_ improved insulin sensitivity through involvement of vitamin K-dependent-protein osteocalcin, anti-inflammatory properties, and lipid-lowering effects (Li et al., 2018). A prospective cohort study showed that both phylloquinone (vitamin K_1_) and menaquinones (vitamin K_2_) intakes may be associated with a reduced risk of type 2 diabetes (Beulens et al., 2010) |
| 80 | Calcium | Dietary calcium (mg). Two-day average was calculated where available. | A population-based prospective cohort study revealed that calcium and magnesium intakes may protect against the development of T2D (Villegas et al., 2009). A study found that intakes of calcium and dairy products may be associated with lower prevalence of the metabolic syndrome in middle-aged and older women (Liu et al., 2005). A large prospective study found results that suggested a potential beneficial role for both vitamin D and calcium intake in reducing the risk of type 2 diabetes (Pittas et al., 2006). A follow-up study reported that higher dietary calcium intake was associated with a decreased risk of T2D development (Kim et al., 2018). |
| 81 | Phosphorus | Dietary phosphorus (mg). Two-day average was calculated where available. | High dietary phosphorus intake is associated with an increased risk of type 2 diabetes in a large prospective cohort study (Mancini et al., 2018). A follow-up study found that serum phosphorus was related with the incidence of metabolic syndrome (Jhuang et al., 2019). |
| 82 | Magnesium | Dietary magnesium (mg). Two-day average was calculated where available. | Among white participants, low serum magnesium level is a strong, independent predictor of incident type 2 diabetes as per a prospective study (Kao et al., 1999). A follow-up study reported a protective role of higher intake of magnesium in reducing the risk of developing type 2 diabetes, especially in overweight women (Song et al., 2004). A dose–response meta-analysis of prospective cohort studies found that increasing dietary magnesium intake is associated with a reduced risk of diabetes. (Fang et al., 2016). |
| 83 | Iron | Dietary iron (mg). Two-day average was calculated where available. | High levels of dietary iron may impart diabetes risk (Simcox & McClain, 2013). In a cross-sectional study, associations among higher serum ferritin level, higher heme iron intake, and elevated risk of diabetes were found (Li et al., 2008). A systematic review and meta-analysis revealed that higher heme iron intake and increased body iron stores were significantly associated with a greater risk of T2DM (Bao et al., 2012). A prospective cohort study reported that heme-iron intake from red meat sources is positively associated with the risk of type 2 diabetes (Jiang et al., 2004). A prospective study reported that greater dietary heme-iron intake and/or supplemental iron were associated with an increased risk of type 2 diabetes, especially amongst those who drink alcohol (Lee et al., 2004). |
| 84 | Zinc | Dietary zinc (mg). Two-day average was calculated where available. | A prospective study concluded that higher zinc intake may be associated with a slightly lower risk of type 2 diabetes in women (Sun et al., 2009). A follow-up study reported that higher total dietary zinc intake and high zinc/iron ratio are associated with lower risk of type 2 diabetes in women (Vashum et al., 2013). A prospective study reported that zinc supplementation and a high zinc to iron intake ratio may lower the risk of T2D, but these associations could be modified by obesity and the SLC30A8 genotype (Drake et al., 2017). |
| 85 | Copper | Dietary copper (mg). Two-day average was calculated where available. | A prospective study concluded that dietary intakes of copper was associated with a higher risk of T2DM (Eshak et al., 2018). A follow-up study reported that a lower dietary Cu-Zn ratio intake is associated with a lower T2D risk, especially among obese women (Laouali et al., 2020). |
| 86 | Sodium | Dietary sodium (mg). Two-day average was calculated where available. | A study found that moderate salt restriction aggravates both systemic and vascular insulin resistance (Feldman & Schmidt, 1999). A study reported that low-salt diet increases insulin resistance in healthy subjects (Garg et al., 2011). A case-control study found a possible relationship between additional adding of salt to prepared meals and an increased risk of type 2 diabetes (Radzeviciene & Ostrauskas, 2017). A study found results suggesting that salt intake is a potential risk factor for obesity independent of energy intake (Ma et al., 2015). |
| 87 | Potassium | Dietary potassium (mg). Two-day average was calculated where available. | A prospective cohort study found that serum potassium level is an independent predictor of incident DM (Chatterjee et al., 2010). Another follow-up study found that low dietary potassium is associated with increased risk of incident diabetes in African-Americans (Chatterjee et al., 2012). |
| 88 | Selenium | Dietary selenium (mcg). Two-day average was calculated where available. | A prospective study found that increased dietary selenium intake was associated with an increased risk of type 2 diabetes (Stranges et al., 2010). A cross-sectional study found that there was a significant positive correlation between dietary selenium intake and the prevalence of diabetes (Wei et al., 2015). A systematic review and meta-analysis found, as per results from both nonexperimental and experimental studies, that selenium may increase the risk of type 2 diabetes across a wide range of exposure levels (Vinceti et al., 2018). |
| 89 | Caffeine | Dietary caffeine (mg). Two-day average was calculated where available. | A prospective cohort study found that moderate consumption of both caffeinated and decaffeinated coffee may lower risk of type 2 diabetes in younger and middle-aged women (Van Dam et al., 2006). A retrospective cohort study found that consumption of green tea, coffee, and total caffeine was associated with a reduced risk for type 2 diabetes (Iso et al., 2006). Coffee intake was associated with a lower risk of T2D, as per a prospective cohort study (Bhupathiraju et al., 2013). A meta-analysis of prospective studies concluded that coffee and caffeine intake might significantly reduce the incidence of T2DM (Jiang et al., 2014). A nested case-control study found results suggesting that sex-hormone binding globulin may account for the inverse association between coffee consumption and type 2 diabetes risk among postmenopausal women (Goto et al., 2011). Findings from a study investigating long-term effects of coffee and caffeine intake on the risk of pre-diabetes and type 2 diabetes in a population with low coffee consumption indicated that coffee drinking may have favorable effect in prevention of pre-diabetes and T2D (Mirmiran et al., 2018). A prospective study concluded that coffee consumption was associated with a substantially lower risk of clinical type 2 diabetes (Van Dam & Feskens, 2002). A prospective cohort study reported that long-term coffee consumption is associated with a statistically significantly lower risk for type 2 diabetes (Salazar-Martinez et al., 2004). A prospective cohort study reported that drinking coffee or tea is associated with a lowered risk of type 2 diabetes, which cannot be explained by magnesium, potassium, caffeine or blood pressure effects concluding that total consumption of at least three cups of coffee or tea per day may lower the risk of type 2 diabetes (Van Dieren et al., 2009). |
| 90 | Theobromine | Dietary theobromine (mg). Two-day average was calculated where available. | A study found that theobromine stimulates thermogenesis by inducing white fat browning and activating brown adipocytes, concluding that the consumption of theobromine may be a feasible way to activate thermogenesis and improve systematic lipid metabolism to protect against obesity and other metabolic disorders (Jang et al., 2018). A prospective cohort study reported that consuming moderate amount of chocolate (rich in theobromine) may reduce the risk of diabetes (Greenberg, 2015). |
| 91 | Alcohol | Dietary alcohol (gm). Two-day average was calculated where available. | Alcohol intake is an established risk factor of T2DM. Please see no:115. Since, association between dietary alcohol content and undiagnosed T2DM has not been explored previously, this was included as an independent variable. |
| 92 | Moisture | Dietary moisture (gm). Two-day average was calculated where available. | A cross-sectional study found that higher plain water intake is associated with lower type 2 diabetes risk (Carroll et al., 2015). Another prospective study reported that plain-water intake, per se, was not significantly associated with risk of T2D, although substitution of plain water for sugar-sweetened beverages or fruit juices was estimated to be associated with modestly lower risk of T2D (Pan et al., 2012). As per a follow-up study, self-reported water intake was inversely and independently associated with the risk of developing hyperglycemia (Roussel et al., 2011). We hypothesized a potential association driven by previous findings with regard to water intake, mentioned above. |
| 93 | SFA 4:0 (butanoic) | Dietary SFA 4:0 (Butanoic) (gm). Two-day average was calculated where available. | Although early clinical studies with high levels of medium‐chain saturated fatty acids (MCFA) resulted in increased levels of plasma triacylglycerols and low‐density lipoprotein cholesterol, and reduced levels of high‐density lipoprotein cholesterol compared to diets enriched in unsaturated long-chain fatty acids, these adverse effects have not been observed in more recent studies with smaller more realistic amounts of MCFA (Huth et al., 2010). A prospective study reported that total SFA does not relate to T2D risk and rather, the association may depend on the types and food sources of SFA. Cheese-derived SFA and individual SFA that are commonly found in cheese, were significantly related to lower T2D risks. They could not exclude the higher T2D risks found for soft and liquid fats derived SFA and for substituting SFA with other macronutrients are influenced by residual confounding by trans fatty acids or limited intake variation in polyunsaturated fatty acids and vegetable protein (Liu et al., 2018). A study reported that the intake of dietary *n*-3 fatty acids may be protective against whereas SFA intake may promote insulin resistance (Paquet et al., 2014). Findings from a prospective study suggested that dietary fat composition may modify the risk of T2D incidence (Gaeini et al., 2019). A cross-sectional study found that the relations between fatty acid intakes and markers of type 2 diabetes risk may depend on the dietary sources of the fatty acids, concluding that more epidemiological studies on diet and cardiometabolic disease are needed, addressing possible interactions between nutrients and their dietary sources (Wanders et al., 2017). A prospective study reported that the proportional saturated fatty acid composition of plasma is positively associated with the development of diabetes, suggesting indirectly that the dietary fat profile, particularly that of saturated fat, may contribute to the etiology of diabetes (ARIC Study Investigators, 2003). A narrative review concluded that there is currently insufficient evidence to support current dietary guidelines which consolidate all dietary SFA into a single group of nutrients whose consumption should be reduced, regardless of dietary source, food matrix, and composition (Unger et al., 2019) A prospective study reported that higher serum 2h-SFA (but not fasting SFA) independently predicted diabetes risk (Wang et al., 2018). |
| 94 | SFA 6:0 (hexanoic) | Dietary SFA 6:0 (Hexanoic) (gm). Two-day average was calculated where available. | See no:93 above. |
| 95 | SFA 8:0 (octanoic) | Dietary SFA 8:0 (Octanoic) (gm). Two-day average was calculated where available. | See no:93 above. |
| 96 | SFA 10:0 (decanoic) | Dietary SFA 10:0 (Decanoic) (gm). Two-day average was calculated where available. | See no:93 above. |
| 97 | SFA 12:0 (dodecanoic) | Dietary SFA 12:0 (Dodecanoic) (gm). Two-day average was calculated where available. | See no:93 above. |
| 98 | SFA 14:0 (tetradecanoic) | Dietary SFA 14:0 (Tetradecanoic) (gm). Two-day average was calculated where available. | See no:93 above. |
| 99 | SFA 16:0 (hexadecanoic) | Dietary SFA 16:0 (Hexadecanoic) (gm). Two-day average was calculated where available. | See no:93 above. |
| 100 | SFA 18:0 (octadecanoic) | Dietary SFA 18:0 (Octadecanoic) (gm). Two-day average was calculated where available. | See no:93 above. |
| 101 | MFA 16:1 (hexadecenoic) | Dietary MFA 16:1 (Hexadecenoic) (gm). Two-day average was calculated where available. | A review concluded that dietary monounsaturated fatty acids are protective against metabolic syndrome and cardiovascular disease risk factors (Gillingham et al., 2011). A study reported that dietary fatty acids are differentially associated with fasting versus 2-hour glucose homeostasis with implications for the management of subtypes of prediabetes (Guess et al., 2016). A follow-up study reported that increases of MUFA and soluble fibers intakes promote benefits on glucose metabolism, independently of adiposity, during a realistic lifestyle intervention in at-risk individuals. Mechanisms mediating these processes may include mainly insulin sensitivity improvement (de Barros et al., 2014). A study reported differential effects of monounsaturated, polyunsaturated and saturated fat ingestion on glucose-stimulated insulin secretion, sensitivity and clearance in overweight and obese, non-diabetic humans (Xiao et al., 2006). A study found that β cell function and insulin sensitivity progressively improve in the postprandial state as the proportion of MUFAs with respect to SFAs in dietary fats increases (Lopez et al., 2008). A randomized trial reported that a diet high in monounsaturated fat has a more favorable effect on glucose homeostasis than does the typical Western diet in the short term and may also be more beneficial than the official recommended low-fat diet during a period of weight regain subsequent to weight loss (Due et al., 2008). A study found dietary intake of MUFAs promoted insulin action in the brain with its beneficial effects for cortical activity, locomotion, and sleep, whereas a comparable intake of SFAs acted as a negative modulator of brain activity in mice and humans (Sartorius et al., 2012). |
| 102 | MFA 18:1 (octadecenoic) | Dietary MFA 18:1 (Octadecenoic) (gm). Two-day average was calculated where available. | See no:101 above. |
| 103 | MFA 20:1 (eicosenoic) | Dietary MFA 20:1 (Eicosenoic) (gm). Two-day average was calculated where available. | See no:101 above. |
| 104 | MFA 22:1 (docosenoic) | Dietary MFA 22:1 (Docosenoic) (gm). Two-day average was calculated where available. | See no:101 above. |
| 105 | PFA 18:2 (octadecadienoic) | Dietary PFA 18:2 (Octadecadienoic) (gm). Two-day average was calculated where available. | A meta-analysis of randomized controlled feeding trials provides evidence that dietary macronutrients have diverse effects on glucose-insulin homeostasis. In comparison to carbohydrate, SFA, or MUFA, most consistent favourable effects were seen with PUFA, which was linked to improved glycaemia, insulin resistance, and insulin secretion capacity (Imamura et al., 2016). A prospective study reported that total fat and saturated and monounsaturated fatty acid intakes are not associated with risk of type 2 diabetes in women, but trans fatty acids increase and polyunsaturated fatty acids reduce risk. Substituting nonhydrogenated polyunsaturated fatty acids for trans fatty acids would likely reduce the risk of type 2 diabetes substantially (Salmeron et al., 2001). A systematic review and meta-analysis provided evidence that marine n-3 PUFA have beneficial effects on the prevention of T2D in Asian populations (Zheng et al., 2012). A case-cohort study reported an important inverse association of circulating plant-origin n-3 PUFA (ALA) but no convincing association of marine-derived n3 PUFAs (EPA and DHA) with T2D. Moreover, they highlight that the most abundant n6-PUFA (LA) is inversely associated with T2D. The detection of associations with previously less well-investigated PUFAs points to the importance of considering individual fatty acids rather than focusing on fatty acid class (Forouhi et al., 2016). A prospective study found that higher serum total n–6 PUFA, LA, and AA concentrations and estimated D5D activity were associated with a lower risk of incident T2D, and higher GLA and DGLA concentrations and estimated D6D activity were associated with a higher risk (Yary et al., 2016). |
| 106 | PFA 18:3 (octadecatrienoic) | Dietary PFA 18:3 (Octadecatrienoic) (gm). Two-day average was calculated where available. | See no:105 above. |
| 107 | PFA 18:4 (octadecatetraenoic) | Dietary PFA 18:4 (Octadecatetraenoic) (gm). Two-day average was calculated where available. | See no:105 above. |
| 108 | PFA 20:4 (eicosatetraenoic) | Dietary PFA 20:4 (Eicosatetraenoic) (gm). Two-day average was calculated where available. | See no:105 above. |
| 109 | PFA 20:5 (eicosapentaenoic) | Dietary PFA 20:5 (Eicosapentaenoic) (gm). Two-day average was calculated where available. | See no:105 above. |
| 110 | PFA 22:5 (docosapentaenoic) | Dietary PFA 22:5 (Docosapentaenoic) (gm). Two-day average was calculated where available. | See no:105 above. |
| 111 | PFA 22:6 (docosahexaenoic) | Dietary PFA 22:6 (Docosahexaenoic) (gm). Two-day average was calculated where available. | See no:105 above. |
| 112 | Total plain water drank yesterday | Total plain water drank yesterday (gm) - including plain tap water, water from a drinking fountain, water from a water cooler, bottled water, and spring water. Calculated from water consumption records reported as part of the 24-hour dietary recall interview. Two-day average was calculated where available. | See no:9 & 92 above. A clinical trial reported that, at baseline, circulating levels of copeptin were positively associated with 24-h urine concentration in healthy young subjects with various fluid intakes. Moreover, this study shows, for the first time, that increased water intake over 6 weeks results in an attenuation of circulating copeptin, which is associated with incident type 2 diabetes mellitus (Lemetais et al., 2018). A study found that high water intake and low urine osmolality are associated with favorable metabolic profile at a population level and concluded that low concentrations of the vasopressin marker copeptin is linked to high water intake, low urine osmolality, and a favorable metabolic profile, suggesting that vasopressin lowering lifestyle interventions, such as increased water intake, may promote metabolic health (Brunkwall et al., 2020). |
| 113 | Total tap water drank yesterday | Total tap water drank yesterday (gm) - including filtered tap water and water from a drinking fountain. Calculated from tap water consumption records reported as part of the 24-hour dietary recall interview. Two-day average was calculated where available. | See no:112 above. Also, constituents in tap water may have an impact proportionate to its intake. For example, a population-based study confirmed a previously reported, but frequently questioned, association between exposure to inorganic arsenic and diabetes, and for the first time linked the risk of diabetes to the production of one of the most toxic metabolites of inorganic arsenic, dimethylarsinite (Del Razo et al., 2011). |
| 114 | Total bottled water drank yesterday | Total bottled water drank yesterday (gm). Calculated from bottle water consumption records reported as part of the 24-hour dietary recall interview. Two-day average was calculated where available. | A cross-sectional study reported that drinking bottled water may be associated with higher prevalence of diabetes compared to tap water, potentially mediated by endocrine disruptors in plastic bottles(Joshipura et al., 2018). Also see no:112 above. |
| **Other modifiable/health behavior associated** | | |  |
| 115 | Alcohol use | Question from the NHANES questionnaire: “The next questions are about drinking alcoholic beverages. Included are liquor (such as whiskey or gin), beer, wine, wine coolers, and any other type of alcoholic beverage.In any one year, {have you/has SP} had at least 12 drinks of any type of alcoholic beverage? By a drink, I mean a 12 oz. beer, a 5 oz. glass of wine, or one and half ounces of liquor.” Coded as 1 = no, 2= yes. | A prospective study revealed that high alcohol intake increases diabetes risk among middle-aged men whereas more moderate levels of alcohol consumption do not increase risk of type 2 diabetes in either middle-aged men or women (Kao et al., 2001). A prospective study reported that high daily intake of alcohol, even on only 1–3 days a week, may increase the risk of diabetes in men (Hodge et al., 2006). A follow-up study concluded that nge drinking and high alcohol consumption may increase the risk of type 2 diabetes in women (Carlsson et al., 2003). A systematic review and dose-response meta-analysis of more than 1.9 million individuals from 38 observational studies found that reductions in risk among moderate alcohol drinkers may be confined to women and non-Asian populations. It also concluded that, although based on a minority of studies, there is also the possibility that reductions in risk may have been overestimated by studies using a referent group contaminated by less healthy former drinkers (Knott et al., 2015). A prospective study reported that high alcohol consumption increases the risk of abnormal glucose regulation in men, whilst, in women the associations are more complex: decreased risk with low or medium intake and increased risk with high alcohol intake (Cullmann et al., 2012). A prospective study revealed that alcohol consumption, especially heavy consumption, was an independent risk factor for prediabetes (Zhang et al., 2016). |
| 116 | Donated blood in past 12 months | Question from NHANES questionnaire: “During the past 12 months, that is, since (DISPLAY CURRENT MONTH, DISPLAY LAST YEAR), (have you/has SP) donated blood?” Coded as 1 = no, 2 = yes. | Those who have donated blood reflect a higher overall health than those who have not (Atsma et al., 2011). And a potential association with diabetes risk was assumed. |
| 117 | Blood ever tested for HIV virus? | Question from NHANES questionnaire: “Except for tests {you/SP} may have had as part of blood donations, {have you/has he/has she} ever had {your/his/her} blood tested for the AIDS virus infection?”. Coded as 1 = no, 2 = yes. | A prospective cohort reported that HIV is a risk factor for incident diabetes (De Wit et al., 2008). A cross-sectional study found that sexual minorities may be at increased risk for diabetes than their heterosexual peers (Beach et al., 2018). |
| 118 | Vigorous work activity | Question from the NHANES questionnaire: “Next I am going to ask you about the time {you spend/SP spends} doing different types of physical activity in a typical week. Please answer these questions even if {you do not consider yourself/SP does not consider himself/herself} to be a physically active person. Think first about the time {you spend/SP spends} doing work. Think of work as the things that {you have/SP has} to do such as paid or unpaid work, studying or training, household chores, and yard work. In answering the following questions, 'vigorous-intensity activities' are activities that require hard physical effort and cause large increases in breathing or heart rate, and 'moderate-intensity activities' are activities that require moderate physical effort and cause small increases in breathing or heart rate. Does {your/SP's} work involve vigorous-intensity activity that causes large increases in breathing or heart rate like carrying or lifting heavy loads, digging or construction work for at least 10 minutes continuously?” Coded as 1 = no, 2 = yes. | A prospective study revealed that black women might reduce their risk of developing type 2 diabetes by increasing their time spent walking or engaged in vigorous physical activity and by limiting television watching (Krishnan et al., 2009). A prospective study reported that vigorous exercise significantly reduces diabetes incidence, due in part to the prevention of age-related weight gain and in part to other exercise effects (Williams, 2007). As per the findings of a follow-up study, objectively measured moderate- and vigorous-intensity physical activity but not sedentary time predicts insulin resistance in high-risk individuals (Ekelund et al., 2009). A systematic review and dose–response meta-analysis provides strong evidence for an inverse association between physical activity and risk of type 2 diabetes and found all subtypes of physical activity appear to be beneficial. It concluded that reductions in risk are observed up to 5–7 h of leisure-time, vigorous or low intensity physical activity per week, but further reductions cannot be excluded beyond this range (Aune et al., 2015). A prospective cohort study revealed that moderate-to-vigorous physical activity plays an important role in diabetes, influencing both its incidence and prognosis. A protective effect on incidence was seen for durations of activity below recommendations and a marginal additional benefit was observed at higher durations (Yerramalla et al., 2020). Results from a prospective study confirmed the importance of PA and sedentary behavior on diabetes risk in a multiethnic population and demonstrated potential variations across race/ethnic groups (Joseph et al., 2016). |
| 119 | Moderate work activity | Question from the NHANES questionnaire: “Does {your/SP's} work involve moderate-intensity activity that causes small increases in breathing or heart rate such as brisk walking or carrying light loads for at least 10 minutes continuously?” Coded as 1 = no, 2 = yes. | A prospective study reported that greater physical activity level is associated with substantial reduction in risk of type 2 diabetes, including physical activity of moderate intensity and duration (Hu et al., 1999). A prospective study found that, compared with physical inactivity, any type of physical activity was associated with reduced risk of type 2 diabetes in adults aged 70 years and over, while in adults aged 50 to 69 years, physical activity needed to be vigorous/moderate in intensity to be associated with reduced risk of type 2 diabetes (Demakakos et al., 2010). A systematic review concluded that adherence to recommendations to participate in physical activities of moderate intensity such as brisk walking can substantially reduce the risk of type 2 diabetes (Jeon et al., 2007). |
| 120 | Walk or bicycle | Question from the NHANES questionnaire: “The next questions exclude the physical activity of work that you have already mentioned. Now I would like to ask you about the usual way {you travel/SP travels} to and from places. For example, to work, for shopping, to school. {Do you/Does SP} walk or use a bicycle for at least 10 minutes continuously to get to and from places?” Coded as 1 = no, 2 = yes. | A study reported that equivalent energy expenditures by moderate (walking) and vigorous (running) exercise produced similar risk reductions for hypertension, hypercholesterolemia, diabetes mellitus, and possibly CHD (Williams & Thompson, 2013). A prospective study revealed that commuter and recreational cycling was consistently associated with lower risk of T2D in Danish adults and also provided evidence that late-in-life initiation of or continued engagement in cycling lowers risk of T2D (Rasmussen et al., 2016). A cross-sectional study reported those engaged in active travel and cycling for travel in particular had lower odds of diabetes and lower risk factors for cardiovascular disease compared to those not engaged in active travel (Riiser et al., 2018). |
| 121 | Vigorous recreational activities | Question from the NHANES questionnaire: “The next questions exclude the work and transportation activities that you have already mentioned. Now I would like to ask you about sports, fitness and recreational activities. {Do you/Does SP} do any vigorous-intensity sports, fitness, or recreational activities that cause large increases in breathing or heart rate like running or basketball for at least 10 minutes continuously?” Coded as 1 = no, 2 = yes. | A prospective study reported that regular physical exercise at least once a week and vigorous activity even only once a week at weekends are associated with a decreased risk of T2DM (Okada et al., 2010). A prospective study found that weight training was associated with a significantly lower risk of T2DM, independent of aerobic exercise. And combined weight training and aerobic exercise conferred a greater benefit (Grøntved et al., 2012). |
| 122 | Moderate recreational activities | Question from the NHANES questionnaire: “{Do you/Does SP} do any moderate-intensity sports, fitness, or recreational activities that cause a small increase in breathing or heart rate such as brisk walking, bicycling, swimming, or golf for at least 10 minutes continuously?” Coded as 1 = no, 2 = yes. | A randomized trial comparing short-term high-intensity interval training (HIIT) with moderate-intensity continuous training (MICT) found that ten days of either HIIT or MICT can improve cardiorespiratory fitness and glucose control and lead to reductions in toll-like receptors TLR2 and TLR4 expression whilst MICT, which involved a longer duration of exercise, may be superior for reducing fasting glucose (Robinson et al., 2015). Another trial reported that a relatively short duration of either HIIT or MIT training may improve cardiometabolic risk factors in previously sedentary overweight or obese young men, with no clear advantage between these two specific regimes (Fisher et al., 2015). |
| 123 | Ever told doctor had trouble sleeping? | Question from the NHANES questionnaire: “{Have you/Has SP} ever told a doctor or other health professional that {you have/s/he has} trouble sleeping?” Coded as 1 = no, 2 = yes. | A study found that insomnia with short sleep duration is associated with increased odds of diabetes (Vgontzas et al., 2009). A population-based retrospective cohort study revealed that chronic insomnia could be an important risk factor for T2DM (Lin et al., 2018). A randomized controlled trial deduced that improved sleep quality in older adults with insomnia reduces biomarkers of cardiometabolic disease risk (Carroll et al., 2015). A retrospective cohort study revealed that insomnia is associated with an increased risk of type 2 diabetes in the clinical setting (LeBlanc et al., 2018). |
| 124 | Smoked at least 100 cigarettes in life? | Question from the NHANES questionnaire: “These next questions are about cigarette smoking and other tobacco use. {Have you/Has SP} smoked at least 100 cigarettes in {your/his/her} entire life?” Coded as 1 = no, 2 = yes. | A systematic review and meta-analysis reported that active smoking is associated with an increased risk of type 2 diabetes (Willi et al., 2007). A prospective study found that cigarette smoking may be an independent, modifiable risk factor for non-insulin dependent diabetes mellitus (Rimm et al., 1995). A prospective cohort study revealed that cigarette smoking predicts incident type 2 diabetes, but smoking cessation leads to higher short-term risk. It recommended for smokers at risk for diabetes, smoking cessation should be coupled with strategies for diabetes prevention and early detection (Yeh et al., 2010). A prospective study revealed that Compared with that for never smokers, the risk for diabetes was significantly elevated in current smokers (hazard ratio = 1.28, 95% confidence interval: 1.20, 1.36) but was even higher in women who quit smoking during the first 3 years of follow-up (hazard ratio = 1.43, 95% confidence interval: 1.26, 1.63). Among former smokers, the risk of diabetes decreased significantly as the time since quitting increased and was equal to that of never smokers following a cessation period of 10 years. In new quitters with low cumulative exposure (<20 pack-years), diabetes risk was not elevated following smoking cessation. In conclusion, the risk of diabetes in former smokers returns to that in never smokers 10 years after quitting, and even more quickly in lighter smokers (Luo et al., 2013). A prospective study found that current smokers had an increased risk of diabetes and observed a significant dose-response trend for higher risk among heavier smokers suggesting that cigarette smoking may be an independent, modifiable risk factor for noninsulin-dependent diabetes mellitus (Rimm et al., 1993). A prospective study reported that cigarette smoking is an independent and modifiable risk factor for type 2 diabetes (Wannamethee et al., 2001). As per a cross-sectional study, current and past smoking are associated with a risk of diabetes mellitus essentially in men, but much less in women, and the relationship between fasting glucose and smoking appears different in men and women (Beziaud et al., 2004). A systematic review and meta-analysis revealed that active and passive smoking are associated with significantly increased risks of type 2 diabetes. The risk of diabetes is increased in new quitters, but decreases substantially as the time since quitting increases (Pan et al., 2015). A prospective study provided longitudinal evidence that smoking increases the risk of incident diabetes (Jee et al., 2010). |
| 125 | Self-rated general health | Question from the NHANES questionnaire: “Next I have some general questions about {your/SP's} health. Would you say {your/SP's} health in general is …” Responses were reverse coded as 1 = poor, 2 = fair, 3 = good, 4 = very good, 5 = excellent. Modelled as numeric. | A prospective study showed that low self-rated health was associated with a higher risk of type 2 diabetes, which could be only partly explained by other health-related variables, of which obesity was the strongest. It found no indication of heterogeneity in the association between self-rated health and type 2 diabetes mellitus across the European centers (Wennberg et al., 2013). Fair or poorer SRH was independently and positively associated with the development of T2D in a large-scale cohort study of apparently healthy Korean adults, indicating that SRH is a predictor of metabolic health (Noh et al., 2019). |
| 126 | Minutes of sedentary activity | Question from the NHANES questionnaire: “The following question is about sitting at work, at home, getting to and from places, or with friends, including time spent sitting at a desk, traveling in a car or bus, reading, playing cards, watching television, or using a computer. Do not include time spent sleeping. How much time {do you/does SP} usually spend sitting on a typical day?” | A prospective cohort study revealed that, independent of exercise levels, sedentary behaviors, especially TV watching, were associated with significantly elevated risk of obesity and type 2 diabetes (Hu et al., 2003). Another study reported that, independent of time spent in moderate-to-vigorous–intensity activity, there were significant associations of sedentary time, light-intensity time, and mean activity intensity with waist circumference and clustered metabolic risk (Healy et al., 2008). A systematic review and dose-response meta-analysis reported that, independent of physical activity, total sitting and TV viewing time are associated with greater risk for several major chronic disease outcomes (Patterson et al., 2018). A cross-sectional analysis reported that, in older adults sedentary behaviour is associated with cardiometabolic risk factors, but the associations are more consistent when is measured by self-report that includes TV viewing (Stamatakis et al., 2012). |
| 127 | How much sleep do you get (hours)? | Question from the NHANES questionnaire: “The next set of questions is about your sleeping habits. How much sleep {do you/does SP} usually get at night on weekdays or workdays?” | A prospective study reported that short sleep duration could be a significant risk factor for diabetes. The association between long sleep duration and diabetes incidence is more likely to be due to some unmeasured confounder such as poor sleep quality (Gangwisch et al., 2007). An analysis of NHANES data reported that American short and long sleepers of black race may be at greater risk for diabetes independently of their sociodemographic profile or the presence of comorbid medical conditions, which have been shown to influence habitual sleep durations (Zizi et al., 2012). Another study found that short sleep is an independent risk factor for type 2 diabetes in whites and Hispanics (Beihl et al., 2009). A study revealed that both short and long sleep durations were independently associated with newly diagnosed diabetes (Chao et al., 2011). A longitudinal analysis reported that short sleep duration, but not long duration, was significantly associated with increased diabetes risk (Nuyujukian et al., 2016). |
| **Socio-economic, demographic and associated** | | |  |
| 128 | Age | Age in years of the participant at the time of screening. Individuals 80 and over are top coded at 80 years of age. | A major risk factor of T2D, and included in almost all risk prediction tools of T2D (Lindström & Tuomilehto, 2003; Chien et al., 2009; Chen et al., 2010; De Sousa et al., 2009; Mühlenbruch et al., 2014). |
| 129 | Gender | Gender of the participant. Coded as 1 = female, 2 = male. | Gender and body weight status play a critical role in determining the direction of the association between psychosocial stress and T2DM (Heraclides et al., 2012). A prospective study found that childhood socioeconomic position was a robust predictor of incident diabetes, especially among women. A cumulative risk effect was observed for both childhood socioeconomic position and adult BMI, especially among women (Maty et al., 2008). Gender differences in glucose homeostasis and diabetes are well-documented (Mauvais-Jarvis, 2018). |
| 130 | Race | Reported race and Hispanic origin information. Recoded as 1 = non-Hispanic White, 2 = All other incl. Hispanic etc. | A population-based study reported ethnic disparities in diabetes risk (Brancati et al., 1996). Interactions between race/ethnicity and anthropometry in risk of incident diabetes were found in the Multi-Ethnic Study of Atherosclerosis (Lutsey et al., 2010). |
| 131 | Citizenship | Question from the NHANES questionnaire: “{Are you/Is SP} a citizen of the United States? [Information about citizenship is being collected by the U.S. Public Health Service to perform health related research. Providing this information is voluntary and is collected under the authority of the Public Health Service Act. There will be no effect on pending immigration or citizenship petitions.]” Coded as 1 = not a citizen of the US 2 = citizen by birth or naturalization. | A Canadian study reported that recent immigrants, particularly women and immigrants of South Asian and African origin, are at high risk for diabetes compared with long-term residents of Ontario (Creatore et al., 2010). A study on the association between acculturation and diabetes risk revealed that Among participants born in Mexico, diabetes risk increased with longer duration of US residence (Anderson et al., 2016). A retrospective cohort study reported that refugees and immigrants had significantly increased risk for diabetes, partially mediated by education (Berkowitz et al., 2016). |
| 132 | Marital status | Marital status of participants. Recoded as 1 = All other 2 = married/living with partner | A follow-up study revealed that the relationship between marital status and health outcomes varied by gender. among women, being widowed was associated with a lower risk of T2D (Ramezankhani et al., 2019). A follow-up study revealed that marital status was independently associated with T2DM incidence. Individuals that remained married, despite having significantly increased their weight, were significantly less likely to develop diabetes than their divorced counterparts (de Oliveira et al., 2020). |
| 133 | Education level | (SP Interview Version) What is the highest grade or level of school {you have/SP has} completed or the highest degree {you have/s/he has} received? Coded as 1 = Less than 9th Grade, 2 = 9-11th Grade (Includes 12th grade with no diploma), 3 = High School Grad/GED or equivalent, 4 = Some College or AA degree, 5 = College Graduate or above. Modelled as numeric. | A study reported that there is a considerable burden of type 2 diabetes attributed to lower educational levels in Sweden (Agardh et al., 2011). A case-cohort study demonstrated the inequalities in the risk of T2DM in Western European countries, with an inverse relationship between educational level and risk of T2DM that is only partially explained by variations in BMI (Sacerdote et al., 2012). |
| 134 | Total no: of people in the household | Total number of people in the household; self-reported. Top-coded at 7. | A quasi-experimental study reported that neighborhood deprivation increased the risk of diabetes in refugees in Sweden (White et al., 2016). |
| 135 | Total no: of people in the family | Total number of people in the family; self-reported. Top coded at 7. | A study showed an association between deprivation and type 2 diabetes prevalence (Larranaga et al., 2005). A pooled analysis of five population‐based studies revealed that Regional deprivation plays a significant part in the explanation of diabetes prevalence in Germany independently of individual socio‐economic status (Maier et al., 2013). |
| 136 | Income-poverty ratio | Poverty income ratio (PIR) - a ratio of family income to poverty threshold. Top-coded at 5.00. | A study showed that socio-economic status, assessed with any of three common measures (income, education, occupation), is a risk factor for diagnosed diabetes in women. Among men these associations are less consistent (Robbins et al., 2005). A follow-up study revealed that poverty increases type 2 diabetes incidence and inequality of care despite universal health coverage, in Taiwan (Hsu et al., 2012). |
| 137 | Monthly family income | Monthly family income (reported as a range value in dollars). Modelled as numeric. | A prospective study revealed that chronic inflammation explained a substantial part of the association between life-course socioeconomic disadvantage and type 2 diabetes (Stringhini et al., 2013). A follow-up study revealed that nneighbourhood socioeconomic characteristics in Chicago may be associated with the risk of diabetes in youth (Grigsby-Toussaint et al., 2010). A systematic review revealed that childhood socio-economic status is associated with type 2 diabetes and obesity in later life (Tamayo et al., 2010). An inverse association between socioeconomic status and the prevalence of type 2 diabetes in the middle years of life was reported by a cross-sectional study (Connolly et al., 2000). Findings from a prospective study suggests an important role for life-course SEP measures in determining risk of diabetes, regardless of race and after adjustment for factors that may confound or mediate these associations (Maty et al., 2010). A follow-up study reported that advanced education and increasing income were both inversely associated with incident diabetes even in a relatively well-educated cohort (Lee et al., 2011). A cohort study reported that neighbourhood socioeconomic disadvantage is associated with differences in health risks across the life course, including detrimental lifestyle factors from childhood and adolescence onwards and worse glucose metabolism from early adulthood. By middle age, cumulative neighbourhood socioeconomic disadvantage is associated with increased cardiometabolic risk factors and increased incidence of diabetes (Kivimäki et al., 2018). |
| 138 | Family monthly poverty level index | Family monthly poverty level index, a ratio of monthly family income to the HHS poverty guidelines specific to family size. Top-coded at 5.00. Modelled as numeric. | See no:137 above. |
| 139 | Family monthly poverty level category | Family monthly poverty level index categories. Coded as 1 = Monthly poverty level index <= 1.30, 2 = 1.30 < Monthly poverty level index <= 1.85, 3 = Monthly poverty level index > 1.85. Modelled as numeric. | See no:137 above. |

**REFERENCES**

Stamatakis, E., Davis, M., Stathi, A. and Hamer, M., 2012. Associations between multiple indicators of objectively-measured and self-reported sedentary behaviour and cardiometabolic risk in older adults. *Preventive Medicine*, *54*(1), pp.82-87.

Kivimäki, M., Vahtera, J., Tabák, A.G., Halonen, J.I., Vineis, P., Pentti, J., Pahkala, K., Rovio, S., Viikari, J., Kähönen, M. and Juonala, M., 2018. Neighbourhood socioeconomic disadvantage, risk factors, and diabetes from childhood to middle age in the Young Finns Study: a cohort study. *The Lancet Public Health*, *3*(8), pp.e365-e373.

Lee, T.C., Glynn, R.J., Peña, J.M., Paynter, N.P., Conen, D., Ridker, P.M., Pradhan, A.D., Buring, J.E. and Albert, M.A., 2011. Socioeconomic status and incident type 2 diabetes mellitus: data from the Women's Health Study. *PLoS One*, *6*(12), p.e27670.

Maty, S.C., James, S.A. and Kaplan, G.A., 2010. Life-course socioeconomic position and incidence of diabetes mellitus among blacks and whites: the Alameda County Study, 1965–1999. *American Journal of Public Health*, *100*(1), pp.137-145.

Connolly, V., Unwin, N., Sherriff, P., Bilous, R. and Kelly, W., 2000. Diabetes prevalence and socioeconomic status: a population based study showing increased prevalence of type 2 diabetes mellitus in deprived areas. *Journal of Epidemiology & Community Health*, *54*(3), pp.173-177.

Tamayo, T., Herder, C. and Rathmann, W., 2010. Impact of early psychosocial factors (childhood socioeconomic factors and adversities) on future risk of type 2 diabetes, metabolic disturbances and obesity: a systematic review. *BMC Public Health*, *10*(1), p.525.

Grigsby-Toussaint, D.S., Lipton, R., Chavez, N., Handler, A., Johnson, T.P. and Kubo, J., 2010. Neighborhood socioeconomic change and diabetes risk: findings from the Chicago childhood diabetes registry. *Diabetes Care*, *33*(5), pp.1065-1068.

Stringhini, S., Batty, G.D., Bovet, P., Shipley, M.J., Marmot, M.G., Kumari, M., Tabak, A.G. and Kivimäki, M., 2013. Association of lifecourse socioeconomic status with chronic inflammation and type 2 diabetes risk: the Whitehall II prospective cohort study. *PLoS Med*, *10*(7), p.e1001479.

Hsu, C.C., Lee, C.H., Wahlqvist, M.L., Huang, H.L., Chang, H.Y., Chen, L., Shih, S.F., Shin, S.J., Tsai, W.C., Chen, T. and Huang, C.T., 2012. Poverty increases type 2 diabetes incidence and inequality of care despite universal health coverage. *Diabetes Care*, *35*(11), pp.2286-2292.

Robbins, J.M., Vaccarino, V., Zhang, H. and Kasl, S.V., 2005. Socioeconomic status and diagnosed diabetes incidence. *Diabetes Research and Clinical Practice*, *68*(3), pp.230-236.

Maier, W., Holle, R., Hunger, M., Peters, A., Meisinger, C., Greiser, K.H., Kluttig, A., Völzke, H., Schipf, S., Moebus, S. and Bokhof, B., 2013. The impact of regional deprivation and individual socio‐economic status on the prevalence of Type 2 diabetes in Germany. A pooled analysis of five population‐based studies. *Diabetic Medicine*, *30*(3), pp.e78-e86.

Larranaga, I., Arteagoitia, J.M., Rodriguez, J.L., Gonzalez, F., Esnaola, S., Pinies, J.A. and Sentinel Practice Network of the Basque Country, 2005. Socio‐economic inequalities in the prevalence of Type 2 diabetes, cardiovascular risk factors and chronic diabetic complications in the Basque Country, Spain. *Diabetic Medicine*, *22*(8), pp.1047-1053.

White, J.S., Hamad, R., Li, X., Basu, S., Ohlsson, H., Sundquist, J. and Sundquist, K., 2016. Long-term effects of neighbourhood deprivation on diabetes risk: quasi-experimental evidence from a refugee dispersal policy in Sweden. *The Lancet Diabetes & Endocrinology*, *4*(6), pp.517-524.

Sacerdote, C., Ricceri, F., Rolandsson, O., Baldi, I., Chirlaque, M.D., Feskens, E., Bendinelli, B., Ardanaz, E., Arriola, L., Balkau, B. and Bergmann, M., 2012. Lower educational level is a predictor of incident type 2 diabetes in European countries: the EPIC-InterAct study. *International Journal of Epidemiology*, *41*(4), pp.1162-1173.

Agardh, E.E., Sidorchuk, A., Hallqvist, J., Ljung, R., Peterson, S., Moradi, T. and Allebeck, P., 2011. Burden of type 2 diabetes attributed to lower educational levels in Sweden. *Population Health Metrics*, *9*(1), p.60.

de Oliveira, C.M., Viater Tureck, L., Alvares, D., Liu, C., Horimoto, A.R.V.R., Balcells, M., de Oliveira Alvim, R., Krieger, J.E. and Pereira, A.C., 2020. Relationship between marital status and incidence of type 2 diabetes mellitus in a Brazilian rural population: The Baependi Heart Study. *PLoS One*, *15*(8), p.e0236869.

Ramezankhani, A., Azizi, F. and Hadaegh, F., 2019. Associations of marital status with diabetes, hypertension, cardiovascular disease and all-cause mortality: a long term follow-up study. *PLoS One*, *14*(4), p.e0215593.

Berkowitz, S.A., Fabreau, G.E., Raghavan, S., Kentoffio, K., Chang, Y., He, W., Atlas, S.J. and Percac-Lima, S., 2016. Risk of developing diabetes among refugees and immigrants: a longitudinal analysis. *Journal of Community Health*, *41*(6), pp.1274-1281.

Anderson, C., Zhao, H., Daniel, C.R., Hromi-Fiedler, A., Dong, Q., Elhor Gbito, K.Y., Wu, X. and Chow, W.H., 2016. Acculturation and diabetes risk in the Mexican American Mano a Mano Cohort. *American Journal of Public Health*, *106*(3), pp.547-549.

Creatore, M.I., Moineddin, R., Booth, G., Manuel, D.H., DesMeules, M., McDermott, S. and Glazier, R.H., 2010. Age-and sex-related prevalence of diabetes mellitus among immigrants to Ontario, Canada. *CMAJ*, *182*(8), pp.781-789.

Lutsey, P.L., Pereira, M.A., Bertoni, A.G., Kandula, N.R. and Jacobs Jr, D.R., 2010. Interactions between race/ethnicity and anthropometry in risk of incident diabetes: the multi-ethnic study of atherosclerosis. *American Journal of Epidemiology*, *172*(2), pp.197-204.

Brancati, F.L., Whelton, P.K., Kuller, L.H. and Klag, M.J., 1996. Diabetes mellitus, race, and socioeconomic status a population-based study. *Annals of Epidemiology*, *6*(1), pp.67-73.

Mauvais-Jarvis, F., 2018. Gender differences in glucose homeostasis and diabetes. *Physiology & Behavior*, *187*, pp.20-23.

Maty, S.C., Lynch, J.W., Raghunathan, T.E. and Kaplan, G.A., 2008. Childhood socioeconomic position, gender, adult body mass index, and incidence of type 2 diabetes mellitus over 34 years in the Alameda County Study. *American Journal of Public Health*, *98*(8), pp.1486-1494.

Heraclides, A.M., Chandola, T., Witte, D.R. and Brunner, E.J., 2012. Work stress, obesity and the risk of Type 2 Diabetes: Gender‐specific bidirectional effect in the Whitehall II study. *Obesity*, *20*(2), pp.428-433.

Mühlenbruch, K., Joost, H.G., Boeing, H. and Schulze, M.B., 2014. Risk prediction for type 2 diabetes in the German population with the updated German Diabetes Risk Score (GDRS). *Ernährungs Umschau*, *61*(6), pp.90-93.

De Sousa, A.G.P., Pereira, A.C., Marquezine, G.F., do Nascimento-Neto, R.M., Freitas, S.N., Nicolato, R.L.D.C., Machado-Coelho, G.L.L., Rodrigues, S.L., Mill, J.G. and Krieger, J.E., 2009. Derivation and external validation of a simple prediction model for the diagnosis of type 2 diabetes mellitus in the Brazilian urban population. *European Journal of Epidemiology*, *24*(2), p.101.

Chen, L., Magliano, D.J., Balkau, B., Colagiuri, S., Zimmet, P.Z., Tonkin, A.M., Mitchell, P., Phillips, P.J. and Shaw, J.E., 2010. AUSDRISK: an Australian Type 2 Diabetes Risk Assessment Tool based on demographic, lifestyle and simple anthropometric measures. *Medical Journal of Australia*, *192*(4), pp.197-202.

Chien, K., Cai, T., Hsu, H., Su, T., Chang, W., Chen, M., Lee, Y. and Hu, F.B., 2009. A prediction model for type 2 diabetes risk among Chinese people. *Diabetologia*, *52*(3), p.443.

Lindström, J. and Tuomilehto, J., 2003. The diabetes risk score: a practical tool to predict type 2 diabetes risk. *Diabetes Care*, *26*(3), pp.725-731.

Nuyujukian, D.S., Beals, J., Huang, H., Johnson, A., Bullock, A., Manson, S.M. and Jiang, L., 2016. Sleep duration and diabetes risk in American Indian and Alaska native participants of a lifestyle intervention project. *Sleep*, *39*(11), pp.1919-1926.

Chao, C.Y., Wu, J.S., Yang, Y.C., Shih, C.C., Wang, R.H., Lu, F.H. and Chang, C.J., 2011. Sleep duration is a potential risk factor for newly diagnosed type 2 diabetes mellitus. *Metabolism*, *60*(6), pp.799-804.

Beihl, D.A., Liese, A.D. and Haffner, S.M., 2009. Sleep duration as a risk factor for incident type 2 diabetes in a multiethnic cohort. *Annals of Epidemiology*, *19*(5), pp.351-357.

Zizi, F., Pandey, A., Murrray-Bachmann, R., Vincent, M., McFarlane, S., Ogedegbe, G. and Jean-Louis, G., 2012. Race/ethnicity, sleep duration, and diabetes mellitus: analysis of the National Health Interview Survey. *The American Journal of Medicine*, *125*(2), pp.162-167.

Gangwisch, J.E., Heymsfield, S.B., Boden-Albala, B., Buijs, R.M., Kreier, F., Pickering, T.G., Rundle, A.G., Zammit, G.K. and Malaspina, D., 2007. Sleep duration as a risk factor for diabetes incidence in a large US sample. *Sleep*, *30*(12), pp.1667-1673.

Najibi, N., Firoozi, R., Shahrezaee, S., Eshraghian, M., Daneshi-Maskooni, M. and Dorosty-Motlagh, A., 2019. Food insecurity is an important risk factor for type 2 diabetes: a case-control study of new referrals to the University clinics, Shiraz, Southern Iran. *BMC Public Health*, *19*(1), p.885.

Seligman, H.K., Bindman, A.B., Vittinghoff, E., Kanaya, A.M. and Kushel, M.B., 2007. Food insecurity is associated with diabetes mellitus: results from the National Health Examination and Nutrition Examination Survey (NHANES) 1999–2002. *Journal of General Internal Medicine*, *22*(7), pp.1018-1023.

Green, A.J., Bazata, D.D., Fox, K.M., Grandy, S. and SHIELD Study Group, 2007. Health‐related behaviours of people with diabetes and those with cardiometabolic risk factors: results from SHIELD. *International Journal of Clinical Practice*, *61*(11), pp.1791-1797.

Mogre, V., Abedandi, R. and Salifu, Z.S., 2014. Distorted self-perceived weight status and underestimation of weight status in diabetes mellitus type 2 patients. *PLoS One*, *9*(4), p.e95165.

Hamman, R.F., Wing, R.R., Edelstein, S.L., Lachin, J.M., Bray, G.A., Delahanty, L., Hoskin, M., Kriska, A.M., Mayer-Davis, E.J., Pi-Sunyer, X. and Regensteiner, J., 2006. Effect of weight loss with lifestyle intervention on risk of diabetes. *Diabetes Care*, *29*(9), pp.2102-2107.

Radzeviciene, L. and Ostrauskas, R., 2017. Adding salt to meals as a risk factor of type 2 diabetes mellitus: a case–control study. *Nutrients*, *9*(1), p.67.

Bolla, A.M., Caretto, A., Laurenzi, A., Scavini, M. and Piemonti, L., 2019. Low-carb and ketogenic diets in type 1 and type 2 diabetes. *Nutrients*, *11*(5), p.962.

Salas-Salvadó, J., Guasch-Ferré, M., Lee, C.H., Estruch, R., Clish, C.B. and Ros, E., 2015. Protective effects of the Mediterranean diet on type 2 diabetes and metabolic syndrome. *The Journal of Nutrition*, *146*(4), pp.920S-927S.

Kanerva, N., Rissanen, H., Knekt, P., Havulinna, A.S., Eriksson, J.G. and Männistö, S., 2014. The healthy Nordic diet and incidence of type 2 diabetes—10-year follow-up. *Diabetes Research and Clinical Practice*, *106*(2), pp.e34-e37.

Liese, A.D., Weis, K.E., Schulz, M. and Tooze, J.A., 2009. Food intake patterns associated with incident type 2 diabetes: the Insulin Resistance Atherosclerosis Study. *Diabetes Care*, *32*(2), pp.263-268.

Mekary, R.A., Giovannucci, E., Willett, W.C., van Dam, R.M. and Hu, F.B., 2012. Eating patterns and type 2 diabetes risk in men: breakfast omission, eating frequency, and snacking. *The American Journal of Clinical Nutrition*, *95*(5), pp.1182-1189.

Roussel, R., Fezeu, L., Bouby, N., Balkau, B., Lantieri, O., Alhenc-Gelas, F., Marre, M., Bankir, L. and DESIR Study Group, 2011. Low water intake and risk for new-onset hyperglycemia. *Diabetes Care*, *34*(12), pp.2551-2554.

Stene, L.C., Hongve, D., Magnus, P., Rønningen, K.S. and Joner, G., 2002. Acidic drinking water and risk of childhood-onset type 1 diabetes. *Diabetes Care*, *25*(9), pp.1534-1538.

Healy, G.N., Wijndaele, K., Dunstan, D.W., Shaw, J.E., Salmon, J., Zimmet, P.Z. and Owen, N., 2008. Objectively measured sedentary time, physical activity, and metabolic risk: the Australian Diabetes, Obesity and Lifestyle Study (AusDiab). *Diabetes Care*, *31*(2), pp.369-371.

Patel, P.S., Sharp, S.J., Luben, R.N., Khaw, K.T., Bingham, S.A., Wareham, N.J. and Forouhi, N.G., 2009. Association between type of dietary fish and seafood intake and the risk of incident type 2 diabetes: the European prospective investigation of cancer (EPIC)-Norfolk cohort study. *Diabetes Care*, *32*(10), pp.1857-1863.

Rylander, C., Sandanger, T.M., Engeset, D. and Lund, E., 2014. Consumption of lean fish reduces the risk of type 2 diabetes mellitus: a prospective population based cohort study of Norwegian women. *PLoS One*, *9*(2), p.e89845.

Wallin, A., Di Giuseppe, D., Orsini, N., Åkesson, A., Forouhi, N.G. and Wolk, A., 2017. Fish consumption and frying of fish in relation to type 2 diabetes incidence: a prospective cohort study of Swedish men. *European Journal of Nutrition*, *56*(2), pp.843-852.

Yeung, S., Soliternik, J., and Mazzola, N., 2018. Nutritional supplements for the prevention of diabetes mellitus and its complications. *Journal of Nutrition & Intermediary Metabolism*, *14*, pp.16-21.

Yu, T.Y., Wei, J.N., Kuo, C.H., Liou, J.M., Lin, M.S., Shih, S.R., Hua, C.H., Hsein, Y.C., Hsu, Y.W., Chuang, L.M. and Lee, M.K., 2017. The impact of gastric atrophy on the incidence of diabetes. *Scientific Reports*, *7*(1), pp.1-8.

Nettleton, J.A., Steffen, L.M., Ni, H., Liu, K. and Jacobs, D.R., 2008. Dietary patterns and risk of incident type 2 diabetes in the Multi-Ethnic Study of Atherosclerosis (MESA). *Diabetes Care*, *31*(9), pp.1777-1782.

Anekwe, T.D. and Rahkovsky, I., 2018. The association between food prices and the blood glucose level of US adults with type 2 diabetes. *American Journal of Public Health*, *108*(S6), pp.S475-S482.

Gucciardi, E., Vahabi, M., Norris, N., Del Monte, J.P. and Farnum, C., 2014. The intersection between food insecurity and diabetes: a review. *Current Nutrition Reports*, *3*(4), pp.324-332.

Kern, D.M., Auchincloss, A.H., Stehr, M.F., Roux, A.V.D., Moore, K.A., Kanter, G.P. and Robinson, L.F., 2018. Neighborhood price of healthier food relative to unhealthy food and its association with type 2 diabetes and insulin resistance: The multi-ethnic study of atherosclerosis. *Preventive Medicine*, *106*, pp.122-129.

Tait, C.A., L’Abbé, M.R., Smith, P.M. and Rosella, L.C., 2018. The association between food insecurity and incident type 2 diabetes in Canada: A population-based cohort study. *PLoS One*, *13*(5), p.e0195962.

Zong, G., Eisenberg, D.M., Hu, F.B. and Sun, Q., 2016. Consumption of meals prepared at home and risk of type 2 diabetes: an analysis of two prospective cohort studies. *PLoS Medicine*, *13*(7), p.e1002052.

Sarkar, C., Webster, C. and Gallacher, J., 2018. Are exposures to ready-to-eat food environments associated with type 2 diabetes? A cross-sectional study of 347 551 UK Biobank adult participants. *The Lancet Planetary Health*, *2*(10), pp.e438-e450.

Kudo, A., Asahi, K., Satoh, H., Iseki, K., Moriyama, T., Yamagata, K., Tsuruya, K., Fujimoto, S., Narita, I., Konta, T. and Kondo, M., 2019. Fast eating is a strong risk factor for new-onset diabetes among the Japanese general population. *Scientific Reports*, *9*(1), pp.1-8.

Yu, D., Zheng, W., Cai, H., Xiang, Y.B., Li, H., Gao, Y.T. and Shu, X.O., 2018. Long-term diet quality and risk of type 2 diabetes among urban Chinese adults. *Diabetes Care*, *41*(4), pp.723-730.

Ley, S.H., Pan, A., Li, Y., Manson, J.E., Willett, W.C., Sun, Q. and Hu, F.B., 2016. Changes in overall diet quality and subsequent type 2 diabetes risk: three US prospective cohorts. *Diabetes Care*, *39*(11), pp.2011-2018.

Liu, S., Choi, H.K., Ford, E., Song, Y., Klevak, A., Buring, J.E. and Manson, J.E., 2006. A prospective study of dairy intake and the risk of type 2 diabetes in women. *Diabetes Care*, *29*(7), pp.1579-1584.

Drouin-Chartier, J.P., Li, Y., Ardisson Korat, A.V., Ding, M., Lamarche, B., Manson, J.E., Rimm, E.B., Willett, W.C. and Hu, F.B., 2019. Changes in dairy product consumption and risk of type 2 diabetes: results from 3 large prospective cohorts of US men and women. *The American Journal of Clinical Nutrition*, *110*(5), pp.1201-1212.

Gao, D., Ning, N., Wang, C., Wang, Y., Li, Q., Meng, Z., Liu, Y. and Li, Q., 2013. Dairy products consumption and risk of type 2 diabetes: systematic review and dose-response meta-analysis. *PLoS One*, *8*(9), p.e73965.

Chen, M., Sun, Q., Giovannucci, E., Mozaffarian, D., Manson, J.E., Willett, W.C. and Hu, F.B., 2014. Dairy consumption and risk of type 2 diabetes: 3 cohorts of US adults and an updated meta-analysis. *BMC Medicine*, *12*(1), p.215.

Wang, B., Liu, L., Qiao, D., Xue, Y., Liu, X., Zhang, D., Liu, C., Mao, Z., Yu, S., Shen, F. and Zhang, Y., 2020. The association between frequency of away-from home meals and type 2 diabetes mellitus in rural Chinese adults: the Henan Rural Cohort Study. *European Journal of Nutrition*, pp.1-11.

Pereira, M.A., Kartashov, A.I., Ebbeling, C.B., Van Horn, L., Slattery, M.L., Jacobs Jr, D.R. and Ludwig, D.S., 2005. Fast-food habits, weight gain, and insulin resistance (the CARDIA study): 15-year prospective analysis. *The Lancet*, *365*(9453), pp.36-42.

Bodicoat, D.H., Carter, P., Comber, A., Edwardson, C., Gray, L.J., Hill, S., Webb, D., Yates, T., Davies, M.J. and Khunti, K., 2015. Is the number of fast-food outlets in the neighbourhood related to screen-detected type 2 diabetes mellitus and associated risk factors?. *Public Health Nutrition*, *18*(9), pp.1698-1705.

Fitzgerald, N., Hromi-Fiedler, A., Segura-Pérez, S. and Pérez-Escamilla, R., 2011. Food insecurity is related to increased risk of type 2 diabetes among Latinas. *Ethnicity & Disease*, *21*(3), p.328.

Seligman, H.K., Bindman, A.B., Vittinghoff, E., Kanaya, A.M. and Kushel, M.B., 2007. Food insecurity is associated with diabetes mellitus: results from the National Health Examination and Nutrition Examination Survey (NHANES) 1999–2002. *Journal of General Internal Medicine*, *22*(7), pp.1018-1023.

Colditz, G.A., Willett, W.C., Stampfer, M.J., Manson, J.E., Hennekens, C.H., Arky, R.A. and Speizer, F.E., 1990. Weight as a risk factor for clinical diabetes in women. *American Journal of Epidemiology*, *132*(3), pp.501-513.

Colditz, G.A., Willett, W.C., Rotnitzky, A. and Manson, J.E., 1995. Weight gain as a risk factor for clinical diabetes mellitus in women. *Annals of Internal Medicine*, *122*(7), pp.481-486.

Harder, T., Rodekamp, E., Schellong, K., Dudenhausen, J.W. and Plagemann, A., 2007. Birth weight and subsequent risk of type 2 diabetes: a meta-analysis. *American Journal of Epidemiology*, *165*(8), pp.849-857.

Ganz, M.L., Wintfeld, N., Li, Q., Alas, V., Langer, J. and Hammer, M., 2014. The association of body mass index with the risk of type 2 diabetes: a case–control study nested in an electronic health records system in the United States. *Diabetology & Metabolic Syndrome*, *6*(1), p.50.

Wittenbecher, C., Kuxhaus, O., Boeing, H., Stefan, N. and Schulze, M.B., 2019. Associations of short stature and components of height with incidence of type 2 diabetes: mediating effects of cardiometabolic risk factors. *Diabetologia*, *62*(12), pp.2211-2221.

Asao, K., Kao, W.L., Baptiste-Roberts, K., Bandeen-Roche, K., Erlinger, T.P. and Brancati, F.L., 2006. Short stature and the risk of adiposity, insulin resistance, and type 2 diabetes in middle age: the Third National Health and Nutrition Examination Survey (NHANES III), 1988–1994. *Diabetes Care*, *29*(7), pp.1632-1637.

Schienkiewitz, A., Schulze, M.B., Hoffmann, K., Kroke, A. and Boeing, H., 2006. Body mass index history and risk of type 2 diabetes: results from the European Prospective Investigation into Cancer and Nutrition (EPIC)–Potsdam Study–. *The American Journal of Clinical Nutrition*, *84*(2), pp.427-433.

Weitzman, S., Wang, C.H., Pankow, J.S., Schmidt, M.I. and Brancati, F.L., 2010. Are measures of height and leg length related to incident diabetes mellitus? The ARIC (Atherosclerosis Risk in Communities) study. *Acta Diabetologica*, *47*(3), pp.237-242.

Smith, G.D., Greenwood, R., Gunnell, D., Sweetnam, P., Yarnell, J. and Elwood, P., 2001. Leg length, insulin resistance, and coronary heart disease risk: the Caerphilly Study. *Journal of Epidemiology & Community Health*, *55*(12), pp.867-872.

Johnston, L.W., Harris, S.B., Retnakaran, R., Gerstein, H.C., Zinman, B., Hamilton, J. and Hanley, A.J., 2013. Short leg length, a marker of early childhood deprivation, is associated with metabolic disorders underlying type 2 diabetes: the PROMISE cohort study. *Diabetes Care*, *36*(11), pp.3599-3606.

Smits, M.M., Boyko, E.J., Utzschneider, K.M., Leonetti, D.L., McNeely, M.J., Suvag, S., Wright, L.A., Fujimoto, W.Y. and Kahn, S.E., 2012. Arm length is associated with type 2 diabetes mellitus in Japanese-Americans. *Diabetologia*, *55*(6), pp.1679-1684.

Hou, Y., Jia, X., Xuan, L., Zhu, W., Deng, C., Wang, L., Zhao, Z., Li, M., Lu, J., Xu, Y. and Chen, Y., 2019. Association between mid-upper arm circumference and cardiometabolic risk in Chinese population: a cross-sectional study. *BMJ Open*, *9*(9), p.e028904.

Zhu, Y., Lin, Q., Zhang, Y., Deng, H., Hu, X., Yang, X. and Yao, B., 2020. Mid-upper arm circumference as a simple tool for identifying central obesity and insulin resistance in type 2 diabetes. *PLoS One*, *15*(5), p.e0231308.

Janiszewski, P.M., Janssen, I. and Ross, R., 2007. Does waist circumference predict diabetes and cardiovascular disease beyond commonly evaluated cardiometabolic risk factors?. *Diabetes Care*, *30*(12), pp.3105-3109.

Vazquez, G., Duval, S., Jacobs Jr, D.R. and Silventoinen, K., 2007. Comparison of body mass index, waist circumference, and waist/hip ratio in predicting incident diabetes: a meta-analysis. *Epidemiologic Reviews*, *29*(1), pp.115-128.

Kim, E.S., Jeong, J.S., Han, K., Kim, M.K., Lee, S.H., Park, Y.M., Baek, K.H., Moon, S.D., Han, J.H., Song, K.H. and Kwon, H.S., 2018. Impact of weight changes on the incidence of diabetes mellitus: a Korean nationwide cohort study. *Scientific Reports*, *8*(1), pp.1-7.

Oguma, Y., Sesso, H.D., Paffenbarger Jr, R.S. and Lee, I.M., 2005. Weight change and risk of developing type 2 diabetes. *Obesity Research*, *13*(5), pp.945-951.

Resnick, H.E., Valsania, P., Halter, J.B. and Lin, X., 2000. Relation of weight gain and weight loss on subsequent diabetes risk in overweight adults. *Journal of Epidemiology & Community Health*, *54*(8), pp.596-602.

Morimoto, Y., Schembre, S.M., Steinbrecher, A., Erber, E., Pagano, I., Grandinetti, A., Kolonel, L.N. and Maskarinec, G., 2011. Ethnic differences in weight gain and diabetes risk: the Multiethnic Cohort Study. *Diabetes & Metabolism*, *37*(3), pp.230-236.

Danquah, I., Galbete, C., Meeks, K., Nicolaou, M., Klipstein-Grobusch, K., Addo, J., Aikins, A.D.G., Amoah, S.K., Agyei-Baffour, P., Boateng, D. and Bedu-Addo, G., 2018. Food variety, dietary diversity, and type 2 diabetes in a multi-center cross-sectional study among Ghanaian migrants in Europe and their compatriots in Ghana: the RODAM study. *European Journal of Nutrition*, *57*(8), pp.2723-2733.

Conklin, A.I., Monsivais, P., Khaw, K.T., Wareham, N.J. and Forouhi, N.G., 2016. Dietary diversity, diet cost, and incidence of type 2 diabetes in the United Kingdom: a prospective cohort study. *PLoS Medicine*, *13*(7), p.e1002085.

Donin, A.S., Nightingale, C.M., Owen, C.G., Rudnicka, A.R., Jebb, S.A., Ambrosini, G.L., Stephen, A.M., Cook, D.G. and Whincup, P.H., 2014. Dietary energy intake is associated with type 2 diabetes risk markers in children. *Diabetes Care*, *37*(1), pp.116-123.

Tinker, L.F., Sarto, G.E., Howard, B.V., Huang, Y., Neuhouser, M.L., Mossavar-Rahmani, Y., Beasley, J.M., Margolis, K.L., Eaton, C.B., Phillips, L.S. and Prentice, R.L., 2011. Biomarker-calibrated dietary energy and protein intake associations with diabetes risk among postmenopausal women from the Women's Health Initiative. *The American Journal of Clinical Nutrition*, *94*(6), pp.1600-1606.

Qi, L., Kraft, P., Hunter, D.J. and Hu, F.B., 2008. The common obesity variant near MC4R gene is associated with higher intakes of total energy and dietary fat, weight change and diabetes risk in women. *Human Molecular Genetics*, *17*(22), pp.3502-3508.

Wang, J., Luben, R., Khaw, K.T., Bingham, S., Wareham, N.J. and Forouhi, N.G., 2008. Dietary energy density predicts the risk of incident type 2 diabetes: the European Prospective Investigation of Cancer (EPIC)-Norfolk Study. *Diabetes Care*, *31*(11), pp.2120-2125.

Sluijs, I., Beulens, J.W., Spijkerman, A.M., Grobbee, D.E. and van der Schouw, Y.T., 2010. Dietary intake of total, animal, and vegetable protein and risk of type 2 diabetes in the European Prospective Investigation into Cancer and Nutrition (EPIC)-NL study. *Diabetes Care*, *33*(1), pp.43-48.

Wang, E.T., de Koning, L. and Kanaya, A.M., 2010. Higher protein intake is associated with diabetes risk in South Asian Indians: the Metabolic Syndrome and Atherosclerosis in South Asians Living in America (MASALA) study. *Journal of the American College of Nutrition*, *29*(2), pp.130-135.

Zhao, L.G., Zhang, Q.L., Liu, X.L., Wu, H., Zheng, J.L. and Xiang, Y.B., 2019. Dietary protein intake and risk of type 2 diabetes: a dose–response meta-analysis of prospective studies. *European Journal of Nutrition*, *58*(4), pp.1351-1367.

Schulze, M.B., Schulz, M., Heidemann, C., Schienkiewitz, A., Hoffmann, K. and Boeing, H., 2008. Carbohydrate intake and incidence of type 2 diabetes in the European Prospective Investigation into Cancer and Nutrition (EPIC)-Potsdam Study. *British Journal of Nutrition*, *99*(5), pp.1107-1116.

Greenwood, D.C., Threapleton, D.E., Evans, C.E., Cleghorn, C.L., Nykjaer, C., Woodhead, C. and Burley, V.J., 2013. Glycemic index, glycemic load, carbohydrates, and type 2 diabetes: systematic review and dose–response meta-analysis of prospective studies. *Diabetes Care*, *36*(12), pp.4166-4171.

Sonestedt, E., Øverby, N., Laaksonen, D. and Eva Birgisdottir, B., 2012. Does high sugar consumption exacerbate cardiometabolic risk factors and increase the risk of type 2 diabetes and cardiovascular disease?. *Food & Nutrition Research*, *56*(1), p.19104.

Seo, E.H., Kim, H. and Kwon, O., 2019. Association between total sugar intake and metabolic syndrome in middle-aged Korean men and women. *Nutrients*, *11*(9), p.2042.

Yao, B., Fang, H., Xu, W., Yan, Y., Xu, H., Liu, Y., Mo, M., Zhang, H. and Zhao, Y., 2014. Dietary fiber intake and risk of type 2 diabetes: a dose–response analysis of prospective studies. *European Journal of Epidemiology*, 29, 79–88. <https://doi.org/10.1007/s10654-013-9876-x>

Lindström, J., Peltonen, M., Eriksson, J.G., Louheranta, A., Fogelholm, M., Uusitupa, M. and Tuomilehto, J., 2006. High-fibre, low-fat diet predicts long-term weight loss and decreased type 2 diabetes risk: the Finnish Diabetes Prevention Study. *Diabetologia*, *49*(5), pp.912-920.

Van Dam, R.M., Willett, W.C., Rimm, E.B., Stampfer, M.J. and Hu, F.B., 2002. Dietary fat and meat intake in relation to risk of type 2 diabetes in men. *Diabetes Care*, *25*(3), pp.417-424.

ARIC Study Investigators, 2003. Plasma fatty acid composition and incidence of diabetes in middle-aged adults: the Atherosclerosis Risk in Communities (ARIC) Study, *The American Journal of Clinical Nutrition*, 78(1), pp. 91–98. <https://doi.org/10.1093/ajcn/78.1.91>

Brunerova, L., Smejkalova, V., Potockova, J. and Andel, M., 2007. A comparison of the influence of a high‐fat diet enriched in monounsaturated fatty acids and conventional diet on weight loss and metabolic parameters in obese non‐diabetic and Type 2 diabetic patients. *Diabetic Medicine*, *24*(5), pp.533-540.

Lankinen, M.A., Stančáková, A., Uusitupa, M., Ågren, J., Pihlajamäki, J., Kuusisto, J., Schwab, U. and Laakso, M., 2015. Plasma fatty acids as predictors of glycaemia and type 2 diabetes. *Diabetologia*, *58*(11), pp.2533-2544.

Virtanen, J.K., Mursu, J., Voutilainen, S., Uusitupa, M. and Tuomainen, T.P., 2014. Serum omega-3 polyunsaturated fatty acids and risk of incident type 2 diabetes in men: the Kuopio Ischemic Heart Disease Risk Factor study. *Diabetes Care*, *37*(1), pp.189-196.

Simonen, P.P., Gylling, H.K. and Miettinen, T.A., 2002. Diabetes contributes to cholesterol metabolism regardless of obesity. *Diabetes Care*, *25*(9), pp.1511-1515.

Meyer, K.A., Kushi, L.H., Jacobs, D.R. and Folsom, A.R., 2001. Dietary fat and incidence of type 2 diabetes in older Iowa women. *Diabetes Care*, *24*(9), pp.1528-1535.

Salonen, J.T., Nyyssonen, K., Tuomainen, T.P., Maenpaa, P.H., Korpela, H., Kaplan, G.A., Lynch, J., Helmrich, S.P. and Salonen, R., 1995. Increased risk of non-insulin dependent diabetes mellitus at low plasma vitamin E concentrations: a four year follow up study in men. *BMJ*, *311*(7013), pp.1124-1127.

Montonen, J., Knekt, P., Järvinen, R. and Reunanen, A., 2004. Dietary antioxidant intake and risk of type 2 diabetes. *Diabetes Care*, *27*(2), pp.362-366.

Mayer-Davis, E.J., Costacou, T., King, I., Zaccaro, D.J. and Bell, R.A., 2002. Plasma and dietary vitamin E in relation to incidence of type 2 diabetes: The Insulin Resistance and Atherosclerosis Study (IRAS). *Diabetes Care*, *25*(12), pp.2172-2177.

Meerza, D., Iqbal, S., Zaheer, S. and Naseem, I., 2016. Retinoids have therapeutic action in type 2 diabetes. *Nutrition*, *32*(7-8), pp.898-903.

Iqbal, S. and Naseem, I., 2015. Role of vitamin A in type 2 diabetes mellitus biology: effects of intervention therapy in a deficient state. *Nutrition*, *31*(7-8), pp.901-907.

Trasino, S.E. and Gudas, L.J., 2015. Vitamin A: a missing link in diabetes?. *Diabetes Management (London, England)*, *5*(5), pp.359–367.

Sluijs, I., Cadier, E., Beulens, J.W.J., Spijkerman, A.M.W. and van der Schouw, Y.T., 2015. Dietary intake of carotenoids and risk of type 2 diabetes. *Nutrition, Metabolism and Cardiovascular Diseases*, *25*(4), pp.376-381.

Ylönen, K., Alfthan, G., Groop, L., Saloranta, C., Aro, A., Virtanen, S.M. and Botnia Research Group, 2003. Dietary intakes and plasma concentrations of carotenoids and tocopherols in relation to glucose metabolism in subjects at high risk of type 2 diabetes: the Botnia Dietary Study. *The American Journal of Clinical Nutrition*, *77*(6), pp.1434-1441.

Sugiura, M., Nakamura, M., Ogawa, K., Ikoma, Y. and Yano, M., 2015. High-serum carotenoids associated with lower risk for developing type 2 diabetes among Japanese subjects: Mikkabi cohort study. *BMJ Open Diabetes Research and Care*, *3*(1).

Sahin, K., Orhan, C., Akdemir, F., Tuzcu, M., Sahin, N., Yılmaz, I. and Juturu, V., 2017. β-Cryptoxanthin ameliorates metabolic risk factors by regulating NF-κB and Nrf2 pathways in insulin resistance induced by high-fat diet in rodents. *Food and Chemical Toxicology*, *107*, pp.270-279.

Iwata, A., Matsubara, S. and Miyazaki, K., 2018. Beneficial effects of a beta-cryptoxanthin-containing beverage on body mass index and visceral fat in pre-obese men: Double-blind, placebo-controlled parallel trials. *Journal of Functional Foods*, *41*, pp.250-257.

Olmedilla, B., Granado, F., Gil-Martinez, E., Blanco, I. and Rojas-Hidalgo, E., 1997. Reference values for retinol, tocopherol, and main carotenoids in serum of control and insulin-dependent diabetic Spanish subjects. *Clinical Chemistry*, *43*(6), pp.1066-1071.

Sugiura, M., 2015. β-cryptoxanthin and the risk for lifestyle-related disease: findings from recent nutritional epidemiologic studies. *Yakugaku zasshi: Journal of the Pharmaceutical Society of Japan*, *135*(1), p.67.

Ford, E.S., Will, J.C., Bowman, B.A. and Narayan, K.V., 1999. Diabetes mellitus and serum carotenoids: findings from the Third National Health and Nutrition Examination Survey. *American Journal of Epidemiology*, *149*(2), pp.168-176.

Nix, W.A., Zirwes, R., Bangert, V., Kaiser, R.P., Schilling, M., Hostalek, U. and Obeid, R., 2015. Vitamin B status in patients with type 2 diabetes mellitus with and without incipient nephropathy. *Diabetes Research and Clinical Practice*, *107*(1), pp.157-165.

Page, G.L.J., Laight, D. and Cummings, M.H., 2011. Thiamine deficiency in diabetes mellitus and the impact of thiamine replacement on glucose metabolism and vascular disease. *International Journal of Clinical Practice*, *65*(6), pp.684-690.

Shin, W.Y. and Kim, J.H., 2019. Low riboflavin intake is associated with cardiometabolic risks in Korean women. *Asia Pacific Journal of Clinical Nutrition*, *28*(2), p.285.

Alam, M.M., Iqbal, S. and Naseem, I., 2015. Ameliorative effect of riboflavin on hyperglycemia, oxidative stress and DNA damage in type-2 diabetic mice: Mechanistic and therapeutic strategies. *Archives of Biochemistry and Biophysics*, *584*, pp.10-19.

Goldie, C., Taylor, A.J., Nguyen, P., McCoy, C., Zhao, X.Q. and Preiss, D., 2016. Niacin therapy and the risk of new-onset diabetes: a meta-analysis of randomised controlled trials. *Heart*, *102*(3), pp.198-203.

Wu, Y., Li, S., Wang, W. and Zhang, D., 2020. Associations of dietary vitamin B1, vitamin B2, niacin, vitamin B6, vitamin B12 and folate equivalent intakes with metabolic syndrome. *International Journal of Food Sciences and Nutrition*, pp.1-12.

Mascolo, E. and Vernì, F., 2020. Vitamin B6 and Diabetes: Relationship and Molecular Mechanisms. *International Journal of Molecular Sciences*, *21*(10), p.3669.

Braun, K.V., Satija, A., Voortman, T., Franco, O., Sun, Q., Hu, F. and Bhupathiraju, S., 2019. Methyl Donor Nutrient Intake and Risk of Type 2 Diabetes: Results from 3 Large US Cohorts (OR15-02-19). *Current Developments in Nutrition*, *3*(Supplement_1), pp.nzz044-OR15.

Zhu, J., Chen, C., Lu, L., Yang, K., Reis, J. and He, K., 2020. Intakes of Folate, Vitamin B6, and Vitamin B12 in Relation to Diabetes Incidence Among American Young Adults: A 30-Year Follow-up Study. *Diabetes Care*. <https://doi.org/10.2337/dc20-0828>

Hong, S.M., Woo, H.W., Kim, M.K., Kim, S.Y., Lee, Y.H., Shin, D.H., Shin, M.H., Chun, B.Y. and Choi, B.Y., 2017. A prospective association between dietary folate intake and type 2 diabetes risk among Korean adults aged 40 years or older: the Korean Multi-Rural Communities Cohort (MRCohort) Study. *British Journal of Nutrition*, *118*(12), pp.1078-1088.

Li, M., Li, S., Chavarro, J.E., Gaskins, A.J., Ley, S.H., Hinkle, S.N., Wang, X., Ding, M., Bell, G., Bjerregaard, A.A. and Olsen, S.F., 2019. Prepregnancy habitual intakes of total, supplemental, and food folate and risk of gestational diabetes mellitus: a prospective cohort study. *Diabetes Care*, *42*(6), pp.1034-1041.

Zhao, J.V., Schooling, C.M. and Zhao, J.X., 2018. The effects of folate supplementation on glucose metabolism and risk of type 2 diabetes: a systematic review and meta-analysis of randomized controlled trials. *Annals of Epidemiology*, *28*(4), pp.249-257.

Virtanen, J.K., Tuomainen, T.P. and Voutilainen, S., 2020. Dietary intake of choline and phosphatidylcholine and risk of type 2 diabetes in men: The Kuopio Ischaemic Heart Disease Risk Factor Study. *European Journal of Nutrition*, pp.1-5.

Svingen, G.F., Schartum-Hansen, H., Pedersen, E.R., Ueland, P.M., Tell, G.S., Mellgren, G., Njølstad, P.R., Seifert, R., Strand, E., Karlsson, T. and Nygård, O., 2016. Prospective associations of systemic and urinary choline metabolites with incident type 2 diabetes. *Clinical Chemistry*, *62*(5), pp.755-765.

Cigerli, O., Parildar, H., Unal, A.D., Tarcin, O., Kut, A., Eroglu, H. and Guvener, N., 2016. Vitamin deficiency and insulin resistance in nondiabetic obese patients. *Acta Endocrinologica (Bucharest)*, *12*(3), p.319.

Knight, B.A., Shields, B.M., Brook, A., Hill, A., Bhat, D.S., Hattersley, A.T. and Yajnik, C.S., 2015. Lower circulating B12 is associated with higher obesity and insulin resistance during pregnancy in a non-diabetic white British population. *PLoS One*, *10*(8), p.e0135268.

Krishnaveni, G.V., Hill, J.C., Veena, S.R., Bhat, D.S., Wills, A.K., Karat, C.L.S., Yajnik, C.S. and Fall, C.H.D., 2009. Low plasma vitamin B 12 in pregnancy is associated with gestational ‘diabesity’and later diabetes. *Diabetologia*, *52*(11), pp.2350-2358.

Baltaci, D., Kutlucan, A., Turker, Y., Yilmaz, A., Karacam, S., Deler, H., Ucgun, T. and Kara, I.H., 2013. Association of vitamin B12 with obesity, overweight, insulin resistance and metabolic syndrome, and body fat composition; primary care-based study. *Med Glas (Zenica)*, *10*(2), pp.203-210.

Ho, M., Halim, J.H., Gow, M.L., El-Haddad, N., Marzulli, T., Baur, L.A., Cowell, C.T. and Garnett, S.P., 2014. Vitamin B12 in obese adolescents with clinical features of insulin resistance. *Nutrients*, *6*(12), pp.5611-5618.

Harding, A.H., Wareham, N.J., Bingham, S.A., Khaw, K., Luben, R., Welch, A. and Forouhi, N.G., 2008. Plasma vitamin C level, fruit and vegetable consumption, and the risk of new-onset type 2 diabetes mellitus: the European prospective investigation of cancer–Norfolk prospective study. *Archives of Internal Medicine*, *168*(14), pp.1493-1499.

Zhou, C., Na, L., Shan, R., Cheng, Y., Li, Y., Wu, X. and Sun, C., 2016. Dietary vitamin C intake reduces the risk of type 2 diabetes in Chinese adults: HOMA-IR and T-AOC as potential mediators. *PLos One*, *11*(9), p.e0163571.

Kirii, K., Mizoue, T., Iso, H., Takahashi, Y., Kato, M., Inoue, M., Noda, M., Tsugane, S. and Japan Public Health Center-based Prospective Study Group, 2009. Calcium, vitamin D and dairy intake in relation to type 2 diabetes risk in a Japanese cohort. *Diabetologia*, *52*(12), pp.2542-2550.

Nsiah-Kumi, P.A., Erickson, J.M., Beals, J.L., Ogle, E.A., Whiting, M., Brushbreaker, C., Borgeson, C.D., Qiu, F., Yu, F. and Larsen, J.L., 2012. Vitamin D insufficiency is associated with diabetes risk in Native American children. *Clinical Pediatrics*, *51*(2), pp.146-153.

Mitri, J., Muraru, M.D. and Pittas, A.G., 2011. Vitamin D and type 2 diabetes: a systematic review. *European Journal of Clinical Nutrition*, *65*(9), pp.1005-1015.

Pittas, A.G., Sun, Q., Manson, J.E., Dawson-Hughes, B. and Hu, F.B., 2010. Plasma 25-hydroxyvitamin D concentration and risk of incident type 2 diabetes in women. *Diabetes Care*, *33*(9), pp.2021-2023.

Knekt, P., Laaksonen, M., Mattila, C., Härkänen, T., Marniemi, J., Heliövaara, M., Rissanen, H., Montonen, J. and Reunanen, A., 2008. Serum vitamin D and subsequent occurrence of type 2 diabetes. *Epidemiology*, pp.666-671.

Song, Y., Wang, L., Pittas, A.G., Del Gobbo, L.C., Zhang, C., Manson, J.E. and Hu, F.B., 2013. Blood 25-hydroxy vitamin D levels and incident type 2 diabetes: a meta-analysis of prospective studies. *Diabetes Care*, *36*(5), pp.1422-1428.

Afzal, S., Brøndum-Jacobsen, P., Bojesen, S.E. and Nordestgaard, B.G., 2014. Vitamin D concentration, obesity, and risk of diabetes: a mendelian randomisation study. *The Lancet Diabetes & Endocrinology*, *2*(4), pp.298-306.

Manna, P. and Kalita, J., 2016. Beneficial role of vitamin K supplementation on insulin sensitivity, glucose metabolism, and the reduced risk of type 2 diabetes: A review. *Nutrition*, *32*(7-8), pp.732-739.

Li, Y., peng Chen, J., Duan, L. and Li, S., 2018. Effect of vitamin K2 on type 2 diabetes mellitus: A review. *Diabetes Research and Clinical Practice*, *136*, pp.39-51.

Beulens, J.W., Grobbee, D.E., Sluijs, I., Spijkerman, A.M. and Van Der Schouw, Y.T., 2010. Dietary phylloquinone and menaquinones intakes and risk of type 2 diabetes. *Diabetes Care*, *33*(8), pp.1699-1705.

Villegas, R., Gao, Y.T., Dai, Q., Yang, G., Cai, H., Li, H., Zheng, W. and Shu, X.O., 2009. Dietary calcium and magnesium intakes and the risk of type 2 diabetes: the Shanghai Women’s Health Study. *The American Journal of Clinical Nutrition*, *89*(4), pp.1059-1067.

Liu, S., Song, Y., Ford, E.S., Manson, J.E., Buring, J.E. and Ridker, P.M., 2005. Dietary calcium, vitamin D, and the prevalence of metabolic syndrome in middle-aged and older US women. *Diabetes Care*, *28*(12), pp.2926-2932.

Kim, K.N., Oh, S.Y. and Hong, Y.C., 2018. Associations of serum calcium levels and dietary calcium intake with incident type 2 diabetes over 10 years: the Korean Genome and Epidemiology Study (KoGES). *Diabetology & Metabolic Syndrome*, *10*(1), p.50.

Jhuang, Y.H., Kao, T.W., Peng, T.C., Chen, W.L., Chang, P.K. and Wu, L.W., 2019. Serum Phosphorus as a Risk Factor of Metabolic Syndrome in the Elderly in Taiwan: A Large-Population Cohort Study. *Nutrients*, *11*(10), p.2340.

Kao, W.L., Folsom, A.R., Nieto, F.J., Mo, J.P., Watson, R.L. and Brancati, F.L., 1999. Serum and dietary magnesium and the risk for type 2 diabetes mellitus: the Atherosclerosis Risk in Communities Study. *Archives of Internal Medicine*, *159*(18), pp.2151-2159.

Song, Y., Manson, J.E., Buring, J.E. and Liu, S., 2004. Dietary magnesium intake in relation to plasma insulin levels and risk of type 2 diabetes in women. *Diabetes Care*, *27*(1), pp.59-65.

Fang, X., Wang, K., Han, D., He, X., Wei, J., Zhao, L., Imam, M.U., Ping, Z., Li, Y., Xu, Y. and Min, J., 2016. Dietary magnesium intake and the risk of cardiovascular disease, type 2 diabetes, and all-cause mortality: a dose–response meta-analysis of prospective cohort studies. *BMC Medicine*, *14*(1), p.210.

Simcox, J.A. and McClain, D.A., 2013. Iron and diabetes risk. *Cell Metabolism*, *17*(3), pp.329-341.

Li, H., Li, S.J., Zhao, Z., Li, X. and Liu, Z.M., 2008. Body iron stores and dietary iron intake in relation to diabetes in adults in North China. *Diabetes Care*, *31*(2), pp.285-286.

Bao, W., Rong, Y., Rong, S. and Liu, L., 2012. Dietary iron intake, body iron stores, and the risk of type 2 diabetes: a systematic review and meta-analysis. *BMC Medicine*, *10*(1), p.119.

Jiang, R., Ma, J., Ascherio, A., Stampfer, M.J., Willett, W.C. and Hu, F.B., 2004. Dietary iron intake and blood donations in relation to risk of type 2 diabetes in men: a prospective cohort study. *The American Journal of Clinical Nutrition*, *79*(1), pp.70-75.

Lee, D.H., Folsom, A.R. and Jacobs, D.R., 2004. Dietary iron intake and type 2 diabetes incidence in postmenopausal women: the Iowa Women’s Health Study. *Diabetologia*, *47*(2), pp.185-194.

Sun, Q., Van Dam, R.M., Willett, W.C. and Hu, F.B., 2009. Prospective study of zinc intake and risk of type 2 diabetes in women. *Diabetes Care*, *32*(4), pp.629-634.

Vashum, K.P., McEvoy, M., Shi, Z., Milton, A.H., Islam, M.R., Sibbritt, D., Patterson, A., Byles, J., Loxton, D. and Attia, J., 2013. Is dietary zinc protective for type 2 diabetes? Results from the Australian longitudinal study on women’s health. *BMC Endocrine Disorders*, *13*(1), p.40.

Drake, I., Hindy, G., Ericson, U. and Orho-Melander, M., 2017. A prospective study of dietary and supplemental zinc intake and risk of type 2 diabetes depending on genetic variation in SLC30A8. *Genes & Nutrition*, *12*(1), p.30.

Eshak, E.S., Iso, H., Maruyama, K., Muraki, I. and Tamakoshi, A., 2018. Associations between dietary intakes of iron, copper and zinc with risk of type 2 diabetes mellitus: A large population-based prospective cohort study. *Clinical Nutrition*, *37*(2), pp.667-674.

Laouali, N., MacDonald, C.J., Fatouhi, D.E., Mancini, F.R., Fagherazzi, G. and Boutron-Ruault, M.C., 2020. Dietary Copper-Zinc Ratio and Type 2 Diabetes Risk in Women: The E3N Cohort Study. *Current Developments in Nutrition*, *4*(Supplement_2), pp.1431-1431.

Feldman, R.D. and Schmidt, N.D., 1999. Moderate dietary salt restriction increases vascular and systemic insulin resistance. *American Journal of Hypertension*, *12*(6), pp.643-647.

Garg, R., Williams, G.H., Hurwitz, S., Brown, N.J., Hopkins, P.N. and Adler, G.K., 2011. Low-salt diet increases insulin resistance in healthy subjects. *Metabolism*, *60*(7), pp.965-968.

Radzeviciene, L. and Ostrauskas, R., 2017. Adding salt to meals as a risk factor of type 2 diabetes mellitus: a case–control study. *Nutrients*, *9*(1), p.67.

Ma, Y., He, F.J. and MacGregor, G.A., 2015. High salt intake: independent risk factor for obesity?. *Hypertension*, *66*(4), pp.843-849.

Chatterjee, R., Yeh, H.C., Shafi, T., Selvin, E., Anderson, C., Pankow, J.S., Miller, E. and Brancati, F., 2010. Serum and dietary potassium and risk of incident type 2 diabetes mellitus: The Atherosclerosis Risk in Communities (ARIC) study. *Archives of Internal Medicine*, *170*(19), pp.1745-1751.

Chatterjee, R., Colangelo, L.A., Yeh, H.C., Anderson, C.A., Daviglus, M.L., Liu, K. and Brancati, F.L., 2012. Potassium intake and risk of incident type 2 diabetes mellitus: the Coronary Artery Risk Development in Young Adults (CARDIA) Study. *Diabetologia*, *55*(5), pp.1295-1303.

Stranges, S., Sieri, S., Vinceti, M., Grioni, S., Guallar, E., Laclaustra, M., Muti, P., Berrino, F. and Krogh, V., 2010. A prospective study of dietary selenium intake and risk of type 2 diabetes. *BMC Public Health*, *10*(1), p.564.

Wei, J., Zeng, C., Gong, Q.Y., Yang, H.B., Li, X.X., Lei, G.H. and Yang, T.B., 2015. The association between dietary selenium intake and diabetes: a cross-sectional study among middle-aged and older adults. *Nutrition Journal*, *14*(1), p.18.

Vinceti, M., Filippini, T. and Rothman, K.J., 2018. Selenium exposure and the risk of type 2 diabetes: a systematic review and meta-analysis. *European Journal of Epidemiology* 33, 789–810. <https://doi.org/10.1007/s10654-018-0422-8>

Van Dam, R.M., Willett, W.C., Manson, J.E. and Hu, F.B., 2006. Coffee, caffeine, and risk of type 2 diabetes: a prospective cohort study in younger and middle-aged US women. *Diabetes Care*, *29*(2), pp.398-403.

Iso, H., Date, C., Wakai, K., Fukui, M. and Tamakoshi, A., 2006. The relationship between green tea and total caffeine intake and risk for self-reported type 2 diabetes among Japanese adults. *Annals of Internal Medicine*, *144*(8), pp.554-562.

Bhupathiraju, S.N., Pan, A., Malik, V.S., Manson, J.E., Willett, W.C., van Dam, R.M. and Hu, F.B., 2013. Caffeinated and caffeine-free beverages and risk of type 2 diabetes. *The American Journal of Clinical Nutrition*, *97*(1), pp.155-166.

Jiang, X., Zhang, D. and Jiang, W., 2014. Coffee and caffeine intake and incidence of type 2 diabetes mellitus: a meta-analysis of prospective studies. *European Journal of Nutrition*, *53*(1), pp.25-38.

Goto, A., Song, Y., Chen, B.H., Manson, J.E., Buring, J.E. and Liu, S., 2011. Coffee and caffeine consumption in relation to sex hormone–binding globulin and risk of type 2 diabetes in postmenopausal women. *Diabetes*, *60*(1), pp.269-275.

Mirmiran, P., Carlström, M., Bahadoran, Z. and Azizi, F., 2018. Long-term effects of coffee and caffeine intake on the risk of pre-diabetes and type 2 diabetes: findings from a population with low coffee consumption. *Nutrition, Metabolism and Cardiovascular Diseases*, *28*(12), pp.1261-1266.

Van Dam, R.M. and Feskens, E.J., 2002. Coffee consumption and risk of type 2 diabetes mellitus. *The Lancet*, *360*(9344), pp.1477-1478.

Salazar-Martinez, E., Willett, W.C., Ascherio, A., Manson, J.E., Leitzmann, M.F., Stampfer, M.J. and Hu, F.B., 2004. Coffee consumption and risk for type 2 diabetes mellitus. *Annals of Internal Medicine*, *140*(1), pp.1-8.

Van Dieren, S., Uiterwaal, C.S.P.M., Van der Schouw, Y.T., Boer, J.M.A., Spijkerman, A., Grobbee, D.E. and Beulens, J.W.J., 2009. Coffee and tea consumption and risk of type 2 diabetes. *Diabetologia*, *52*(12), pp.2561-2569.

Jang, M.H., Kang, N.H., Mukherjee, S. and Yun, J.W., 2018. Theobromine, a methylxanthine in cocoa bean, stimulates thermogenesis by inducing white fat browning and activating brown adipocytes. *Biotechnology and Bioprocess Engineering*, *23*(6), pp.617-626.

Greenberg, J.A., 2015. Chocolate intake and diabetes risk. *Clinical Nutrition*, *34*(1), pp.129-133.

Carroll, H.A., Davis, M.G. and Papadaki, A., 2015. Higher plain water intake is associated with lower type 2 diabetes risk: a cross-sectional study in humans. *Nutrition Research*, *35*(10), pp.865-872.

Pan, A., Malik, V.S., Schulze, M.B., Manson, J.E., Willett, W.C. and Hu, F.B., 2012. Plain-water intake and risk of type 2 diabetes in young and middle-aged women. *The American Journal of Clinical Nutrition*, *95*(6), pp.1454-1460.

Roussel, R., Fezeu, L., Bouby, N., Balkau, B., Lantieri, O., Alhenc-Gelas, F., Marre, M., Bankir, L. and DESIR Study Group, 2011. Low water intake and risk for new-onset hyperglycemia. *Diabetes Care*, *34*(12), pp.2551-2554.

Huth, P.J., Fulgoni, V., Jandacek, R.J., Jones, P.J., St‐Onge, M.P. and Senanayake, V., 2010. Bioactivity and emerging role of short and medium chain fatty acids. *Lipid Technology*, *22*(12), pp.266-269.

Liu, Shengxin, Yvonne T. van der Schouw, Sabita S. Soedamah-Muthu, Annemieke MW Spijkerman, and Ivonne Sluijs. 2019. Intake of dietary saturated fatty acids and risk of type 2 diabetes in the European Prospective Investigation into Cancer and Nutrition-Netherlands cohort: associations by types, sources of fatty acids and substitution by macronutrients. *European Journal of Nutrition* 58(3): 1125-1136.

Paquet, C., Propsting, S.L. and Daniel, M., 2014. Total n-3 fatty acid and SFA intakes in relation to insulin resistance in a Canadian First Nation at risk for the development of type 2 diabetes. *Public Health Nutrition*, *17*(6), pp.1337-1341.

Gaeini, Z., Bahadoran, Z., Mirmiran, P. and Djazayery, A., 2019. The association between dietary fat pattern and the risk of type 2 diabetes. *Preventive Nutrition and Food Science*, *24*(1), p.1.

Wanders, A.J., Alssema, M., De Koning, E.J.P., Le Cessie, S., De Vries, J.H., Zock, P.L., Rosendaal, F.R., den Heijer, M. and De Mutsert, R., 2017. Fatty acid intake and its dietary sources in relation with markers of type 2 diabetes risk: the NEO study. *European Journal of Clinical Nutrition*, *71*(2), pp.245-251.

Salmeron, J., Hu, F.B., Manson, J.E., Stampfer, M.J., Colditz, G.A., Rimm, E.B. and Willett, W.C., 2001. Dietary fat intake and risk of type 2 diabetes in women. *The American Journal of Clinical Nutrition*, *73*(6), pp.1019-1026.

ARIC Study Investigators. 2003. Plasma fatty acid composition and incidence of diabetes in middle-aged adults: the Atherosclerosis Risk in Communities (ARIC) Study, *The American Journal of Clinical Nutrition*, 78(1), pp91–98, <https://doi.org/10.1093/ajcn/78.1.91>

Unger, A.L., Torres-Gonzalez, M. and Kraft, J., 2019. Dairy fat consumption and the risk of metabolic syndrome: an examination of the saturated fatty acids in dairy. *Nutrients*, *11*(9), p.2200.

Wang, Y., Meng, X., Deng, X., Okekunle, A.P., Wang, P., Zhang, Q., Ding, L., Guo, X., Lv, M., Sun, C. and Li, Y., 2018. Postprandial saturated fatty acids increase the risk of type 2 diabetes: a cohort study in a chinese population. *The Journal of Clinical Endocrinology & Metabolism*, *103*(4), pp.1438-1446.

Imamura, F., Micha, R., Wu, J.H., de Oliveira Otto, M.C., Otite, F.O., Abioye, A.I. and Mozaffarian, D., 2016. Effects of saturated fat, polyunsaturated fat, monounsaturated fat, and carbohydrate on glucose-insulin homeostasis: a systematic review and meta-analysis of randomised controlled feeding trials. *PLoS Medicine*, *13*(7), p.e1002087.

Gillingham, L.G., Harris-Janz, S. and Jones, P.J., 2011. Dietary monounsaturated fatty acids are protective against metabolic syndrome and cardiovascular disease risk factors. *Lipids*, *46*(3), pp.209-228.

Guess, N., Perreault, L., Kerege, A., Strauss, A. and Bergman, B.C., 2016. Dietary fatty acids differentially associate with fasting versus 2-hour glucose homeostasis: implications for the management of subtypes of prediabetes. *PLoS One*, *11*(3), p.e0150148.

de Barros, C.R., Cezaretto, A., Curti, M.L.R., Pires, M.M., Folchetti, L.D., Siqueira-Catania, A. and Ferreira, S.R.G., 2014. Realistic changes in monounsaturated fatty acids and soluble fibers are able to improve glucose metabolism. *Diabetology & Metabolic Syndrome*, *6*(1), p.136.

Xiao, C., Giacca, A., Carpentier, A. and Lewis, G.F., 2006. Differential effects of monounsaturated, polyunsaturated and saturated fat ingestion on glucose-stimulated insulin secretion, sensitivity and clearance in overweight and obese, non-diabetic humans. *Diabetologia*, *49*(6), pp.1371-1379.

López, S., Bermúdez, B., Pacheco, Y.M., Villar, J., Abia, R. and Muriana, F.J., 2008. Distinctive postprandial modulation of β cell function and insulin sensitivity by dietary fats: monounsaturated compared with saturated fatty acids. *The American Journal of Clinical Nutrition*, *88*(3), pp.638-644.

Due, A., Larsen, T.M., Hermansen, K., Stender, S., Holst, J.J., Toubro, S., Martinussen, T. and Astrup, A., 2008. Comparison of the effects on insulin resistance and glucose tolerance of 6-mo high-monounsaturated-fat, low-fat, and control diets. *The American Journal of Clinical Nutrition*, *87*(4), pp.855-862.

Sartorius, T., Ketterer, C., Kullmann, S., Balzer, M., Rotermund, C., Binder, S., Hallschmid, M., Machann, J., Schick, F., Somoza, V. and Preissl, H., 2012. Monounsaturated fatty acids prevent the aversive effects of obesity on locomotion, brain activity, and sleep behavior. *Diabetes*, *61*(7), pp.1669-1679.

Zheng, J.S., Huang, T., Yang, J., Fu, Y.Q. and Li, D., 2012. Marine N-3 polyunsaturated fatty acids are inversely associated with risk of type 2 diabetes in Asians: a systematic review and meta-analysis. *PLoS One*, *7*(9), p.e44525.

Forouhi, N.G., Imamura, F., Sharp, S.J., Koulman, A., Schulze, M.B., Zheng, J., Ye, Z., Sluijs, I., Guevara, M., Huerta, J.M. and Kröger, J., 2016. Association of plasma phospholipid n-3 and n-6 polyunsaturated fatty acids with type 2 diabetes: the EPIC-InterAct Case-Cohort Study. *PLoS Medicine*, *13*(7), p.e1002094.

Yary, T., Voutilainen, S., Tuomainen, T.P., Ruusunen, A., Nurmi, T. and Virtanen, J.K., 2016. Serum n–6 polyunsaturated fatty acids, Δ 5-and Δ 6-desaturase activities, and risk of incident type 2 diabetes in men: the Kuopio Ischaemic Heart Disease Risk Factor Study. *The American Journal of Clinical Nutrition*, *103*(5), pp.1337-1343.

Lemetais, G., Melander, O., Vecchio, M., Bottin, J.H., Enhörning, S. and Perrier, E.T., 2018. Effect of increased water intake on plasma copeptin in healthy adults. *European Journal of Nutrition*, *57*(5), pp.1883-1890.

Brunkwall, L., Ericson, U., Nilsson, P.M. and Enhörning, S., 2020. High water intake and low urine osmolality are associated with favorable metabolic profile at a population level: low vasopressin secretion as a possible explanation. *European Journal of Nutrition*, pp.1-8.

Joshipura, K.J., Trak, M.A. and Munoz-Torres, F.J., 2018. Cross-sectional associations between drinking bottled water and prediabetes/diabetes. <https://doi.org/10.2337/db18-197-LB>

Del Razo, L.M., García-Vargas, G.G., Valenzuela, O.L., Castellanos, E.H., Sánchez-Peña, L.C., Currier, J.M., Drobná, Z., Loomis, D. and Stýblo, M., 2011. Exposure to arsenic in drinking water is associated with increased prevalence of diabetes: a cross-sectional study in the Zimapan and Lagunera regions in Mexico. *Environmental Health*, *10*(1), p.73.

Kao, W.L., Puddey, I.B., Boland, L.L., Watson, R.L. and Brancati, F.L., 2001. Alcohol consumption and the risk of type 2 diabetes mellitus: atherosclerosis risk in communities study. *American Journal of Epidemiology*, *154*(8), pp.748-757.

Hodge, A.M., English, D.R., O'dea, K. and Giles, G.G., 2006. Alcohol intake, consumption pattern and beverage type, and the risk of Type 2 diabetes. *Diabetic Medicine*, *23*(6), pp.690-697.

Carlsson, S., Hammar, N., Grill, V. and Kaprio, J., 2003. Alcohol consumption and the incidence of type 2 diabetes: a 20-year follow-up of the Finnish twin cohort study. *Diabetes Care*, *26*(10), pp.2785-2790.

Knott, C., Bell, S. and Britton, A., 2015. Alcohol consumption and the risk of type 2 diabetes: a systematic review and dose-response meta-analysis of more than 1.9 million individuals from 38 observational studies. *Diabetes Care*, *38*(9), pp.1804-1812.

Cullmann, M., Hilding, A. and Östenson, C.G., 2012. Alcohol consumption and risk of pre‐diabetes and type 2 diabetes development in a Swedish population. *Diabetic Medicine*, *29*(4), pp.441-452.

Zhang, S., Liu, Y., Wang, G., Xiao, X., Gang, X., Li, F., Sun, C., Gao, Y. and Wang, G., 2016. The relationship between alcohol consumption and incidence of glycometabolic abnormality in middle-aged and elderly Chinese men. *International Journal of Endocrinology*, *2016*.

Kouroglou, E., Anagnostis, P., Daponte, A. and Bargiota, A., 2019. Vitamin B12 insufficiency is associated with increased risk of gestational diabetes mellitus: a systematic review and meta-analysis*. Endocrine*. 66(2):149-156. doi:10.1007/s12020-019-02053-1

Atsma, F., Veldhuizen, I., Verbeek, A., de Kort, W. and de Vegt, F., 2011. Healthy donor effect: its magnitude in health research among blood donors. *Transfusion*, *51*(8), pp.1820-1828.

De Wit, S., Sabin, C.A., Weber, R., Worm, S.W., Reiss, P., Cazanave, C., El-Sadr, W., Monforte, A.D.A., Fontas, E., Law, M.G. and Friis-Møller, N., 2008. Incidence and risk factors for new-onset diabetes in HIV-infected patients: the Data Collection on Adverse Events of Anti-HIV Drugs (D: A: D) study. *Diabetes Care*, *31*(6), pp.1224-1229.

Beach, L.B., Elasy, T.A. and Gonzales, G., 2018. Prevalence of self-reported diabetes by sexual orientation: Results from the 2014 Behavioral Risk Factor Surveillance System. *LGBT health*, *5*(2), pp.121-130.

Hu, F.B., Sigal, R.J., Rich-Edwards, J.W., Colditz, G.A., Solomon, C.G., Willett, W.C., Speizer, F.E. and Manson, J.E., 1999. Walking compared with vigorous physical activity and risk of type 2 diabetes in women: a prospective study. *JAMA*, *282*(15), pp.1433-1439.

Krishnan, S., Rosenberg, L. and Palmer, J.R., 2009. Physical activity and television watching in relation to risk of type 2 diabetes: the Black Women's Health Study. *American Journal of Epidemiology*, *169*(4), pp.428-434.

Williams, P.T., 2007. Changes in vigorous physical activity and incident diabetes in male runners. *Diabetes Care*, *30*(11), pp.2838-2842.

Ekelund, U., Brage, S., Griffin, S.J. and Wareham, N.J., 2009. Objectively measured moderate-and vigorous-intensity physical activity but not sedentary time predicts insulin resistance in high-risk individuals. *Diabetes Care*, *32*(6), pp.1081-1086.

Aune, D., Norat, T., Leitzmann, M., Tonstad, S. and Vatten, L.J., 2015. Physical activity and the risk of type 2 diabetes: a systematic review and dose–response meta-analysis. *European Journal of Epidemiology* 30, 529–542. <https://doi.org/10.1007/s10654-015-0056-z>

Yerramalla, M.S., Fayosse, A., Dugravot, A., Tabak, A.G., Kivimäki, M., Singh-Manoux, A. and Sabia, S., 2020. Association of moderate and vigorous physical activity with incidence of type 2 diabetes and subsequent mortality: 27 year follow-up of the Whitehall II study. *Diabetologia*, *63*(3), pp.537-548.

Joseph, J.J., Echouffo-Tcheugui, J.B., Golden, S.H., Chen, H., Jenny, N.S., Carnethon, M.R., Jacobs, D., Burke, G.L., Vaidya, D., Ouyang, P. and Bertoni, A.G., 2016. Physical activity, sedentary behaviors and the incidence of type 2 diabetes mellitus: the Multi-Ethnic Study of Atherosclerosis (MESA). *BMJ Open Diabetes Research and Care*, *4*(1).

Williams, P.T. and Thompson, P.D., 2013. Walking versus running for hypertension, cholesterol, and diabetes mellitus risk reduction. *Arteriosclerosis, Thrombosis, and Vascular Biology*, *33*(5), pp.1085-1091.

Demakakos, P., Hamer, M., Stamatakis, E. and Steptoe, A., 2010. Low-intensity physical activity is associated with reduced risk of incident type 2 diabetes in older adults: evidence from the English Longitudinal Study of Ageing. *Diabetologia*, *53*(9), pp.1877-1885.

Jeon, C.Y., Lokken, R.P., Hu, F.B. and Van Dam, R.M., 2007. Physical activity of moderate intensity and risk of type 2 diabetes: a systematic review. *Diabetes Care*, *30*(3), pp.744-752.

Okada, K., Hayashi, T., Tsumura, K., Suematsu, C., Endo, G. and Fujii, S., 2000. Leisure‐time physical activity at weekends and the risk of Type 2 diabetes mellitus in Japanese men: the Osaka Health Survey. *Diabetic Medicine*, *17*(1), pp.53-58.

Rasmussen, M.G., Grøntved, A., Blond, K., Overvad, K., Tjønneland, A., Jensen, M.K. and Østergaard, L., 2016. Associations between recreational and commuter cycling, changes in cycling, and type 2 diabetes risk: a cohort study of Danish men and women. *PLoS Medicine*, *13*(7), p.e1002076.

Riiser, A., Solbraa, A., Jenum, A.K., Birkeland, K.I. and Andersen, L.B., 2018. Cycling and walking for transport and their associations with diabetes and risk factors for cardiovascular disease. *Journal of Transport & Health*, *11*, pp.193-201.

Grøntved, A., Rimm, E.B., Willett, W.C., Andersen, L.B. and Hu, F.B., 2012. A prospective study of weight training and risk of type 2 diabetes mellitus in men. *Archives of Internal Medicine*, *172*(17), pp.1306-1312.

Robinson, E., Durrer, C., Simtchouk, S., Jung, M.E., Bourne, J.E., Voth, E. and Little, J.P., 2015. Short-term high-intensity interval and moderate-intensity continuous training reduce leukocyte TLR4 in inactive adults at elevated risk of type 2 diabetes. *Journal of Applied Physiology*, *119*(5), pp.508-516.

Fisher, G., Brown, A.W., Brown, M.M.B., Alcorn, A., Noles, C., Winwood, L., Resuehr, H., George, B., Jeansonne, M.M. and Allison, D.B., 2015. High intensity interval-vs moderate intensity-training for improving cardiometabolic health in overweight or obese males: a randomized controlled trial. *PLoS One*, *10*(10), p.e0138853.

Vgontzas, A.N., Liao, D., Pejovic, S., Calhoun, S., Karataraki, M. and Bixler, E.O., 2009. Insomnia with objective short sleep duration is associated with type 2 diabetes: a population-based study. *Diabetes care*, *32*(11), pp.1980-1985.

Lin, C.L., Chien, W.C., Chung, C.H. and Wu, F.L., 2018. Risk of type 2 diabetes in patients with insomnia: A population‐based historical cohort study. *Diabetes/Metabolism Research and Reviews*, *34*(1), p.e2930.

Carroll, J.E., Seeman, T.E., Olmstead, R., Melendez, G., Sadakane, R., Bootzin, R., Nicassio, P. and Irwin, M.R., 2015. Improved sleep quality in older adults with insomnia reduces biomarkers of disease risk: pilot results from a randomized controlled comparative efficacy trial. *Psychoneuroendocrinology*, *55*, pp.184-192.

LeBlanc, E.S., Smith, N.X., Nichols, G.A., Allison, M.J. and Clarke, G.N., 2018. Insomnia is associated with an increased risk of type 2 diabetes in the clinical setting. *BMJ Open Diabetes Research and Care*, *6*(1).

Willi, C., Bodenmann, P., Ghali, W.A., Faris, P.D. and Cornuz, J., 2007. Active smoking and the risk of type 2 diabetes: a systematic review and meta-analysis. *JAMA*, *298*(22), pp.2654-2664.

Rimm, E.B., Chan, J., Stampfer, M.J., Colditz, G.A. and Willett, W.C., 1995. Prospective study of cigarette smoking, alcohol use, and the risk of diabetes in men. *BMJ*, *310*(6979), pp.555-559.

Yeh, H.C., Duncan, B.B., Schmidt, M.I., Wang, N.Y. and Brancati, F.L., 2010. Smoking, smoking cessation, and risk for type 2 diabetes mellitus: a cohort study. *Annals of Internal Medicine*, *152*(1), pp.10-17.

Luo, J., Rossouw, J., Tong, E., Giovino, G.A., Lee, C.C., Chen, C., Ockene, J.K., Qi, L. and Margolis, K.L., 2013. Smoking and diabetes: does the increased risk ever go away?. *American Journal of Epidemiology*, *178*(6), pp.937-945.

Rimm, E.B., Manson, J.E., Stampfer, M.J., Colditz, G.A., Willett, W.C., Rosner, B., Hennekens, C.H. and Speizer, F.E., 1993. Cigarette smoking and the risk of diabetes in women. *American Journal of Public Health*, *83*(2), pp.211-214.

Wannamethee, S.G., Shaper, A.G. and Perry, I.J., 2001. Smoking as a modifiable risk factor for type 2 diabetes in middle-aged men. *Diabetes Care*, *24*(9), pp.1590-1595.

Beziaud, F., Halimi, J.M., Lecomte, P. and Tichet, J., 2004. Cigarette smoking and diabetes mellitus. *Diabetes & Metabolism*, *30*(2), pp.161-166.

Pan, A., Wang, Y., Talaei, M., Hu, F.B. and Wu, T., 2015. Relation of active, passive, and quitting smoking with incident type 2 diabetes: a systematic review and meta-analysis. *The Lancet Diabetes & Endocrinology*, *3*(12), pp.958-967.

Jee, S.H., Foong, A.W., Hur, N.W. and Samet, J.M., 2010. Smoking and risk for diabetes incidence and mortality in Korean men and women. *Diabetes Care*, *33*(12), pp.2567-2572.

Wennberg, P., Rolandsson, O., Spijkerman, A.M., Kaaks, R., Boeing, H., Feller, S., Bergmann, M.M., Langenberg, C., Sharp, S.J., Forouhi, N. and Riboli, E., 2013. Self-rated health and type 2 diabetes risk in the European Prospective Investigation into Cancer and Nutrition-InterAct study: a case-cohort study. *BMJ Open*, *3*(3).

Noh, J.W., Chang, Y., Park, M., Kwon, Y.D. and Ryu, S., 2019. Self-rated health and the risk of incident type 2 diabetes mellitus: A cohort study. *Scientific Reports*, *9*(1), pp.1-8.

Hu, F.B., Li, T.Y., Colditz, G.A., Willett, W.C. and Manson, J.E., 2003. Television watching and other sedentary behaviors in relation to risk of obesity and type 2 diabetes mellitus in women. *JAMA*, *289*(14), pp.1785-1791.

Patterson, R., McNamara, E., Tainio, M., de Sá, T.H., Smith, A.D., Sharp, S.J., Edwards, P., Woodcock, J., Brage, S. and Wijndaele, K., 2018. Sedentary behaviour and risk of all-cause, cardiovascular and cancer mortality, and incident type 2 diabetes: a systematic review and dose response meta-analysis. *European Journal of Epidemiology* 33, 811–829. <https://doi.org/10.1007/s10654-018-0380-1>
