# Supplementary material for: Nutritional markers of undiagnosed type 2 diabetes in adults: Findings of a machine learning analysis with external validation and benchmarking
Source: PLoS One. 2021 May 5;16(5):e0250832. doi: 10.1371/journal.pone.0250832 (PMC8099133; doi:10.1371/journal.pone.0250832)
Supplement: S2 Table — (DOCX) [file pone.0250832.s002.docx]

**S2 Table: Distribution of features in the entire cohort (N=16429) between undiagnosed T2D and non-T2D (normoglycaemic) individuals.**

| **Variable** | **Non-T2D**  **(n=15564)** | **Undiagnosed T2D (n=865)** | **p-value^a^** |
| --- | --- | --- | --- |
| **Categorical variables** | **n (%)** | **n (%)** |  |
| **Gender ^b^** |  |  |  |
| Male | 7236(46.49) | 455 (52.60) | **0.0004** |
| Female | 8328 (53.51) | 410 (47.40) |  |
| **Race ^b^** |  |  |  |
| (Non-Hispanic) White | 6719 (43.17) | 291(33.64) | **<0.0001** |
| Other**^c^** | 8845 (56.83) | 574 (66.36) |  |
| **Citizenship ^b^** |  |  |  |
| Yes | 13261 (85.20) | 698 (80.69) | **0.0003** |
| No | 2303 (14.80) | 167 (19.31) |  |
| **Marital status ^b^** |  |  |  |
| Married/ Living with partner | 9105 (58.50) | 527 (60.92) | NS |
| Other**^d^** | 6459 (41.50) | 338 (39.08) |  |
| **Alcohol use ^b, e^** |  |  |  |
| No | 4249 (27.3) | 215 (24.86) | NS |
| Yes | 11315 (72.7) | 650 (75.14) |  |
| **Donated blood in past 12 months ^b^** |  |  |  |
| No | 14835 (95.32) | 836 (96.65) | NS |
| Yes | 729 (4.68) | 29 (3.35) |  |
| **Blood ever tested for HIV? ^b^** |  |  |  |
| No | 9216 (59.21) | 614 (70.98) | **<0.0001** |
| Yes | 6348 (40.79) | 251 (29.02) |  |
| **HH Emergency food received ^b^** |  |  |  |
| No | 14029 (90.14) | 758 (87.63) | **0.0167** |
| Yes | 1535 (9.86) | 107 (12.37) |  |
| **HH FS benefit: ever received ^b^** |  |  |  |
| No | 10921 (70.17) | 627 (72.48) | NS |
| Yes | 4643 (29.83) | 238 (27.52) |  |
| **Vigorous work activity ^b^** |  |  |  |
| No | 12520 (80.44) | 726 (83.93) | **0.0115** |
| Yes | 3044 (19.56) | 139 (16.07) |  |
| **Moderate work activity ^b^** |  |  |  |
| No | 9951 (63.94) | 608 (70.29) | **0.0001** |
| Yes | 5613 (36.06) | 257 (29.71) |  |
| **Walk or bicycle ^b^** |  |  |  |
| No | 11329 (72.79) | 679 (78.50) | **0.0002** |
| Yes | 4235 (27.21) | 186 (21.50) |  |
| **Vigorous recreational activities ^b^** |  |  |  |
| No | 11590 (74.47) | 783 (90.52) | **< 0.0001** |
| Yes | 3974 (25.53) | 82 (9.48) |  |
| **Moderate recreational activities ^b^** |  |  |  |
| No | 8958 (57.56) | 622 (71.91) | **< 0.0001** |
| Yes | 6606 (42.44) | 243 (28.09) |  |
| **Ever told doctor had trouble sleeping? ^b^** |  |  |  |
| No | 11971 (76.92) | 649 (75.03) | NS |
| Yes | 3593 (23.08) | 216 (24.97) |  |
| **Smoked at least 100 cigarettes in life ^b^** |  |  |  |
| No | 8961 (57.58) | 428 (49.48) | **< 0.0001** |
| Yes | 6603 (42.42) | 437 (50.52) |  |
| **How do you consider your weight ^b^** |  |  |  |
| Underweight | 973 (6.25) | 31 (3.58) | **0.0014** |
| About the right weight | 7119 (45.74) | 305 (35.26) | **<0.0001** |
| Overweight | 7472 (48.01) | 529 (61.16) | **<0.0001** |
| **Like to weigh more, less or same ^b^** |  |  |  |
| More | 1430 (9.19) | 36 (4.16) | **< 0.0001** |
| Stay about the same | 5091 (32.71) | 251 (29.02) | **0.0241** |
| Less | 9043 (58.10) | 578 (66.82) | **<0.0001** |
| **Tried to lose weight in past year ^b^** |  |  |  |
| No | 9906 (63.65) | 535 (61.85) | NS |
| Yes | 5658 (36.35) | 330 (38.15) |  |
| **Type of table salt used ^b^** |  |  |  |
| Ordinary salt | 10282 (66.06) | 508 (58.72) | **<0.0001** |
| Salt substitutes/other | 841 (5.40) | 52 (6.01) | NS |
| Doesn’t use/add salt products at the table | 4441 (28.54) | 305 (35.27) | **<0.0001** |
| **On special diet? ^b^** |  |  |  |
| No | 13568 (87.17) | 740 (85.55) | NS |
| Yes | 1996 (12.83) | 125 (14.45) |  |
| **Compare food consumed yesterday to usual ^b^** |  |  |  |
| Much more than usual | 1486 (9.55) | 95 (10.98) | NS |
| Usual | 11288 (72.53) | 608 (70.29) | NS |
| Much less than usual | 2790 (17.92) | 162 (18.73) | NS |
| **Tap water source ^b^** |  |  |  |
| Community supply | 10832 (69.60) | 568 (65.66) | **0.0144** |
| Well or rain cistern | 1323 (8.50) | 73 (8.45) | NS |
| Spring | 142 (0.91) | 11 (1.27) | NS |
| Don't drink tap water | 3267 (20.99) | 213 (24.62) | **0.0110** |
| **Shellfish eaten during past 30 days ^b^** |  |  |  |
| No | 7245 (46.55) | 440 (50.87) | **0.0132** |
| Yes | 8319 (53.45) | 425 (49.13) |  |
| **Fish eaten during past 30 days ^b^** |  |  |  |
| No | 4323 (27.78) | 254 (29.36) | NS |
| Yes | 11241 (72.22) | 611 (70.64) |  |
| **Any dietary supplements taken? ^b^** |  |  |  |
| No | 8105 (52.07) | 489 (56.53) | **0.0106** |
| Yes | 7459 (47.93) | 376 (43.47) |  |
| **Any Antacids Taken? ^b^** |  |  |  |
| No | 13787 (88.58) | 766 (88.56) | NS |
| Yes | 1777 (11.42) | 99 (11.44) |  |
| **Numeric variables** | | | |
| **Variable** | **Mean (SD)** | **Mean; SD** | **p-value*** |
| Age (years) **^b^** | 46.4 (17.2) | 58.3 (14.8) | **<0.0001** |
| Education level ^b, f^ | 3.50 (1.25) | 3.00 (1.31) | **<0.0001** |
| Total no: of people in the household ^b^ | 3.28 (1.68) | 3.21 (1.74) | NS |
| Total no: of people in the family ^b^ | 3.07 (1.73) | 3.09 (1.76) | NS |
| Income-poverty ratio | 2.61 (1.65) | 2.32 (1.56) | **<0.0001** |
| Money spent at supermarket/grocery store ^b^ | 440 (1974) | 426 (358) | NS |
| Money spent on nonfood items ^b^ | 36.5 (67.2) | 36.2 (72.7) | NS |
| Money spent on food at other stores ^b^ | 82.0 (152.0) | 83.1 (147.0) | NS |
| Money spent on eating out ^b^ | 115 (166) | 146 (204) | **<0.0001** |
| Money spent on carryout/delivered foods ^b^ | 25.8 (98.8) | 19.6 (46.0) | NS |
| Self-rated general health ^b, f^ | 3.07 (0.987) | 2.69 (0.960) | **<0.0001** |
| How healthy is the diet ^b, f^ | 3.08 (1.01) | 2.95 (1.02) | **0.0002** |
| Past 30-day milk product consumption ^b, f^ | 1.81 (1.11) | 1.93 (1.10) | **0.0020** |
| Regular milk use 5 times per week ^b, f^ | 1.16 (0.781) | 1.19 (0.791) | NS |
| # of meals not home prepared ^b^ | 2.60 (3.53) | 3.42 (3.94) | **<0.0001** |
| # of meals from fast food or pizza place ^b^ | 1.34 (2.18) | 1.75 (2.57) | **<0.0001** |
| # of ready-to-eat foods in past 30 days ^b^ | 1.35 (4.60) | 2.08 (7.18) | **<0.0001** |
| # of frozen meals/pizza in past 30 days ^b^ | 1.93 (8.24) | 2.51 (7.10) | **0.0425** |
| HH Worried run out of food ^b, f^ | 2.64 (0.647) | 2.60 (0.692) | NS |
| HH Food didn't last ^b, f^ | 2.64 (0.658) | 2.71 (0.583) | **0.0022** |
| HH Couldn't afford balanced meals ^b, f^ | 2.72 (0.571) | 2.76 (0.547) | **0.0445** |
| Household food security category ^f^ | 3.43 (0.959) | 3.36 (1.02) | **0.0373** |
| Adult food security category ^f^ | 3.44 (0.961) | 3.37 (1.01) | **0.0376** |
| Monthly family income ^b, f^ | 6.85 (3.25) | 6.41 (3.01) | **0.0001** |
| Family monthly income-poverty level index | 2.34 (1.58) | 2.13 (1.48) | **0.0001** |
| Family monthly income-poverty level category ^f^ | 2.16 (0.912) | 2.04 (0.919) | **0.0002** |
| Minutes sedentary activity ^b^ | 342 (205) | 357 (203) | **0.0361** |
| How much sleep do you get (hours)? ^b^ | 7.00 (1.46) | 7.01 (1.61) | NS |
| Weight (kg) | 78.5 (20.2) | 88.4 (24.2) | **<0.0001** |
| Standing height (cm) | 167 (10.1) | 166 (10.2) | **0.0046** |
| Body mass index (kg/m^2^) | 28.0 (6.44) | 32.0 (7.42) | **<0.0001** |
| Upper leg length (cm) | 38.8 (3.79) | 37.1 (4.20) | **<0.0001** |
| Upper arm length (cm) | 37.4 (2.85) | 37.1 (2.79) | **0.0026** |
| Arm circumference (cm) | 32.4 (5.00) | 34.8 (5.36) | **<0.0001** |
| Waist circumference (cm) | 96.0 (15.5) | 108 (16.3) | **<0.0001** |
| Current self-reported height (inches) ^b^ | 66.3 (4.14) | 65.9 (4.26) | **0.0058** |
| Current self-reported weight (pounds) ^b^ | 172 (42.7) | 193 (50.1) | **<0.0001** |
| Self-reported weight-1 year ago (pounds) ^b^ | 172 (44.4) | 192 (49.8) | **<0.0001** |
| Self-reported greatest weight (pounds) ^b^ | 187 (49.3) | 208 (55.7) | **<0.0001** |
| Age when heaviest weight ^b^ | 39.0 (16.0) | 49.8 (16.1) | **< 0.0001** |
| Salt used in preparation? ^b, d^ | 3.12 (0.938) | 3.11 (0.967) | NS |
| Total # of dietary supplements taken ^b^ | 1.10 (1.78) | 0.990 (1.76) | NS |
| Total # of antacids taken ^b^ | 0.117 (0.330) | 0.116 (0.324) | NS |
| Average number of foods reported ^b^ | 16.5 (5.61) | 16.3 (5.40) | NS |
| Energy (kcal) ^g^ | 2001 (782) | 2096 (792) | **0.0005** |
| Protein (gm) ^g^ | 82.4 (35.5) | 79.9 (33.8) | **0.0433** |
| Carbohydrate (gm) ^g^ | 238 (101) | 252 (104) | **<0.0001** |
| Total sugars (gm) ^g^ | 111 (65.0) | 115 (65.4) | NS |
| Dietary fiber (gm) ^g^ | 17.7 (9.28) | 17.3 (8.81) | NS |
| Total fat (gm) ^g^ | 75.4 (36.4) | 78.5 (36.5) | **0.0148** |
| Total saturated fatty acids (gm) ^g^ | 23.4 (12.4) | 24.5 (12.9) | **0.0113** |
| Total monounsaturated fatty acids (gm) ^g^ | 26.9 (14.0) | 25.6 (14.1) | **0.0079** |
| Total polyunsaturated fatty acids (gm) ^g^ | 20.2 (13.0) | 19.6 (13.6) | NS |
| Cholesterol (mg) ^g^ | 302 (209) | 304 (206) | NS |
| Vitamin E as alpha-tocopherol (mg) ^g^ | 10.7 (11.4) | 10.5 (9.57) | NS |
| Added alpha-tocopherol (Vitamin E) (mg) ^g^ | 3.36 (8.22) | 2.72 (7.15) | **0.0249** |
| Retinol (mcg) ^g^ | 436 (391) | 434 (370) | NS |
| Vitamin A, RAE (mcg) ^g^ | 688 (516) | 671 (457) | NS |
| Alpha-carotene (mcg) ^g^ | 541 (1056) | 535 (919) | NS |
| Beta-carotene (mcg) ^g^ | 2705 (3557) | 2510 (2907) | NS |
| Beta-cryptoxanthin (mcg) ^g^ | 118 (289) | 112 (305) | NS |
| Lycopene (mcg) ^g^ | 5776 (8302) | 6046 (10002) | NS |
| Lutein + Zeaxanthin (mcg) ^g^ | 1764 (2833) | 1538 (1910) | **0.0205** |
| Thiamin (vitamin B1) (mg) ^g^ | 1.66 (0.819) | 1.59 (0.718) | **0.0138** |
| Riboflavin (vitamin B2) (mg) ^g^ | 2.28 (1.29) | 2.26 (1.43) | NS |
| Niacin (mg) ^g^ | 28.4 (16.1) | 27.8 (17.5) | NS |
| Vitamin B6 (mg) ^g^ | 2.64 (2.14) | 2.72 (2.38) | NS |
| Total folate (mcg) ^g^ | 401 (208) | 370 (185) | **<0.0001** |
| Folic acid (mcg) ^g^ | 185 (155) | 170 (140) | **0.0054** |
| Food folate (mcg) ^g^ | 216 (119) | 200 (99.9) | **0.0001** |
| Folate, DFE (mcg) ^g^ | 530 (304) | 489 (274) | **0.0001** |
| Total choline (mg) ^g^ | 352 (181) | 354 (179) | NS |
| Vitamin B12 (mcg) ^g^ | 7.29 (8.19) | 6.66 (6.87) | **0.0265** |
| Added vitamin B12 (mcg) ^g^ | 2.00 (3.79) | 2.09 (4.07) | NS |
| Vitamin C (mg) ^g^ | 112 (111) | 105 (97.5) | NS |
| Vitamin D (D2 + D3) (mcg) ^g^ | 6.97 (7.85) | 6.31 (7.49) | **0.0159** |
| Vitamin K (mcg) ^g^ | 132 (145) | 124 (117) | NS |
| Calcium (mg) ^g^ | 973 (509) | 964 (510) | NS |
| Phosphorus (mg) ^g^ | 1377 (573) | 1332 (545) | **0.0242** |
| Magnesium (mg) ^g^ | 312 (140) | 303 (135) | NS |
| Iron (mg) ^g^ | 15.3 (7.79) | 15.0 (7.25) | NS |
| Zinc (mg) ^g^ | 12.8 (10.1) | 13.2 (12.3) | NS |
| Copper (mg) ^g^ | 1.37 (0.916) | 1.35 (0.982) | NS |
| Sodium (mg) ^g^ | 3456 (1535) | 3316 (1597) | **0.0092** |
| Potassium (mg) ^g^ | 2783 (1135) | 2799 (1187) | NS |
| Selenium (mcg) ^g^ | 117 (59.5) | 113 (57.2) | NS |
| Caffeine (mg) ^g^ | 151 (183) | 132 (162) | **0.0028** |
| Theobromine (mg) ^g^ | 39.9 (65.9) | 38.6 (63.1) | NS |
| Alcohol (gm) ^g^ | 11.9 (24.1) | 11.5 (22.6) | NS |
| Moisture (gm) ^g^ | 2913 (1282) | 2818 (1281) | **0.0326** |
| SFA 4:0 (butanoic) (gm) ^g^ | 0.473 (0.383) | 0.490 (0.405) | NS |
| SFA 6:0 (hexanoic) (gm) ^g^ | 0.287 (0.239) | 0.283 (0.235) | NS |
| SFA 8:0 (octanoic) (gm) ^g^ | 0.246 (0.212) | 0.239 (0.205) | NS |
| SFA 10:0 (decanoic) (gm) ^g^ | 0.426 (0.320) | 0.454 (0.344) | **0.0126** |
| SFA 12:0 (dodecanoic) (gm) ^g^ | 0.701 (0.863) | 0.774 (0.955) | **0.0161** |
| SFA 14:0 (tetradecanoic) (gm) ^g^ | 1.96 (1.34) | 2.05 (1.43) | NS |
| SFA 16:0 (hexadecanoic) (gm) ^g^ | 12.8 (6.62) | 13.3 (6.80) | **0.0309** |
| SFA 18:0 (octadecanoic) (gm) ^g^ | 5.88 (3.15) | 6.07 (3.29) | NS |
| MFA 16:1 (hexadecenoic) (gm) ^g^ | 1.10 (0.687) | 1.10 (0.702) | NS |
| MFA 18:1 (octadecenoic) (gm) ^g^ | 25.0 (13.1) | 23.9 (13.3) | **0.0139** |
| MFA 20:1 (eicosenoic) (gm) ^g^ | 0.310 (0.28) | 0.303 (0.28) | NS |
| MFA 22:1 (docosenoic) (gm) ^g^ | 0.057 (0.198) | 0.062 (0.212) | NS |
| PFA 18:2 (octadecadienoic) (gm) ^g^ | 17.4 (10.6) | 16.8 (11.1) | NS |
| PFA 18:3 (octadecatrienoic) (gm) ^g^ | 2.25 (2.32) | 2.33 (2.53) | NS |
| PFA 18:4 (octadecatetraenoic) (gm) ^g^ | 0.0253 (0.0604) | 0.0266 (0.0583) | NS |
| PFA 20:4 (eicosatetraenoic) (gm) ^g^ | 0.163 (0.132) | 0.160 (0.128) | NS |
| PFA 20:5 (eicosapentaenoic) (gm) ^g^ | 0.0844 (0.212) | 0.0934 (0.212) | NS |
| PFA 22:5 (docosapentaenoic) (gm) ^g^ | 0.0365 (0.0645) | 0.0381 (0.0672) | NS |
| PFA 22:6 (docosahexaenoic) (gm) ^g^ | 0.176 (0.381) | 0.155 (0.352) | NS |
| Total plain water drank yesterday (gm) ^b, g^ | 1034 (941) | 960 (869) | **0.0237** |
| Total tap water drank yesterday (gm) ^b, g^ | 590 (824) | 525 (718) | **0.0238** |
| Total bottled water drank yesterday (gm) ^b, g^ | 445 (695) | 435 (668) | NS |

a-Chi-squared test for 2 proportions and 2-samples t-test were used for univariate analyses of categorical and continuous variables, respectively. Level of significance p = 0.05; b = self-reported data; c = Mexican American, other Hispanic, non-Hispanic Black, non-Hispanic Asian & other races including multi-racial; d = widowed, divorced, separated or never-married; e = defined as use of at least 12 drinks of any alcoholic beverage in any 1 year; f = modelled as continuous variables; g = Average of the two-day total intakes, where measured/reported and single-day total intakes otherwise.

FS = Food Stamps Program; HH = household; HIV = human immunodeficiency virus; MFA = mono unsaturated fatty Acids; NS = not significant; PFA = poly unsaturated fatty acids; SD = standard deviation; SFA = saturated fatty acids; T2D = type 2 diabetes.
