# Supplementary material for: Nutritional markers of undiagnosed type 2 diabetes in adults: Findings of a machine learning analysis with external validation and benchmarking
Source: PLoS One. 2021 May 5;16(5):e0250832. doi: 10.1371/journal.pone.0250832 (PMC8099133; doi:10.1371/journal.pone.0250832)
Supplement: S3 Table — (DOCX) [file pone.0250832.s003.docx]

**S3 Table: Comparison of algorithmic performance across internally- and externally-validated models.**

| Comparison of algorithmic performance: internally-validated models* | | | | | | | | |
| --- | --- | --- | --- | --- | --- | --- | --- | --- |
| Logistic regression | Random forests | | | | Artificial neural network | | | |
|  | Unbalanced  AUC=0.709 | Oversampled  AUC=0.743 | ROSE  AUC=0.752 | SMOTE  AUC=0.748 | Unbalanced  AUC=0.717 | Oversampled  AUC=0.72 | ROSE  AUC=0.749 | SMOTE  AUC=0.749 |
| Unbalanced AUC = 0.757 | P=0.051751 | P=0.565088 | P=0.836479 | P=0.710861 | P=0.104035 | P=0.132221 | P=0.741626 | P=0.741626 |
| Oversampled  AUC = 0.746 | P=0.135883 | P=0.902424 | P=0.80545 | P=0.934726 | P=0.241117 | P=0.292754 | P=0.902187 | P=0.902187 |
| ROSE  AUC = 0.735 | P=0.297041 | P=0.744954 | P=0.487557 | P=0.59625 | P=0.469066 | P=0.545844 | P=0.568135 | P=0.568135 |
| SMOTE  AUC = 0.735 | P=0.297041 | P=0.744954 | P=0.487557 | P=0.59625 | P=0.469066 | P=0.545844 | P=0.568135 | P=0.568135 |
| Comparison of algorithmic performance: externally-validated models* | | | | | | | | |
| Logistic regression | Random forests | | | | Artificial neural network | | | |
|  | Unbalanced  AUC=0.715 | Oversampled  AUC=0.741 | ROSE  AUC=0.743 | SMOTE  AUC=0.752 | Unbalanced  AUC=0.718 | Oversampled  AUC=0.727 | ROSE  AUC=0.743 | SMOTE  AUC=0.736 |
| Unbalanced  AUC = 0.746 | P=0.188375 | P=0.830235 | P=0.897516 | P=0.795867 | P=0.234282 | P=0.417998 | P=0.897516 | P=0.668724 |
| Oversampled  AUC = 0.753 | P=0.105684 | P=0.605575 | P=0.666686 | P=0.965463 | P=0.135751 | P=0.266138 | P=0.666686 | P=0.465452 |
| ROSE  AUC = 0.743 | P=0.235425 | P=0.931785 | P=1.000000 | P=0.69846 | P=0.288917 | P=0.495872 | P=1.000000 | P=0.764864 |
| SMOTE  AUC = 0.740 | P=0.290101 | P=0.965861 | P=0.897833 | P=0.605924 | P=0.351337 | P=0.580537 | P=0.897833 | P=0.86446 |

*p-values derived by applying Hanley and McNeil test for comparison of ROC curves.
